# Supplementary material for: Contribution of physician assistants/associates to secondary care: a systematic review
Source: BMJ Open. 2018 Jun 19;8(6):e019573. doi: 10.1136/bmjopen-2017-019573 (PMC6020983; doi:10.1136/bmjopen-2017-019573)
Supplement: Supplementary file 1 [file bmjopen-2017-019573supp001.pdf]

SUPPLEMENTARY FILE 1: Scoping review (Preliminary Medline search strategy – 24/11/2015)

#	Concept		Search Terms	Results
1	Physician Associates		exp Physician Assistants/	2410
2			exp Pediatric Assistants/	26
3			Physician Assistant$.tw.	1498
4			Feldsher$.tw.	17
5			Clinical Officer$.tw.	135
6			Paramedical Practitioner&.tw.	0
7			Medical Assistant$.tw.	324
8			Allied Health Personnel.tw.	48
9			physician associate$.tw.	37
10			(mid level adj3 provider$).tw.	124
11			((assistant* or technician* or officer* or associate$) adj2 (physician$ or surgical or clinical$ or practitioner$ or medical$ or provider$)).tw.	24985
12	Secondary Care	Emergency Medicine	exp Emergency Medicine/ and (speciali?ed or specialty or hospital$ or secondary or care or medicine).tw.	4983
13			((accident and emergency) or A&E department or emergency department or casualty or emergency Medicine).tw.	47842
14			(emergency adj3 (medic* or servic* or ward* or department)).tw.	54262
15			(exp critical care/ or exp intensive care/) and (speciali?ed or specialty or hospital$ or secondary or care or medicine).tw.	23791
16			((intensive adj3 care) and (speciali?ed or specialty or hospital$ or secondary or care or medicine)).tw.	71552
17		Acute Medicine	exp Internal Medicine/ and (speciali?ed or specialty or hospital$ or secondary or care or medicine).tw.	16968
18			(internal medicine and (speciali?ed or specialty or hospital$ or secondary or care or medicine)).tw.	10752
19			(Acute Medicine or acute internal medicine or acute medical unit$ or medical assessment unit$ or acute ward$).tw.	690
20		Trauma or Orthopaedics	(exp Orthopedics/ or exp Traumatology/) and (speciali?ed or specialty or hospital$ or secondary or care or medicine).tw.	3015
21			((Trauma or Orthop?dic$) adj3 (speciali?ed or specialty or hospital$ or secondary or care or medicine)).tw.	7280
22			(Orthop?dic surgery or trauma surgery).tw.	4466
23			((bone$ or joint$ or ligament$ or tendon$ or muscle$ or nerve$) adj3 (operation$ or surgery or replacement$)).tw.	13668
24		Care of the Elderly	(exp geriatrics/ or Aging/ or exp Aged/ or older people.mp. or exp Frail Elderly/) and (speciali?ed or specialty or hospital$ or secondary or care).tw.	361294
25			((Older adult or Aged or elderly or geriatric* or older people* or ag?ng) adj3 (speciali?ed or specialty or hospital$ or secondary or care or medicine)).tw.	15561

26			or/12-25	508965
27			exp Primary Health Care/ or exp preventive medicine/ or exp physicians, Primary Care/	75166
28			(primary care or primary healthcare or primary health care or primary health service$).tw.	68593
29			27 or 28	111510
30		**Primary care**	exp Family Practice/ or exp Physicians, Family/ or exp General Practitioners/ or exp General Practice/	47498
31			(family practice$ or family practitioner$ or family physician$ family medicine$ or General practice$ or General practitioner$ or GPs).tw.	47129
32			30 or 31	72038
33			29 not 32	91680
34		**Outpatient and inpatient care**	(exp Outpatients/ or Outpatient Clinics, Hospital/ or ambulatory care/) and (speciali?ed or specialty or hospital$ or secondary or care or medicine).tw.	18427
35			(exp Inpatients/ or Hospitalization/) and (speciali?ed or specialty or hospital$ or secondary or care or medicine).tw.	49797
36			(ambulatory care or ambulatory emergency care).tw.	3948
37			((outpatient$ or out-patient$) adj3 (speciali?ed or specialty or hospital$ or secondary or care or medicine)).tw.	11455
38			((inpatient$ or in-patient$) adj3 (speciali?ed or specialty or hospital$ or secondary or care or medicine)).tw.	24157
39	**Impact**		Treatment Outcome/ or "Outcome and Process Assessment (Health Care)"/ or "Outcome Assessment (Health Care)"/ or Medical Audit/ or Program Evaluation/	769470
40			exp Patient Readmission/ or exp Length of Stay/ or exp Clinical Audit/ or exp Medical Audit/	68267
41			Health Planning/ and (organi?ation* or system* or hospital* or Physician* or workforce or staff or professional*).tw.	2686
42			Efficiency, Organizational/ and (organi?ation* or system* or hospital* or Physician* or workforce or staff or professional*).tw.	8952
43			Resource Allocation/ and (organi?ation* or system* or hospital* or Physician* or workforce or staff or professional*).tw.	1377
44			Health Personnel/ and (organi?ation* or system* or hospital* or Physician* or workforce or staff or professional*).tw.	11958
45			Health Manpower/ and (organi?ation* or system* or hospital* or Physician* or workforce or staff or professional*).tw.	2123
46			Medical Staff/ and (organi?ation* or system* or hospital* or Physician* or workforce or staff or professional*).tw.	899
47			Delivery of Health Care/ and (productivity or efficiency or performance or guideline* or quality).tw.	8411
48			((equity or difference$ disparit$ or inequalit$ or inequit$) adj5 (experience$ or perception$ or view$ or rates or rating or review or audit or impact or influence or effect or outcome or performance or quality)).tw.	2048
49			((Acceptability or compassion or dignity or satisfaction or dissatisfaction) adj5 (experience$ or perception$ or view$ or rates or rating or review or audit or impact or influence or effect or outcome or performance or quality)).tw.	16604

50		((Efficiency or productivity or economic$ or benefit) adj5 (experience$ or perception$ or view$ or rates or rating or review or audit or impact or influence or effect or outcome or performance or quality)).tw.	34565
51		((Effectiveness or efficacy or effectivity or capability) adj5 (experience$ or perception$ or view$ or rates or rating or review or audit or impact or influence or effect or outcome or performance or quality)).tw.	35758
52		((Effectiveness or efficacy or effectivity or capability) adj5 (experience$ or perception$ or view$ or rates or rating or review or audit or impact or influence or effect or outcome or performance or quality)).tw.	35758
53		((Access$ or responsiveness or timely or timeliness) adj5 (experience$ or perception$ or view$ or rates or rating or review or audit or impact or influence or effect or outcome or performance or quality)).tw.	16251
54		((Appropriate$ or relevance or relevant) adj5 (experience$ or perception$ or view$ or rates or rating or review or audit or impact or influence or effect or outcome or performance or quality)).tw.	32405
55		((Cost$ or afford$ value for money or financ$) adj5 (experience$ or perception$ or view$ or rates or rating or review or audit or impact or influence or effect or outcome or performance or quality)).tw.	33373
56	**Impact in Secondary Care of Physician Associates**	or/1-11	26515
57		26 or 33 or 34 or 35 or 36 or 37 or 38	621770
58		or/39-55	959419
59		56 and 57 and 58	1575
60		limit 59 to (english language and last 20 years)	1513

SUPPLEMENTARY FILE 1 continued: MeSH --Medical Subject Headings definition of search terms used

(alphabetical [US spellings])

http://www.ncbi.nlm.nih.gov/mesh

Aged: A person 65 through 79 years of age. For a person older than 79 years, AGED, 80 AND OVER is available. Year introduced: 1966. By exploding this term, we do include MeSH terms found below it in the MeSH hierarchy as follows: Aged, 80 and over; Frail Elderly.

Aging: The gradual irreversible changes in structure and function of an organism that occur as a result of the passage of time. By exploding this term, we do include MeSH terms found below it in the MeSH hierarchy as follows: Longevity.

Ambulatory care: Health care services provided to patients on an ambulatory basis, rather than by admission to a hospital or other health care facility. The services may be a part of a hospital, augmenting its inpatient services, or may be provided at a free-standing facility. Year introduced: 1968(1966)

Behavioral Disciplines and Activities: The specialties in psychiatry and psychology, their diagnostic techniques and tests, their therapeutic methods, and psychiatric and psychological services. Year introduced: 1998

Clinical Audit: A detailed review and evaluation of selected clinical records by qualified professional personnel to improve the quality of patient care and outcomes. The clinical audit was formally introduced in 1993 into the United Kingdom's National Health Service. Year introduced: 2008

Critical Care: Health care provided to a critically ill patient during a medical emergency or crisis. Year introduced: 1975

Emergency medicine: The branch of medicine concerned with the evaluation and initial treatment of urgent and emergent medical problems, such as those caused by accidents, trauma, sudden illness, poisoning, or disasters. Emergency medical care can be provided at the hospital or at sites outside the medical facility.

Family Practice: A medical specialty concerned with the provision of continuing, comprehensive primary health care for the entire family.

Frail Elderly: Older adults or aged individuals who are lacking in general strength and are unusually susceptible to disease or to other infirmity. Year introduced: 1991

General Practice: Patient-based medical care provided across age and gender or specialty boundaries. Year introduced: 2011

General Practitioners: Physicians whose practice is not restricted to a specific field of medicine

Geriatrics: The branch of medicine concerned with the physiological and pathological aspects of the aged, including the clinical problems of senescence and senility.

Hospitalization: The confinement of a patient in a hospital.

Inpatients: Persons admitted to health facilities which provide board and room, for the purpose of observation, care, diagnosis or treatment.

Intensive care: Advanced and highly specialized care provided to medical or surgical patients whose conditions are life-threatening and require comprehensive care and constant monitoring. It is usually administered in specially equipped units of a health care facility. Year introduced: 1992

Internal Medicine: A medical specialty concerned with the diagnosis and treatment of diseases of the internal organ systems of adults. By exploding this term, we do include MeSH terms found below it in the MeSH hierarchy as follows: Cardiology; Cardiac electrophysiology; Endocrinology; Gastroenterology; Hematology; Transfusion Medicine; Infectious Disease Medicine; Medical Oncology Radiation; Oncology; Nephrology; Pulmonary Medicine; Rheumatology; Sleep Medicine Specialty.

Medical Audit: A detailed review and evaluation of selected clinical records by qualified professional personnel for evaluating quality of medical care. Year introduced: 1968

Mental Disorders: Psychiatric illness or diseases manifested by breakdowns in the adaptational process expressed primarily as abnormalities of thought, feeling, and behavior producing either distress or impairment of function. Year introduced: use pre-explosion 1974-1997

Mental Health Services: Organized services to provide **mental health** care. Year introduced: 1967
Mental Health: The state wherein the person is well adjusted. Year introduced: 1967

Orthopedics: A surgical specialty which utilizes medical, surgical, and physical methods to treat and correct deformities, diseases, and injuries to the skeletal system, its articulations, and associated structures.

Outcome and Process Assessment (Health Care): Evaluation procedures that focus on both the outcome or status (OUTCOMES ASSESSMENT) of the patient at the end of an episode of care - presence of symptoms, level of activity, and mortality; and the process (ASSESSMENT, PROCESS) - what is done for the patient diagnostically and therapeutically. Year introduced: 1979. By exploding this term, we do include MeSH terms found below it in the MeSH hierarchy as follows: Outcome Assessment (Health Care); Patient Outcome Assessment; Treatment Outcome; Process Assessment (Health Care)

Outcome Assessment (Health Care): Research aimed at assessing the quality and effectiveness of health care as measured by the attainment of a specified end result or outcome. Measures include parameters such as improved health, lowered morbidity or mortality, and improvement of abnormal states (such as elevated blood pressure). Year introduced: 1992

Outpatient Clinics, Hospital: Organized services in a hospital which provide medical care on an outpatient basis. Year introduced: 1978

Outpatients: Persons who receive ambulatory care at an outpatient department or clinic without room and board being provided. Year introduced: 1991(1980)

Pediatric Assistants: Persons academically trained to provide medical care, under the supervision of a physician, to infants and children. Year introduced: 1991(1975)

Physician Assistants: Health professionals who practice medicine as members of a team with their supervising physicians. They deliver a broad range of medical and surgical services to diverse populations in rural and urban settings. Duties may include physical exams, diagnosis and treatment of disease, interpretation of tests, assist in surgery, and prescribe medications. (from http://www.aapa.orglabout-pas accessed 2114/2011) Year introduced: 1995

Physicians, Family: Those physicians who have completed the education requirements specified by the American Academy of Family Physicians. Year introduced: 1974(1972)

Physicians, Primary Care: Providers of initial care for patients. These PHYSICIANS refer patients when appropriate for secondary or specialist care. Year introduced: 2011

Preventive medicine: A medical specialty primarily concerned with prevention of disease (PRIMARY PREVENTION) and the promotion and preservation of health in the individual. By exploding this term, we do include MeSH terms found below it in the MeSH hierarchy as follows: Environmental Medicine; Occupational Medicine; Preventive Psychiatry.

Primary Health Care: Care which provides integrated, accessible health care services by clinicians who are accountable for addressing a large majority of personal health care needs, developing a sustained partnership with patients, and practicing in the context of family and community. (JAMA 1995;273(3):192) Year introduced: 1974(1972).

Program Evaluation: Studies designed to assess the efficacy of programs. They may include the evaluation of cost-effectiveness, the extent to which objectives are met, or impact. Year introduced: 1989. By exploding this term, we do include MeSH terms found below it in the MeSH hierarchy as follows: benchmarking.

Psychiatry: The medical science that deals with the origin, diagnosis, prevention, and treatment of mental disorders.

Traumatology: The medical specialty which deals with wounds and injuries as well as resulting disability and disorders from physical traumas.

Treatment Outcome: Evaluation undertaken to assess the results or consequences of management and procedures used in combating disease in order to determine the efficacy, effectiveness, safety, and practicability of these interventions in individual cases or series. Year introduced: 1992

SUPPLEMENTARY FILE 2: DEFINITIONS used in this review

As this review question contained broad terms, these were defined at the outset, as follows:

- Physician Associates: trained in a medical model to work in all settings and undertake physical examinations, investigations, diagnosis, treatment, and prescribe within their scope of practice as agreed with their supervising doctor.[1,2] Physician Associates are sometimes described within the term 'mid-level providers' in developed economies: '…..the term mid-level practitioner means an individual practitioner, other than a physician, dentist, veterinarian, or podiatrist, who is licensed, registered, or otherwise permitted by the United States or the jurisdiction in which he/she practices, to dispense a controlled substance in the course of professional practice. Examples of mid-level practitioners include, but are not limited to, health-care providers such as nurse practitioners, nurse midwives, nurse anaesthetists, clinical nurse specialists and physician assistants who are authorized to dispense controlled substances by the state in which they practice.' [3] While this term is contested as an appropriate umbrella term due to its hierarchical connotations [4,5] and international variation in usage,[6] it appears in the literature regarding Physician Associates.
- Impact: using the broad headings of the components of quality as suggested by Maxwell (1992),[7] augmenting that of Donabedian,[8] that is, effectiveness, efficiency, acceptability, access, equity and relevance; further consolidated in the aspects of quality set out in the NHS Next stage Review (2008)[9]: patient safety, patient experience and effectiveness of care.
- Specialties most frequently employing PAs in England:
  - acute medicine

    'Acute medicine is the part of general (internal) medicine concerned with the immediate and early specialist management of adult patients who present to, or from within, hospitals as urgencies or emergencies'.[10]
  - care of the elderly

    '…geriatric medicine is mainly concerned with people over the age of 75, although many 'geriatric' patients are much older. However, geriatric medicine in the UK is broadly from the age of 65 onwards. Frail older people are those with multiple diseases, that often includes dementia, with reduced functional reserve who tend to present to hospital with 'geriatric syndromes' such as falls, confusion and immobility.'[11]
  - emergency medicine

    'Emergency medicine is a field of practice based on the knowledge and skills required for the prevention, diagnosis and management of acute and urgent aspects of illness and injury affecting patients of all age groups with a full spectrum of episodic undifferentiated physical and behavioural disorders; it further encompasses an understanding of the development of

prehospital and in hospital emergency medical systems and the skills necessary for this development.' [12]

– mental health /psychiatry

'Mental health problems can take many forms including depression, schizophrenia, eating disorders, anxieties, phobias, drug and alcohol abuse, post-traumatic stress disorder, and dementia.'[13] Psychiatry includes the sub specialties of child and adolescent, forensic, general adult, old age, psychotherapy and psychiatry of learning disabilities. [14]

- trauma and orthopaedics

Trauma and orthopaedics is an area of surgery concerned with injuries and conditions that affect the musculoskeletal system (the bones, joints, ligaments, tendons, muscles and nerves).[15]

References

1. Department of Health. The Competence and Curriculum Framework for the Physician Assistant. London: Department of Health 2006.
2. Physician Assistant Managed Voluntary Register The Revised Competence and Curriculum Framework for the Physician Assistant. 2012 Accessed at http://www.ukapa.co.uk/files/CCF-27-03-12-for-PAMVR.pdf.
3. United States Department of Justice. (n.d.). Mid-level practitioners authorization by state. Retrieved from http://www.deadiversion.usdoj.gov/drugreg/practioners/index.html
4. Bishop CS. Advanced Practitioners Are Not Mid-Level Providers. Vol 3 No 5 Sep/Oct 2012 287-8. J Adv Pract Oncol AdvancedPractitioner.com http://www.advancedpractitioner.com/media/152572/287.pdf
5. Pappas MD. Stop calling nurse practitioners mid-level providers. 2014 Kevin MD.com. http://www.kevinmd.com/blog/2014/07/stop-calling-nurse-practitioners-mid-level-providers.html. Accessed 24 November 2015.
6. Brown A, Cornetto G, Cumbi A, de Pinho H, Kamwendo F, Lehmann U et al. Mid-level health providers: a promising resource. Rev Peru Med Exp Salud Publica. 2011; 28(2): 308-15.
7. Maxwell R. Dimensions of quality revisited: from thought to action. Quality in Health Care 1992:1:171-177.
8. Donabedian A. The quality of care. How can it be assessed? JAMA 1988;260:1743–8. http://dx.doi.org/10.1001/jama.1988.03410120089033

9. Department of Health. High Quality Care For All NHS Next Stage Review Final Report. 2008. https://www.gov.uk/government/uploads/system/uploads/attachment_data/file/228836/7432.pdf Accessed 10 December 2015.
10. Royal College of Physicians. *Acute medical care; the right person, in the right setting—first time.* Report of a working party. London: RCP, 2007.
11. Conroy S, Cooper N. Executive summary. British Geriatrics Society, 2012. http://www.bgs.org.uk/index.php/topresources/publicationfind/goodpractice/44-gpgacutecare%20 Accessed 24 Nov 2015.
12. International Federation for Emergency Medicine. About IFEM IFEM Definition of Emergency Medicine. 2008. http://www.ifem.cc/About_IFEM.aspx Accessed Nov 24 2015.
13. Royal College of Psychiatrists. Glossary of Terms Professionals involved in the care of people with mental health problems. 2015. http://www.rcpsych.ac.uk/healthadvice/moreinformation/definitions/professionalsinpsychiatry.aspx. Accessed 10 December 2015.
14. Royal College of Psychiatrists. A Career in Psychiatry. 2015. http://www.rcpsych.ac.uk/discoverpsychiatry/acareerinpsychiatry.aspx Accessed 10 December 2015.
15. NHS Choices. Trauma and Orthopaedics (Orthopeadic Surgery) 2015. http://www.nhs.uk/Conditions/orthopaedics/Pages/Introduction.aspx
