# Supplementary material for: Contribution of physician assistants/associates to secondary care: a systematic review
Source: BMJ Open. 2018 Jun 19;8(6):e019573. doi: 10.1136/bmjopen-2017-019573 (PMC6020983; doi:10.1136/bmjopen-2017-019573)
Supplement: Author's manuscript [file bmjopen-2017-019573.draft_revisions.pdf]

BMJ Open is committed to open peer review. As part of this commitment we make the peer review history of every article we publish publicly available.

When an article is published we post the peer reviewers' comments and the authors' responses online. We also post the versions of the paper that were used during peer review. These are the versions that the peer review comments apply to.

The versions of the paper that follow are the versions that were submitted during the peer review process. They are not the versions of record or the final published versions. They should not be cited or distributed as the published version of this manuscript.

BMJ Open is an open access journal and the full, final, typeset and author-corrected version of record of the manuscript is available on our site with no access controls, subscription charges or pay-per-view fees (http://bmjopen.bmj.com).

If you have any questions on BMJ Open's open peer review process please email info.bmjopen@bmj.com

BMJ Open

The contribution of physician assistants to secondary care: a systematic review

Journal:	*BMJ Open*
Manuscript ID	bmjopen-2017-019573
Article Type:	Research
Date Submitted by the Author:	28-Sep-2017

Complete List of Authors:	Halter, Mary; St Georges University Hospital, Faculty of Health and Social Care Sciences<br>Wheeler, Carly; Kingston University Faculty of Health Social Care and Education, Centre for Health and Social Care Research<br>Pelone, Ferruccio; Royal College of Obstetricians and Gynaecologists, National Guidelines Alliance<br>Gage, Heather; University of Surrey, School of Economics<br>de Lusignan, Simon; University of Surrey, Department of Health Care Management and Policy; St. Georges University of London, Division of Population Health Sciences and Education<br>Parle, Jim; University of Birmingham, Institute of Clinical Sciences<br>Grant, Robert; Kingston University Faculty of Health Social Care and Education, Centre for Health and Social Care Research<br>Gabe, Jonathan; Royal Holloway, University of London, Criminology and Sociology<br>Nice, Laura; University of Birmingham, Institute of Clinical Sciences<br>Drennan, Vari; Kingston University Faculty of Health Social Care and Education, Centre for Health and Social Care Research

Keywords:	GENERAL MEDICINE (see Internal Medicine), Quality in health care < HEALTH SERVICES ADMINISTRATION & MANAGEMENT, ORTHOPAEDIC & TRAUMA SURGERY, Physician Assistant

SCHOLARONE™
Manuscripts

The contribution of physician assistants to secondary care: a systematic review

Authors

Corresponding author

Halter M, PhD

Faculty of Health, Social Care and Education

Kingston University and St George's, University of London

Cranmer Terrace

London SW17 0RE

00 44 (0)20 8725 0337

m.halter@sgul.kingston.ac.uk

ORCID: 0000-0001-6636-0621

Wheeler C, PhD

Faculty of Health, Social Care and Education, Kingston University and St George's, University of London, London UK

Pelone F, PhD

National Guideline Alliance, Royal College of Obstetricians and Gynaecologists, London UK

For peer review only

For peer review only - http://bmjopen.bmj.com/site/about/guidelines.xhtml

Gage H, PhD

School of Economics, University of Surrey, Guildford, UK

de Lusignan S, MD, FRCGP

Department of Clinical and Experimental Medicine, University of Surrey, Guildford, UK

Parle J, MD, FRCGP

Institute of Clinical Sciences, University of Birmingham, Birmingham, UK

Grant R, MSc

Faculty of Health, Social Care and Education, Kingston University and St George's, University of London, London UK

Gabe J, PhD

Centre for Criminology & Sociology/ Centre for Public Services and Policy, Royal Holloway, University of London, Egham, UK

Nice L, PhD

Institute of Clinical Sciences, University of Birmingham, Birmingham, UK

For peer review only

For peer review only - http://bmjopen.bmj.com/site/about/guidelines.xhtml

Drennan VM, PhD

Faculty of Health, Social Care and Education, Kingston University and St George's, University of London, London UK

Word count: 4837

For peer review only

For peer review only - http://bmjopen.bmj.com/site/about/guidelines.xhtml

ABSTRACT

Objective: to appraise and synthesise research on physician assistants/associates' impact in acute, care of the elderly and emergency medicine; trauma and orthopaedics and mental health.

Design Systematic review

Setting: Electronic databases (Medline, Embase, ASSIA, CINAHL, SCOPUS, PsycINFO, Social Policy and Practice, EconLit and Cochrane database), reference lists and related articles.

Included articles: Peer reviewed articles of any study design, published in English, 1995 to 2015.

Interventions: Blinded parallel processes were used for screening abstracts and full text, data extractions and quality assessments against published guidelines. A narrative synthesis was undertaken.

Outcome measures: Impact on patients' experience and outcomes, service organisation, working practices, other professional groups and costs.

Results: 4267 references were identified and 127 read in full; 11 were included - emergency medicine (six), trauma and orthopaedics (four), internal (acute) medicine (one) and care of the elderly or mental health (none). All studies were observational, with variable methodological quality.

In emergency medicine and trauma and orthopaedics, when PAs are added to teams, reduced waiting and process times, lower charges and acceptability to staff and patients are reported. Analgesia prescribing, operative complications and mortality outcomes were variable. In internal medicine outcomes of care provided by PAs and doctors were equivalent.

Conclusions: The review suggests PAs can be used well to increase the capacity of a team, enabling time, throughput, continuity and medical cover gains. When comparing PAs to medical staff reassuringly little or no effect on health outcomes or cost is observed. The difficulty of attributing cause and effect in complex systems where work is organised in teams is highlighted. Rigorous

For peer review only

For peer review only - http://bmjopen.bmj.com/site/about/guidelines.xhtml

evaluation is required to addresses the complexity of the PA role, reporting on more than one setting and including comparison between PAs and roles for which they are substituting.

Strengths and limitations of this study

- This study systematically analyses the empirical evidence for the contribution of physician associates to secondary care, following international guidelines
- It focuses on specialties in which physician associates are increasingly deployed in the UK, while aiming for international applicability.
- It highlights the limitations in quality in the current literature, but presents a picture for clinical decision makers of where physician associates could add value.

For peer review only

For peer review only - http://bmjopen.bmj.com/site/about/guidelines.xhtml

THE CONTRIBUTION OF PHYSICIAN ASSISTANTS TO SECONDARY CARE: A SYSTEMATIC REVIEW

Introduction

Health care systems internationally face medical workforce challenges. [1] An approach used in many countries has been to develop of advanced clinical practitioner roles (also sometimes known as mid-level non-physician clinicians), who undertake some of the activities of doctors. [2] One of these roles is the physician assistant. Physician assistants (PAs), were first developed, by physicians, in the 1960s in the United States (US) in response to medical shortages in certain specialties and regions.[3] Today approximately 93,000 PAs practice in the US[4] as nationally certified and state-licensed medical professionals in healthcare teams with physicians and other providers in all 50 states[5] Over the last two decades other countries have been introducing PAs into their health workforce, including Australia, Canada, Germany, Ghana, India, Kenya, the Netherlands, Saudi Arabia, South Africa, Taiwan, and the UK,[6] where they are known as physicians associates. Some countries have national or federal policy commitments to develop PA education programmes and significantly increase their availability,[7,8] while others are determining the value of such roles through demonstration projects.[9] The majority of PAs are employed in hospital settings.[10-12] However, like many aspects of workforce innovation and change, there is very limited published evidence as to the contribution and impact PAs have within this setting.

Existing systematic reviews of the contribution PAs make to health care have consider evidence from primary and secondary caretogether [13] just primary care, [14] or rural healthcare and emergency department [15] with no publications included after 2010. Given

For peer review only - http://bmjopen.bmj.com/site/about/guidelines.xhtml

the recent trends to utilise PAs internationally in secondary care, our purpose in conducting this new review was to fill this gap in current evidence.

The objective of the review was to appraise and synthesise the published literature on the impact of physician associates on patient experience and outcomes, service organisation, working practices, other professional groups and cost. The review was bounded by consideration of the secondary care specialties in which PAs were most frequently reported as employed in the UK. Using the annual 2016 UK Association of Physician Associates Census (n=150 PA respondents),[16] four specialties with relatively larger numbers of PAs replying to the survey were clearly identifiable: acute medicine (n=23), emergency medicine (n=23), care of the elderly (n=12) and trauma and orthopaedics (n=10). While three other specialties (cardiology, neurology and general surgery) reported five PAs in each, we selected mental health as our fifth specialty to explore, with four PAs reported,[16] to provide a contrast to the focus on physical health in the other four specialties selected. The concentration of PAs in these clinical areas is consistent with evidence from other European countries developing a PA workforce.[17] The review is intended to inform clinicians and managers considering innovation and change in their secondary care workforce.

METHODS

Search strategy

This systematic review was designed and reported to meet international guidelines: the Preferred Reporting Items for Systematic Reviews and Meta-Analyses (PRISMA).[18] Full details of the overall search strategy can be found in the research protocol, registered with

For peer review only - http://bmjopen.bmj.com/site/about/guidelines.xhtml

International Prospective Register of Systematic Reviews (PROSPERO), CRD42016032895.[19]

Studies addressing the research question were identified by systematic searching for keywords in the following electronic databases: Medline (Ovid), Embase (Ovid), Applied Social Sciences Index and Abstracts (ASSIA), Cumulative Index to Nursing and Allied Health Literature (CINAHL) Plus (EBSCO), SCOPUS –V.4 (Elsevier), PsycINFO, Social Policy and Practice (Ovid), EconLit (EBSCO), and Cochrane Central Register of Controlled Trials (CENTRAL) from the beginning of January 1995 to the 2nd week of December 2015. No language or publication status restrictions were imposed at the electronic search strategy stage. We present the Medline search strategy, and the definitions of the MeSH terms employed, in Supplementary file 1.

In addition, we used 'lateral searching' techniques[20] including checking reference lists of systematic reviews identified at the abstract screening stage and papers selected for inclusion after full text reading; using the 'Cited by' option on Scopus, and the 'Related articles' option on PubMed and tracking citations.

Inclusion criteria and study selection

Relevant studies were selected according to eligibility criteria using a two-step screening process: 1) title and abstract screening; and 2) full-text screening. First, two authors (CW and FP) in parallel sifted titles and abstracts of all the articles resulting from the searches to ascertain their potential relevance, with disagreements resolved by a third author (MH or VMD). All the full-texts of the potentially relevant citations were further examined in parallel by two authors (pairings amongst CW, FP, or MH) to analyse whether they met all the inclusion criteria. Disagreements were resolved by peer discussion and a third view from the project lead (VMD) if required.

For peer review only - http://bmjopen.bmj.com/site/about/guidelines.xhtml

Peer-reviewed articles were considered for analysis if they fitted the following inclusion criteria:

- Population: Physician Associates (PAs) according to the UK definition [21]
- Intervention: The implementation of PAs in the following secondary health care specialties: acute medicine, care of the elderly, emergency medicine, mental health, and trauma and orthopaedics (see supplementary file 2 for the definitions used).
- Comparison: The comparison group was any health care professional to whom PAs were compared.
- Outcome: Any measure of impact, informed by recognised dimensions of quality - effectiveness, efficiency, acceptability, access, equity and relevance.[22]
- Study design: Any study design that allowed measurement of impact of PAs in a primary study.

Screening exclusion criteria

Articles were excluded if they did not fulfil one or more inclusion criteria or if they: 1) were not published in the English language, 2) reported on PAs working in countries that are not defined by the International Monetary Fund as advanced economies;[22] 3) did not report empirical findings or were published only in abstract form; 4) presented their results for PAs in an amalgamated form with the results for other professions/mid-level providers or did not describe the specialties they were reporting on; 5) contained only descriptive accounts of PA demography, workload, clinical practice or productivity or PA self-report of any aspect of their role; 6) focused on and measured an intervention delivered by PAs rather than PAs as the intervention; 7) focused on and measured PA clinical practice or productivity before and after a service redesign or educational intervention; 8) focused solely on educational

For peer review only - http://bmjopen.bmj.com/site/about/guidelines.xhtml

processes; and 9) presented literature reviews, commentaries, and/or non-peer-reviewed articles.

Data collection and quality assessment

Two authors (pairings amongst FP, CW and MH) independently extracted the data from selected papers, with any disagreement resolved through discussion. A checklist was used to extract the following information from the selected papers: 1) general characteristics of studies and 2) results, limitations and conclusions as noted by authors and reviewers.

The same author pairings appraised the quality of included studies using the QualSyst quality checklists for quantitative and qualitative studies, [25] with additional questions from the Mixed Methods Appraisal Tool [26] where appropriate. For the quantitative studies, 12 items (table 3a) were scored depending on the degree to which the specific criteria were met ("yes" = 2, "partial" = 1, "no" = 0). Scores for the qualitative studies were calculated in a similar fashion, based on the scoring of ten items. Any items not applicable to a particular study design were marked "n/a" and were excluded from the calculation of the summary score. No study was excluded on the basis of its quality score; the limitations of lower quality evidence are however explored in considering how much weight can be given to the evidence when we synthesise studies. [27]

Data analysis

A meta-analysis was not performed due to the heterogeneity of the included studies in terms of scope and outcomes investigated. Therefore, narrative synthesis was undertaken [28] conducted against the four elements in guidance on the conduct of narrative synthesis in systematic reviews [29, 30]: developing a theory of how the intervention works, why and for whom; developing a preliminary synthesis of findings of included studies; exploring

For peer review only - http://bmjopen.bmj.com/site/about/guidelines.xhtml

relationships within and between studies; assessing the robustness of the synthesis (through formal quality assessment as well as reflection). For the synthesis the included studies were grouped into specialty (that is, acute medicine, care of the elderly, emergency medicine, mental health and trauma and orthopaedics) and then sub-grouped into the outcomes they measured.

RESULTS

Search results

The overall search strategy identified 4,267 references, from which we selected 136 articles for more detailed reading. Figure 1 presents the PRISMA flowchart, illustrating the literature search and selection process, and reasons for study exclusion on full text reading. A total of 11 articles were included for data collection, quality appraisal and data analysis.

A summary of the included evidence is presented below in three subsections: characteristics of included studies, methodological quality, and synthesis of findings on the impact of PAs.

Characteristics of included studies

Table 1 presents the characteristics for each study in terms of the specialties they were drawn from.

For peer review only

For peer review only - http://bmjopen.bmj.com/site/about/guidelines.xhtml

220 **Table 1: Characteristics of studies included in full – studies presenting comparisons of PAS with other health care professionals**

221

Specialty	Aim(s)	Study Setting	Intervention	Comparison	Participants	Study design	Outcome measures	First author and year
Emergency medicine	To determine whether PAs are an appropriate option for providing services rendered by physicians in the ED	USA<br><br>Walk in urgent care facility (satellite of an inner-city teaching hospital level 1 trauma centre)	PAs (n=5) rotate from the ED. PAs work solo from 08.00- 12.00. No written diagnostic or therapeutic guidelines were followed.	25 physicians rotate from the ED. Physicians work solo from 17.00-21.00. No written diagnostic or therapeutic guidelines were followed.	n= 5345 (seen by PAs)<br>n = 4256 (seen by physicians) during times of single coverage June 1995-June 1996	Comparative<br><br>retrospective	• Length of visit<br>• Total charge	Arnopolin 2000[31]
Emergency medicine	To examine the impact of PAs and nurse practitioners in EDs	Canada<br><br>Six community hospitals with ED volumes between 23 and 66,000	PAs were introduced as an unregulated provider without medical directives and worked under the supervision of a registered physician who was responsible for all patient care on predetermined busiest periods for each ED	Baseline two weeks	All ED patients: Baseline n=9,585; two week period six months post implementation June 2007 n=10,007, of which PAs were on duty for 1,076 visits and directly involved in n=376	Descriptive<br>retrospective	• Leaving without being seen<br>• Wait time (triage to initial assessment)<br>• LOS in ED	Ducharme 2009[32]

For peer review only - http://bmjopen.bmj.com/site/about/guidelines.xhtml

Emergency medicine	To understand trends in emergency medicine and interprofessional roles in delivering this care […] The focus was on how doctors, PAs and nurse practitioners NPs share emergency medicine visits	USA<br><br>National sample EDs of non-institutional general and short-stay hospitals in the 50 States and the District of Columbia from the National Hospital Ambulatory Medical Care Survey	PAs as providers of ED care and prescribers of medication in emergency medicine (7.9% of patients seen by PAs in 2004)	Physicians and Nurse Practitioners	Random sample of patient visits to hospital EDs (n= 1,034,758,313), 1995-2004	Longitudinal	• Proportion of visits in which medications are prescribed<br>• Mean number of prescriptions written per visit<br>• Non-narcotic analgesics prescriptions<br>• Narcotic analgesics/NSAIDS prescription by type of provider<br>• Patient contact growth by provider	Hooker 2008[33]
Emergency medicine	To compare the analgesic practices of emergency physicians with that of PAs	USA<br><br>ED within a suburban teaching hospital in Michigan with 90,000 annual visits	PAs were deployed for seeing patients presenting at the ED with isolated lower extremity trauma. PAs work closely with emergency physicians in the Prompt Care Area of the ED	Emergency physicians	n=384 survey respondents of patients of all ages who presented at the ED with an isolated lower extremity injury evaluated with a foot or ankle radiograph, n=227 PA patients, n=153 emergency physician patients in a nine week period	Prospective cohort	• Analgesia prescribing	Kozlowski 2002[34]

For peer review only - http://bmjopen.bmj.com/site/about/guidelines.xhtml

Emergency medicine	To compare the quality of ED pain management before and after implementation of the Joint Commission on the Accreditation of Healthcare Organizations' standards in 2001	USA<br><br>National sample EDs included in the National Hospital Ambulatory Medical Care Survey	The use of PAs in the care of patients presenting to the ED with a long bone fracture	Patients presenting to the ED with a long bone fracture not seen by PAs (medical residents, internists)	n=2064 Patients presenting at the ED with a long bone fracture (femur, humerus, tibia, fibula, radius, or ulna) in two time periods: 1998-2000, n= 834 of which 3% were seen by a PA, 9% by resident/intern and 90% by staff physician ; 2001-2003 8% PA, 10% resident/intern, 90% staff physician	Retrospective cohort	• Proportion of patients with long bone fracture receiving analgesia	Ritsema 2007[35]
Emergency medicine	To compare the wound care practices and infection rates of wounds managed in the ED by practitioners with varying levels of medical training.	USA<br><br>Department of Emergency Medicine within a teaching hospital in New York	All patients with lacerations were evaluated by an attending physician who determined whether wound could be managed by a junior practitioner (PAs, students, interns, and residents)	ED patients whose wounds were managed by other providers (students, interns, and residents)	All patients with lacerations attending the ED n=1163, n=901 seen by a PA, n=262 by other providers October 1992 – November 1993	Prospective observational	• Patient wound infection rate	Singer 1995[36]

For peer review only - http://bmjopen.bmj.com/site/about/guidelines.xhtml

Trauma and orthopaedics	To define the clinical and financial impact of hospital-based PAs on orthopaedic trauma care at a level II community hospital.	USA<br><br>Orthopaedic trauma care at a level II community hospital.	Hospital-employed PAs (n=2) were utilised to cover all orthopaedic trauma needs, under the supervision of one of 18 orthopaedic surgeons. Each PA performed 12-hour day shifts for three consecutive days, January to December 2007. PAs on call carried trauma pagers and reported to the emergency room as soon as possible.	Attending surgeon as the primary orthopaedic responder for emergency department consults	n=1104<br>• n=310: PA<br>• n=687: No PA	Comparative retrospective	• Triage time to time seen by orthopaedic service in emergency department (minutes)<br>• Triage time to time of surgery (minutes)<br>• Operating room complication rates (%)<br>• The use of deep vein thrombosis prophylaxis (%)<br>• Post-operative antibiotic administration (%)<br>• Postoperative complications (%)<br>• Triage time to out of emergency department (minutes)<br>• Operating room set up time (minutes)<br>• Average operating room time (minutes)<br>• Time from wound closure to wheels out (operating room) (minutes)<br>• Hospital length of stay (minutes)<br>• Cost savings (emergency department) ($)<br>• Cost savings (operating room) ($)	Althausen 2013[37]

For peer review only - http://bmjopen.bmj.com/site/about/guidelines.xhtml

222

Trauma and orthopaedics	To describe the effect of PAs working in an arthroplasty practice from the perspective of patients and health care providers<br>To describe the costs, time savings for surgeons and effects on surgical throughput and waiting times	Canada<br><br>High-volume academic arthroplasty programme employing PAs (The Concordia Joint Replacement Group)	Addition of PAs (n=3) to the operating room team. The PAs were added to the team, replacing surgical assists (usually general practitioners). The PAs took first call with their supervising physician, provided first-assist services in the operating room (OR), write postoperative orders, generate operative notes, undertake daily working rounds and complete discharge summaries.	-Costs: GP first assists in the operating room<br><br>-Waiting times: Patients on the arthroplasty waiting list in 2004 and 2005	Sample size varying by outcome:<br>-Patient satisfaction n=1070<br>-Perceptions of healthcare providers and patients n=44<br>-Costs n=402 surgical procedures performed in 2006<br>-Time savings n=1409 procedures carried out 2006<br>-Waiting times in 2006	Mixed-methods	• Patient satisfaction<br>• Perceptions of healthcare providers and patients about PAs<br>• Costs<br>• Time savings<br>• Waiting times<br>• Throughput	Bohm 2010[38]
Trauma and orthopaedics	To assess whether staffing changes within a Level 1 trauma centre improved mortality and shortened hospital and ICU length of stay for patients with trauma.	USA<br><br>Urban, community-based level I trauma centre	Group 3: core trauma panel and PAs	Group 1: general surgery residents (staffed by full-time, in-house post-graduate year-4 general surgery residents with attending back up from home, followed by a transition to a trauma service staffed with in-house independent general surgeon attendings) ;	n=15297 Trauma patients 18 years or older and not transferred from the ED to another acute care facility	Prospective cohort	• Overall mortality<br>• Mortality for patients with injury severity score (ISS) >15<br>• Hospital LOS	Mains 2009[39]

For peer review only - http://bmjopen.bmj.com/site/about/guidelines.xhtml

				Group 2: core trauma panel (consisting of full-time, in-house trauma surgeons, without PAs or residents)				
Trauma and orthopaedics	To analyze patient outcomes and efficiency of care provided for trauma patients during this transition from resident physician support to PA support	USA<br><br>Level I Trauma Center	PAs substituting for doctors in trauma alerts: PA's role was to assist the trauma surgeon at the trauma alerts and trauma patient rounds, update the trauma patient census list	General and orthopaedic residents who attend in trauma alerts	n=293-before<br>n=476-after<br>All patients evaluated by the trauma surgeons and on the trauma registry, excluding those transferred to another facility for treatment of severe burns	Before-after	• Collaborative relationship<br>• Transfer time<br>• LOS<br>• Mortality rate	Oswanski 2004[40]
Internal medicine	To examine and compare costs, between a PA service and an intern/resident (teaching) service in the provision of inpatient care for five high-volume internal medicine diagnostic related groups	USA<br><br>Two general internal medicine units, teaching hospital	The use of PAs (n=16) in the provision of care within internal medicine department (64 attending physicians on rotation coverage, scheduled to admit to either a PA or teaching service, with group assignment determined one year in advance).	The teaching service (32 intern/residents with an average experience of one year post-medical school)	Adult patients discharged in the following diagnostic-related groups: cerebrovascular accident/stroke, pneumonia, acute myocardial infarction discharged alive, congestive heart failure, gastro-intestinal haemorrhage: n=923, of which n=409 PA and n=514 teaching service	Prospective cohort study	• Relative value units (costs)<br>• Length of stay	Van Rhee 2002[41]

For peer review only - http://bmjopen.bmj.com/site/about/guidelines.xhtml

In summary, six studies were included from emergency medicine, [31-36] four studies reported from trauma and orthopaedics [37-40] and one from internal medicine. [41] No studies were identified from acute medicine, care of the elderly or mental health. The publication year ranged from 1995[36] to 2013, [37] with only two of the included studies being published after 2010. The majority were from the USA (n=9), with the other two from Canada.[32,38] The studies measured a number of outcomes (see Table 2).

For peer review only

For peer review only - http://bmjopen.bmj.com/site/about/guidelines.xhtml

Table 2: Main findings of included studies

Specialty	Outcome measures	Finding(s)	Quality score	Key limitations	Study details
Emergency medicine	Length of visit (LOV)	Small but clinically insignificant differences (regression coefficient -8): LOV was 8 minutes longer when patients were treated by a PA (mean 82 minutes) than a physician (mean 75 minutes) (95% CI -10 to -6, p<0.001), although difference ranged from 5 to 32 minute difference dependent on patient condition	82%	• Not randomised<br>• Differences by patient condition not explained<br>• Limited control for confounders	Arnopolin 2000[31]
	Total charge	Mean total charge was $159 when patients were treated by a PA and $164 by a physician (95% CI: 2 to 14, p=0.013), regression coefficient -8			
Emergency medicine	Leaving without being seen	Absolute improvement (not controlling for hospital or acuity) from 6.5 to 4.9%; when a PA was on duty, the likelihood that a patient left without being seen was less than half (44% [95% CI 31% to 63%] p < 0.01), controlling for hospital and patient acuity	73%	• Two months data<br>• Sample size unclear	Ducharme 2009[32]
	Wait time (triage to initial assessment)	When a PA was involved in patient care, the odds of the patient being seen within the benchmark wait time was 1.6 times greater than when the PA was not involved (95%CI 1.3 to 2.1) p <0.05, adjusting for hospital, acuity and time of day			
	LOS in ED	When a PA was involved in patient care, the LOS in the ED was shorter (mean: 262.4 mins versus 182.9 mins) than when a PA was not present (30.3% [95% CI 21.6% to 39%]), p < 0.01			
Emergency medicine	Proportion of visits in which medications are prescribed	Significant differences were observed between PAs if compared to physicians and NPs in the proportion of visits in which medication was prescribed: PAs 77.9%, physicians 75.5%, nurse practitioners 75.4% (p=0.001)	73%	• Secondary data analysis<br>• No adjustment<br>• Treatment outcomes/appropriateness not assessed	Hooker 2008[33]
	Mean number of prescriptions written per visit	There were no significant differences among the three providers in terms mean number of prescriptions per visit (PA and physician 1.7, nurse practitioner 1.6)			
	Non-narcotic analgesics prescriptions	There were no significant differences among the three providers in the frequency of prescribing non-narcotic analgesics (p=0.16).			
	Narcotic analgesics/NSAIDS prescription by type of provider	There were also no significant differences among the three prescribers in the frequency of narcotic analgesics or NSAIDS recorded (p=0.15 and p=0.06, respectively)			

For peer review only - http://bmjopen.bmj.com/site/about/guidelines.xhtml

Emergency medicine	Analgesia prescribing	Emergency physicians gave some form of ED analgesia to 29% of patients, as compared with 10% of patients seen by PAs (OR=3.58 [CI 95% 2.05 to 6.24]), adjusting for sex, reported degree of pain and fracture	92%	• Dependent on patient recall	Kozlowski 2002[34]
Emergency medicine	Proportion of patients with long bone fracture receiving analgesia	Patients seen by PAs had more than twice the odds of receiving opiates/narcotics (OR=2.05% [95%CI 1.24 to 3.29]) and were more likely to receive analgesics (OR=1.72% [95%CI 0.94 to 3.17]) compared with those not seen by PAs	100%	• Changes in workload and documentation could have confounded results	Ritsema 2007[35]
Emergency medicine	Patient wound infection rate	There were no significant differences in wound infection rates by practitioner level of training (medical students, 0/60[0%]; all residents, 17/547[3.1%]; physician assistants, 11/305[3.6%]; and attending physicians 14/251[5.6%]; p=0.14)	67%	• Hawthorne effect<br>• Differences in wounds not controlled for	Singer 1995[36]
Trauma and orthopaedics	Triage time to time seen by orthopaedic service (emergency department) (mins)	PA presence resulted in a 205 minutes faster orthopaedic service response time (366 versus 571 mins; p=0.0006)	91%	• Exact cost savings difficult to determine<br>• Did not have a way of calculating savings for the time it took for patients to reach the OR from the time of triage<br>• Single site with two PAs	Althausen 2013[37]
	Triage time to time of surgery (ER) (mins)	PA presence resulted in a 360 minutes improvement in time to surgery (1139 versus 1499 mins; p=0.03)			
	Operating room complication rates (%)	There was no significant difference in the proportion of operating room complications between the presence and the absence of PAs (both 0.65%; p=0.9972)			
	The use of deep vein thrombosis prophylaxis (%)	The use of deep vein thrombosis prophylaxis increased by a mean of 6.73% (60.69 versus 53.96%; p = 0.0084) with PA presence.			
	Post-operative antibiotic administration (%)	Post-operative antibiotic administration increased by 2.88% with PA presence (94.35 versus 91.47%; p=0.0302)			
	Postoperative complications (%)	There was a 4.67% decrease in postoperative complications with PA presence (8.16 versus 12.83%; p=0.0034)			

For peer review only - http://bmjopen.bmj.com/site/about/guidelines.xhtml

	Triage time to out of emergency department (mins)	There was a 176 minutes decrease in total ER time with PA presence (270 versus 446 mins; p<0.001)			
	Operating room set up time (mins)	There was a marginally improved operating room set up time by 0.43 minutes with PA presence (26.6 versus 24 mins; p=0.0034)			
	Time from wound closure to wheels out (operating room) (mins)	There was no significant difference for this outcome when the PA was present (7.8 versus 7.6 mins; p=0.5914)			
	Average operating room time (mins)	There was no significant difference in the average operating room time when the PA was present (70 versus 74 mins; p=0.44)			
	Cost savings (emergency department) ($)	Based on 50% collection of PA charges and emergency department time savings, per orthopaedic trauma patient seen, PAs saved the hospital $133.53 per patient, resulting in $41,394 in one year (310 patients)			
	Cost savings (operating room) ($)	The presence of a PA in the operating room resulted in savings of $3,207 based on operating room costs (only set up time was decreased with presence of the PA).			
	Hospital length of stay (days)	There was no significant difference in the hospital LOS when the PA was present if compared to when the presence and the absence of PAs (7.96 versus 8.57 days; p=0.2662)			
Trauma and orthopaedics	Patient satisfaction	91.3% of hip patients (total= 626, 58.5% response) reported being satisfied or very satisfied and 87.7% of knee patients reported being satisfied or very satisfied with PAs at one year follow-up (after surgery)	32%	• Methods are not fully described e.g. no description of data analysis<br>• Sample is not described<br>• Is this a study about PAs or about the two room operating model?<br>• Patient satisfaction with the surgery at one year cannot be attributed to the PA	Bohm 2010[38]
	Perceptions of healthcare providers and patients about PAs	Patients: Overall patients expressed very positive opinions of PAs who were helpful in providing information and explaining aspects of their care<br>Ward nurses: felt that patient care, information flow and patient rounds were enhanced by the PAs; ambiguous as to whether PA tasks fell within the scope of nursing<br>Orthopaedic surgeons: overall the surgeons had very positive opinions of PAs – 100% agreement with all survey items: 'a fully trained PA provides surgical assistance equal to an R5'; 'the presence of PA has improved your job satisfaction'; 'the presence of a PA has safely allowed you to do more surgical volume'; 'the care of your patients in the OR is improved by the assistance of PAs'; 'PAs greatly decrease the amount of "scut work" that you have to do'<br>Operating room nurses: overall OR nurses reported that PAs were valuable team members; improved the care of orthopaedic surgery patients in the operating room; provided surgical assistance superior to family practitioners; and were necessary to run two operating rooms			

For peer review only - http://bmjopen.bmj.com/site/about/guidelines.xhtml

		Orthopaedic residents: nearly unanimous that PAs reduced their workload and they generally felt that PAs relieved them of clinical responsibilities so that they could attend teaching.			
	Costs	The cost of employing three PAs in 2006 (between $270,000 AND $327000) was found to be similar to the forgone general practitioner (GP) surgical assist fees of $270226.88.			
	Time savings	PAs were found to “free up” 204 hours per year (the equivalent of four 50-hour work weeks), for their supervising physician (p=not reported). Furthermore, they potentially freed GPs from the operating room to spend more time delivering primary care			
	Throughput	Increased the volume from three to seven primary joint surgeries per day through the use of double rooms in 2006			
	Waiting time	Median wait time for surgery decreased from 44 to 30 weeks			
Trauma and orthopaedics	Overall mortality	The introduction of PAs to the core trauma panel (group 3 versus group 2) decreased overall mortality (2.80% versus 3.76%, adjusted OR=0.74 [CI95% 0.55 to 0.99], p=0.05). Furthermore, the introduction of PAs to general surgery residents (group 3 versus group 1) decreased overall mortality (2.32% versus 3.82%, adjusted OR=0.6 [CI95% 0.45 to 0.81], p=0.003)	100%	• Not all the covariates which could be significantly associated with outcomes were collected (e.g. changes in care)<br>• The group 1 period was characterised by a transition from on-call attending surgeons to in-house surgeons and the outcomes may not be homogenous across the study period<br>• Other changes were made, not just individual staff type	Mains 2009[39]
	Mortality for patients with injury severity score (ISS) >15	The introduction of PAs to the core trauma panel (group 3 versus group 2) decreased overall mortality for patients with injury severity score (ISS) >15 (9.67% versus 12.21%, adjusted OR=0.77 [CI95% 0.55 to 0.99], p=0.13). Furthermore, the introduction of PAs to general surgery residents (group 3 versus group 1) decreased overall mortality in this patients (9.03% versus 14.83%, adjusted OR=0.6 [CI95% 0.41 to 0.80], p=0.003)			
	Hospital LOS	The introduction of PAs to the core trauma panel (group 3 versus group 2) reduced mean and median hospital LOS (4.32 days versus 4.69 days, p=0.05; and 3.74 days versus 3.88 days, p= 0.02, respectively). As well, the introduction of PAs to general surgery residents (group 3 versus group 1) reduced mean and median hospital LOS (4.32 days versus 4.62 days, p=0.05; and 3.74 days versus 3.94 days, p= 0.003, respectively)			
Trauma and orthopaedics	Collaborative relationship	Participation during trauma alert calls: PA 100%; resident 51% overall, 88% during on duty hours; Involvement in minor procedures PA 100% when residents off-duty, 91% overall; resident 95% during on duty hours, 83% overall.	Before-after 82%	• Investigators not blinded and all work in the trauma centre investigated.<br>• No sample size calculation versus multiple<br>• Single site with two PAs<br>• Minimal description of	Oswanski 2004[40]
	Transfer time	When controlling for age, gender, race and severity of illness, there was no significant difference in the mean transfer rate overall or for any subpopulation (destination) between years 1998 and 1999			
	LOS	When controlling for age, gender, race and severity of injury, there was no significant			

For peer review only - http://bmjopen.bmj.com/site/about/guidelines.xhtml

		difference in the mean LOS overall between years 1998 and 1999		data collection method	
	Mortality rate	Mortality rate for all patients admitted to the trauma service was 2.2% for both 1998 (8/293) and 1999 (13/479)			
Internal medicine	Relative value units (costs)	1) Radiology RVUs: There were no statistically significant differences between PAs and residents; 2) Total RVUs (excluding pharmacy data): PAs used significantly fewer resources when compared to resident services for pneumonia care (p = .004), although had a higher mortality rate (% and p value not reported). For all other diagnoses there were no statistically significant differences in total relative value units between PAs and residents; 3) Laboratory RVUs: There were statistically significant differences between PAs and residents in laboratory relative value units for stroke (p = .015), pneumonia (p = .003) and CHF (p = .004). In each case PAs' RVUs were lower than those of residents.	Prospective cohort study 86%	• RVU figures are not explained<br>• Non-random group assignment<br>• Single centre	Van Rhee 2002[41]
	LOS	There were no significant differences in LOS between PAs and residents after adjusting for admitting physician effect and other covariates			

For peer review only - http://bmjopen.bmj.com/site/about/guidelines.xhtml

One study employed mixed methods; [38] the remainder employed quantitative approaches. Five studies analysed prospectively collected data [34,36,38,39,41] and four used a retrospective analysis.[31,32,33,37] All studies bar one [41] were observational.

Methodological quality

The studies were of variable methodological quality. The mean score was 80% (SD 19), median 73%, minimum 32%, [38] maximum 100%, [35,39] IQR 73,92. Figure 2 presents a summary of the degree to which the included studies met the criteria of methodological quality and shows that the most important methodological flaws in the included studies were the failure to adjust the analysis for confounding variables, the absence of information to evaluate participants' selection adequacy, and the lack of information about baseline and/or demographic information of the investigated patients or PAs.

Synthesis of findings on the impact of physician associates

We organised our findings by secondary care specialty. Within each specialty, we described the findings within the quality dimensions, [20] presenting the dimension with the largest number of studies within each specialty.

Emergency medicine

The six studies in emergency medicine compared clinical care offered by PAs and physicians of various grades[33,34,35,36] and two operational/service measures.[31,32] In only one of these studies was the comparison of PAs and other physicians in a system where the PAs were described as working 'solo', substituting for physicians at particular times of the day.[31]

For peer review only - http://bmjopen.bmj.com/site/about/guidelines.xhtml

Waiting or access_outcomes were reported in one Canadian study; [32] the outcomes were leaving without being seen and waiting times. The presence of a PA was reported as significantly reducing the likelihood of a patient leaving without being seen by 44% (the crude rate being 6.5 without and 4.9% with a PA).   and the odds of a patient being seen within their benchmark wait time was 1.6 times greater when the PA was involved in the patient's care, with these analyses strengthened by adjustment for hospital, time of patient visit and acuity level.[32] However, the PA was an additional staff resource rather than a substitute in this study, giving extra coverage at the busiest times, alongside also newly appointed nurse practitioners, who increased the odds of being seen on target more than the PAs did, with an odds ratio of 2.1. .

Length of stay was considered in two studies,[31,32] with contradictory results in the comparison against physicians, from different interventions in terms of PAs. Arnopolin and Smithline (2000)[31] reported experienced ED PAs and physicians working solo at different times of day in a satellite unit. This study provided a direct comparison (and control for patient age in the analysis), with a result of a statistically significantly mean longer length of visit (eight minutes) for patients of PAs but also noted that differences in length of visit varied by diagnostic group, with PAs' patients between five and 32 minutes longer. In contrast, Ducharme et al[32] reported that where   PAs were an additional staff resource alternating with nurse practitioners, PAs reduced length of stay the by 30% (mean 80 minute reduction).

Cost was considered through total charge (hospital and physician charge) for the visit, [31] with a small but statistically significant decrease per patient reported when patients were treated by a PA, with differences (not statistically significant) by diagnostic groups.

For peer review only - http://bmjopen.bmj.com/site/about/guidelines.xhtml

Treatments offered, in terms of analgesia prescribing, were reported in three studies, [33,34,35] with conflicting findings. Secondary analysis of national (USA) ED survey data (1995 to 2004) reported no significant difference by type of provider in frequency of prescribing narcotic and non-narcotic analgesics and in the mean number of prescriptions per visit, but did observe a statistically significantly higher proportion of PAs' cases receiving a prescription compared with those of physicians and nurse practitioners. [33] No adjustment for potential confounders was made. Using the same national survey data but for a subset for long bone fractures, secondary analysis for 1998 to 2003 reported similarly, with those seen by a PA having adjusted odds of 2.05 for receiving opiate analgesia in the ED. This well powered retrospective cohort study of high quality differs from another study of similar quality with somewhat contrasting findings [34]. For patients contacted at an undefined time, on average three days following their ED visit, those attended by an emergency physician had adjusted odds of 3.52 for receiving pain medication while in the ED (29% of their patients) compared to those attended by PAs (10% of their patients), in a prospective cohort study based on patient self-report.[34] Although the period of time for this study is not specified, it first reported in 1998, perhaps suggesting the same decade of data was involved. These three studies did not report the PAs' place in the team or whether they added to, substituted for members of the medical team, nor whether they saw patients as part of a team or solo.

The only study that considered a clinical outcome of care was the oldest study in the review [36], from 1995. PAs were reported to have no statistically significant difference in wound infection rates, in a large sample of patients presenting with lacerations at the ED, compared to other medical staff providers (medical students, residents and attending physicians).[36] However, the authors noted a potential Hawthorne effect as all wounds had been evaluated by an attending physician prior to allocation to one of the medical team members, based on

For peer review only - http://bmjopen.bmj.com/site/about/guidelines.xhtml

their level of training. It was noted that PAs in this study, with nine to 12 years' experience, were classified as experienced (not junior) practitioners.

Trauma and orthopaedics

Four papers reported on PAs working in trauma and orthopaedics. These spanned a 10 year period. Three [37,39,40] focused on an aspect of provision of a hospital trauma service; and one considered planned inpatient care.[38]

Two studies described how PAs were substituting for doctors, for residents [40] or GP surgical assistants [38], whilst the others presented service re-organisations of which PAs were a part, seemingly an addition to the pre-existing medical team [37, 39] The outcomes assessed were numerous - patient satisfaction, perceptions of other clinical staff, costs, time of various aspects of care, length of stay, operative complications and mortality. The strength of evidence for each outcome is now assessed.

One prospective study reported both patient satisfaction and acceptability of PAs to other clinical staff from surveys of these groups. Positive results were presented from the patient satisfaction survey, although the number of respondents was small and no comparator data were collected. The reports of staff were more mixed, with physician team members being positive and nursing staff more equivocal, expressing concern about the overlap of tasks traditionally considered to be the responsibility of nurses.

Operational measures were_addressed in all four of the studies in this specialty, split into a number of outcomes pertaining to time [37-40] and to cost. [37,38]

The evidence of the impact of PAs on access times was equivocal. One study reported how the wait to be seen and the length of treatment by the orthopaedic service in the emergency

For peer review only - http://bmjopen.bmj.com/site/about/guidelines.xhtml

department section of their orthopaedic pathway were significantly shortened when PAs were substituted directly for doctors, although the authors attributed this to a combination of factors, and not just to the PAs, including more registered nurse cover, introduction of a family practice resident and other changing practices.[40] Another found the same when PAs were added to the team as part of larger trauma team re-organisation.[37] Median number of weeks to wait for surgical procedures were also reported to be reduced,[38] attributed by the authors to the use of two operating theatres by the surgeon, made possible by the PA preparing and finishing the case. In terms of time, Althausen et al (2013)[37] reported in detail on operating room times – set up, wound closure to out of theatre, average operating room time – and only noted a minimal (not statistically significant) difference for set up time in a direct comparison study. PAs also released time for supervising physicians and general practitioners (GPs), who had previously acted as surgical assistants [38].

Three high quality studies [37,39,40] reported variably on length of hospital stay, with one showing a significant reduction (three to four hours, a fraction of one day) for all patients when PAs were an addition to either the resident physician team or reorganised trauma panel[39] and two replacement studies finding no difference – when carrying out adjusted analyses of one year against another[40] or when PAs were present or not.[37]

Evidence regarding cost was again mixed. Bohm[38] suggests the actual costs of employment were similar to those of the GPs they replaced in the operating room but argue an opportunity cost for others through released time for supervising physicians. However, a non-replacement model, Althausen[37] reported specific cost savings in the ED and operating room based on time reduction and PA charges (although they noted that only 50% of PA costs were covered through charges).

For peer review only - http://bmjopen.bmj.com/site/about/guidelines.xhtml

As well as these operational measures, these studies also reported health outcomes, and all reported improvement in these.[37,39,40] One considered the rate of complication from procedures involving physician associates [37] and two reported on mortality.[39,40] In terms of operating room complication rates, these did not differ significantly, but postoperative complications were reported to have decreased and antibiotic use and DVT prophylaxis increased (statistically significantly) for cases with a physician associate present (although it is noted that the tables in this paper presented the findings contradictory to the text and abstract).[37] One study assessing mortality in two, year long periods reported that involvement of PAs in the clinical team had no effect on overall mortality rates[40] while another found that mortality decreased by approximately one per cent with the introduction of PAs to a trauma panel and 1.5% to general surgery residents' teams.[39] However, this could not be directly attributable to the addition of the PA because contemporaneous improvements in efficiency of the trauma service occurred.

Internal (acute) medicine

The only study [41] considering PAs in internal (acute) medicine examined resource use. This study measured length of stay, direct costs, and outcomes for patients with diagnoses of cerebrovascular accident, pneumonia, acute myocardial infarction discharged alive, congestive heart failure and gastrointestinal haemorrhage. In this controlled comparative replacement (PAs for interns/residents) study no significant differences in length of stay were found between patients admitted by attending physicians to teams with a physician associate or team with an intern/resident, with length of stay considered to be a proxy for severity of illness. Cost in terms of relative value units (RVUs, based on billing information for physician-ordered items, excluding administrative costs outside of the physician's control)

For peer review only - http://bmjopen.bmj.com/site/about/guidelines.xhtml

was also mostly similar although laboratory RVUs were lower for PAs, that is, they ordered fewer investigations after adjustment for demographics in each diagnostic group. The authors concluded that PAs used resources as effectively as, or more effectively than, residents.

DISCUSSION

Principal findings

This systematic review identified a large number of studies of PAs working in secondary care settings, internationally. However, once studies were excluded that ddi not meet the inclusion criteria, only 11 papers remained. Most of the included studies were from the emergency medicine and trauma and orthopaedics specialties, with one from internal medicine. We found no studies in our other specialties of interest – care of the elderly and mental health – where other larger groupings of physician associates worked in the UK according to national survey[16] at the time of planning this review. Several of the studies were of high quality, providing comparative data, and some contained statistical adjustments to address confounding; however all findings were observational. While we recognise that trials are rarely feasible in this type of workforce intervention, adjustment for confounding by indication is a serious challenge in this setting, especially when using a limited routine data source, and there was evidence of both residual confounding from imperfect measures of severity[42] and bias from adjusting for co-variates that were not confounders.[43] Quality also varied widely. This is noteworthy considering that this was a relatively recent set of papers. In addition, comparison and synthesis has been limited by the mix in the papers of those who measure outcomes where PAs are an addition to a team (presenting difficulties in attributing the outcomes to PAs as opposed to any other increase in team capacity) and those

For peer review only - http://bmjopen.bmj.com/site/about/guidelines.xhtml

where PAs substitute for other physicians where the contribution of PAs is actually being measured. Although every paper reported the contribution of PAs in its speciality/subspecialty as overall positive, it is important that the following summary of the main findings of the review is considered in the context of the issues of method and methodological quality.

Results were spread across a number of outcomes, though those related to operational measures were most prevalent. Outcomes reported when employing PAs in emergency medicine were varied. Operational performance results reported were decreased waiting time and reduced length of stay in the emergency department,[32] and an increase in length of visit for those seen by PAs[31] and reduced charges.[31] Health care outcomes reported were no difference in wound infection rate,[36] and differences which were difficult to interpret, for example an increased prescription rate[33], or increase[35] or decrease in analgesia prescribing.[34] The messages are remarkably similar for trauma and orthopaedics. Operational measures highlighted no difference to [40] or reduced [37,38,39] waiting times in the emergency, operative and post-operative phases of care; released physician time[38] and reduced cost.[37] Here the evidence on health outcomes was mostly positive – increased adherence to treatment processes such as antibiotic administration[37] and reduced post-operative complications[37] and either no difference[40] or a reduction[39] in mortality. High patient satisfaction and staff acceptability were also reported.[38]

The one study in internal (acute) medicine was one of the few using a prospective design, and found few differences in efficiency measures between PAs and residents, although there were lower costs for some conditions.[41]

Summarising across the specialties we have reported three studies where PAs were an addition to the team.[32,37,39] In these more patients are reported to have been treated;

For peer review only - http://bmjopen.bmj.com/site/about/guidelines.xhtml

waiting, ED and operating room times are said to have been shorter and mortality to be lower; assessment of the contribution of PAs as opposed to any increase in team capacity is limited. Six studies which compared outcomes of care by PAs and physicians either when one or the other was providing care or when PAs were substituting overall for physicians [31,34,36,38,40,41] presented mixed results: either no or a very small difference to length of stay, reduced resource used but at equal cost, some time savings to senior physicians, lower analgesia prescribing, no difference in wound infection rate or in acceptability to staff and patients. In the two studies carrying out secondary data analysis we do not know if the PAs were additions or substitutions but both reported higher prescribing by PAs.

Strengths and weaknesses

This review has systematically assessed the body of PA literature most immediately applicable to the current UK secondary care setting. We selected the five specialties in which PAs in the UK were mostly reported to be working[16] and therefore drew together the evidence of most relevance in that context and noted prominent gaps in evidence. However, this excluded evidence from other specialties. We excluded any studies including intensive care data as this overlapped with acute medicine in many abstracts and we could not separately draw this out. We note that this literature appeared to include a greater proportion of studies with stronger study designs, including prospective and randomised designs.

All of the included papers were from North America, with the majority from USA, where health service organisation and the PA role may differ from that in other countries developing the PA role. In the USA PAs can prescribe and are, as a body, more experienced than in countries more recently embracing this role.

For peer review only - http://bmjopen.bmj.com/site/about/guidelines.xhtml

We planned to carry out meta-analysis as appropriate to the literature included. The diversity of intervention as in initiation of PAs or change to PA practice being measured prevented this, as did identifying the effect of PAs when there were other simultaneous changes, even where a body of literature pertaining to a particular outcome measure, such as length of stay, was included. Although narrative review is more limited in its precision, in following a framework for this, we have aimed to provide a clear rationale for the synthesis and conclusions we draw from it.

Meaning of the study

This evidence is heavily weighted towards process times and patient satisfaction, with much less on health outcomes, although outcomes are crucial to assess safety of practice for all clinicians. Similar findings have been reported in a systematic review of new (non-medical) roles in emergency medicine – reductions in waiting times in emergency departments, high level of patient satisfaction, confidence and acceptance of the roles. [44] Evidence also suggests that the perception of waiting times and satisfaction are correlated. [45]

Evidence within the parameters of this review from outside of the USA is very slim, as is evidence from multi-centre studies. The case for PAs in the UK secondary care setting is made on the stability they might offer to medical teams and their broad knowledge in the face of hyper-specialisation.[46] Our recently-acquired knowledge suggests that PAs in England work in teams of multiple medical and other clinical staff grades[47] and that they are seen primarily as a resource where there are significant medical staffing issues.[48] When we place this emerging evidence alongside the exponential growth in training numbers for PAs in England (alongside other UK countries),[49] government support for their professional regulation[50] and increased numbers (in primary care at least)[51] we suggest that this

For peer review only - http://bmjopen.bmj.com/site/about/guidelines.xhtml

professional group is judged by increasing numbers of employers and workforce planners to be of value, alongside other medical associate professions.[52].

The studies included in this review can be seen as complex interventions in complex systems and yet this has not been considered in the conclusions the authors draw. Well-controlled studies are needed to fill in the gaps in our knowledge about the outcomes of PAs' contribution to the secondary care. High quality substitution evidence from the Netherlands,[53,54] published since we conducted this review, suggests that the role differs in offering greater continuity and PA-managed wards are similarly cost effective to resident-managed wards. More such evidence is required as well as further evaluation from a realist perspective – considering context, mechanisms and outcome - if PAs cannot be separated from service; measurement would utilise the principles of realist complex intervention science[55] or process evaluation to “Clearly describe the intervention and clarify causal assumptions (in relation to how it will be implemented, and the mechanisms through which it will produce change, in a specific context).”[56]

For peer review only

For peer review only - http://bmjopen.bmj.com/site/about/guidelines.xhtml

Conclusion

Limited research evidence on physician associates working in emergency medicine, trauma and orthopaedics and internal (acute) medicine exists, with a lack of evidence in other specialties. The studies report mostly positive results but these are difficult to interpret in studies where cause and effect cannot be attributed as the PAs worked as additions as well as substitutes in complex systems where work is organised in teams. Physician associate employment is often part of wider service re-design or staffing recalibration as a result of other changes, for example, availability of medical staff. Rigorous evaluation is required to address the complexity of the PA role, reporting on more than one setting and should include comparators. Clinicians, managers, service commissioners and service users need more evidence about the contribution of physician associates/assistants.

For peer review only - http://bmjopen.bmj.com/site/about/guidelines.xhtml

COMPETING INTERESTS

SdeL is Head of Department of Clinical and Experimental Medicine; one of the Sections in this Department – Medical Education - runs a Physician Associate course.

- JP chairs the UK and Ireland Board for Physician Associate Education and is director of the Physician Associate programme at the University of Birmingham.

FUNDING AND DISCLAIMER

This project was funded by the National Institute for Health Research Health Services and Delivery Research Programme (project number 14/19/26).

The views and opinions expressed herein are those of the authors and do not necessarily reflect those of the HS&DR Programme, NIHR, NHS or the Department of Health.

DATA SHARING STATEMENT

No further data are available

CONTRIBUTOR STATEMENT

MH led the design, execution and writing of this paper, under the direction of the study's PI VMD and supported by discussion with and written feedback from all co-authors'(CW, FP, HG, SdeL, JP, RG, JG, LN) on the design of the review and interpretation of findings. In addition, MH, CW, FP and VMD searched for literature and carried out data extraction and quality assessment. All authors (CW, FP, HG, SdeL, JP, RG, JG, LN, VMD) contributed intellectual content to the paper.

For peer review only - http://bmjopen.bmj.com/site/about/guidelines.xhtml

For peer review only

TABLES AND FIGURES:

Figure 1: PRISMA flow chart

Figure 2: 'Risk of bias' graph: review authors' judgements about each risk of bias item presented as percentages across all included studies

Supplementary file 1: Scoping review (Preliminary Medline search strategy – 24/11/2015) and MeSH (Medical Subject Headings) definition of search terms used

For peer review only

For peer review only - http://bmjopen.bmj.com/site/about/guidelines.xhtml

REFERENCES

1. World Health Organisation Global strategy on human resources for health: Workforce 2030. Draft (2015) Accessed last 12-09-2016 http://www.who.int/hrh/resources/pub_globstrathrh-2030/en/

2. World Health Organisation. Task shifting: rational redistribution of tasks among health workforce teams - global recommendations and guidelines. Geneva: World Health Organisation; 2008.

3. Mittman DE, Cawley JF, Fenn WH. Physician assistants in the United States. *BMJ* 2002;325:485-487.

4. AAPA. 2013 Annual Survey Report. Accessed March 22 2017 at https://www.aapa.org/wp-content/uploads/2016/12/Annual_Server_Data_Tables-S.pdf

5. AAPA. What is a PA? Accessed March 22 2017 at https://www.aapa.org/what-is-a-pa/

6. Hooker RS, Hogan K, Leeker E. The globalization of the physician assistant profession. *J Physician Assist Educ* 2007;18:76–85.

7. Health Education England. HEE Business Plan 2015/16. 201515. Accessed 02-09-2016 https://hee.nhs.uk/sites/default/files/documents/HEE-Business-Plan-2015-16.pdf

8. Healthforce Ontario 2009 Ontario’s Physician Assistant Initiative: An Overview. Accessed on 13-09-2016 at http://www.healthforceontario.ca/en/M4/Ontario%27s_Physician_Assistant_Initiative

For peer review only

For peer review only - http://bmjopen.bmj.com/site/about/guidelines.xhtml

9. Synergia Limited. 2015. Phase II of the Physician Assistant Demonstrations Evaluation Report: Report for Health Workforce New Zealand. Wellington: Ministry of Health. Accessed last 2-9-2016 at http://www.health.govt.nz/publication/phase-ii-physician-assistant-demonstrations-evaluation-report

10. U.S. Department of Health and Human Services Health Resources and Services Administration. National Center for Health Workforce Analysis. The U.S. Health workforce chartbook. 2013 Accessed last on 13-09-2016 at http://bhpr.hrsa.gov/healthworkforce/supplydemand/usworkforce/chartbook/chartbookpart1.pdf

11. Velde FVD, Windt WVD. Alumni van de masteropleiding Physician Assistant. Utrecht: Kiwa Prismant; 2013. Cited in Timmermans MJ, van Vught AJ, Wensing M, Laurant MG. The effectiveness of substitution of hospital ward care from medical doctors to physician assistants: a study protocol. *BMC Health Serv Res* 2014;14:43. doi: 10.1186/1472-6963-14-43.

12. Ross N, Parle J, Begg P, Kuhns D. The case for the physician assistant. *Clin Med* 2012;12:200-6.

13. Hooker RS, Everett CM. The contributions of physician assistants in primary care systems. *Health Soc Care Community* 2012;20–31.

14. Halter M, Drennan V, Chattopadhyay K, et al. The contribution of physician assistants in primary care: a systematic review. *BMC Health Serv Res* 2013;18:223. doi: 10.1186/1472-6963-13-223.

15. Doan Q, Sabhaney V, Kissoon N, et al. A systematic review: the role and impact of the physician assistant in the emergency department. *Emerg Med Australas* 2011;23:7-15.

For peer review only - http://bmjopen.bmj.com/site/about/guidelines.xhtml

16. Ritsema TS. Faculty of Physician Associates Census Results 2016. Accessed 10 September 2017 at http://www.fparcp.co.uk/about-fpa/fpa-census

17. Merkle F, Ritsema TS, Bauer S, Kuilman L. The physician assistant: Shifting the Paradigm of European medical practice? *HSR Proc Intensive Care Cardiovasc Anesth* 2011;3:255-262.

18. Moher D, Liberati A, Tetzlaff J et al. The PRISMA Group. Preferred reporting items for systematic reviews and meta-analyses: the PRISMA statement. *J Clin Epidemiol* 2009;62:1006–12.

19. Halter M, Wheeler C, Drennan V, et al. Evidence of impact of physician associates in secondary care: a systematic mixed studies review. PROSPERO 2016:CRD42016032895. Accessed on 10 September at2017http://www.crd.york.ac.uk/PROSPERO/display_record.asp?ID=CRD42016032895.

20. Greenhalgh T, Peacock R. Effectiveness and efficiency of search methods in systematic reviews of complex evidence: audit of primary sources. *BMJ* 2005; 331:1064–1065

21. Department of Health. The Competence and Curriculum Framework for the Physician Assistant. London: Department of Health 2006.

22. Maxwell R. Dimensions of quality revisited: from thought to action. *Qual Health Care* 1992:1:171-177.

23. Department of Health. High Quality Care For All NHS Next Stage Review Final Report. 2008. https://www.gov.uk/government/uploads/system/uploads/attachment_data/file/228836/7432.pdf Accessed 10 December 2015.

For peer review only - http://bmjopen.bmj.com/site/about/guidelines.xhtml

24. International Monetary Fund. Table B1. Advanced Economies: Unemployment, Employment, and Real GDP per Capita. https://www.imf.org/external/pubs/ft/weo/2015/01/pdf/tblpartb.pdf Accessed on 14 December 2015.

25. Kmet LM, Lee RC, Cook LS. Standard quality assessment criteria for evaluating primary research papers from a variety of fields. *Alberta Herit Found Med Res* 2004;13:1–11. http://files.deslibris.ca/cppc/200/200548.pdf Accessed November 24 2015.

26. Pluye P, Robert E, Cargo M, et al. Proposal: A mixed methods appraisal tool for systematic mixed studies reviews. 2011. Retrieved on 24 November 2015 from http://mixedmethodsappraisaltoolpublic.pbworks.com

27. Centre for Reviews and Dissemination, University of York. Systematic Reviews. CRD's guidance for undertaking reviews in healthcare. 2009.

28. Thomas J, Harden A: Methods for the thematic synthesis of qualitative research in systematic reviews. *BMC Med Res Meth* 2008;8:45.

29. Popay J, Roberts H, Sowden A, et al. Guidance on the conduct of narrative synthesis in systematic reviews. ESRC Research Methods Programme. 2006.

30. Noyes J & Lewin S. Chapter 6: Supplemental Guidance on Selecting a Method of Qualitative Evidence Synthesis, and Integrating Qualitative Evidence with Cochrane Intervention Reviews. In: Noyes J, Booth A, Hannes K, et al (editors), Supplementary Guidance for Inclusion of Qualitative Research in Cochrane Systematic Reviews of Interventions. Version 1 (updated August 2011). Cochrane Collaboration Qualitative Methods Group, 2011. Available from URL http://cqrmg.cochrane.org/supplemental-handbook-guidance Last accessed January 2015.

For peer review only - http://bmjopen.bmj.com/site/about/guidelines.xhtml

31. Arnopolin SL, Smithline HA. Patient care by physician assistants and by physicians in an emergency department. *JAAPA* 2000;13:39-40, 49-50, 53-4 passim.

32. Ducharme J, Alder RJ, Pelletier C, et al. The impact on patient flow after the integration of nurse practitioners and physician assistants in 6 Ontario emergency departments. CJEM. 2009;11:455-461.

33. Hooker RS, Cipher DJ, Cawley JF, et al. Emergency medicine services: Interprofessional care trends. J Interprof Care 2009;22: 167-178.

34. Kozlowski MJ, Wiater JG, Pasqual RG, et al. Painful discrimination: The differential use of analgesia in isolated lower limb injuries. Am J Emerg Med.2002;20:502-5.

35. Ritsema TS, Kelen GD, Pronovost PJ, et al. The National Trend in Quality of Emergency Department Pain Management for Long Bone Fractures. *Acad Emerg Med* 2007;14:163-9.

36. Singer AJ, Hollander JE, Cassara G, et al. Level of training, wound care practices, and infection rates. *Am J Emerg Med* 1995;13:265-8.

37. Althausen PL, Shannon S, Owens B, et al. Impact of hospital-employed physician assistants on a level II community-based orthopaedic trauma system. *J Orthop Trauma.* 2013;27:e87-91. doi: 10.1097/BOT.0b013e3182647f29.

38. Bohm ER, Dunbar M, Pitman D, et al. Experience with physician assistants in a Canadian arthroplasty program. *Can J Sur*. 2010;53:103-8.

39. Mains C Scarborough K, Baror R, et al. Staff commitment to trauma care improves mortality and length of stay at a level i trauma center. *J Trauma* 2009;66:1315-20. doi: 10.1097/TA.0b013e31819d96d8.

40. Oswanski MF, Sharma OP, Raj SS. Comparative review of use of physician assistants in a level I trauma center. *Am Surg* 2004 Mar;70:272-9.

For peer review only - http://bmjopen.bmj.com/site/about/guidelines.xhtml

41. Van Rhee J, Ritchie J, Eward A. Resource use by physician assistant services versus teaching services *JAAPA* 2002;15:33-8,40,42.

42. Greenland S, Robins JM, Pearl J. Confounding and collapsibility in causal inference. *Statistical Science* 14:29-46. http://www.biostat.harvard.edu/robins/publications/Confounding_and_collapsibility.pdf] Accessed March 30th 2017.

43. Greenland S, Pearl J, Robins JM. Causal diagrams for epidemiological research. *Epidemiology* 1999;10:37-48. http://www.biostat.harvard.edu/robins/publications/causaldia.pdf] Accessed March 30th 2017.

44. Hoskins R. Evaluating new roles within emergency care: a literature review. *Int Emerg Nurs* 2011;19):125-40. doi: 10.1016/j.ienj.2010.09.003.

45. Saxon K, London K, Bacharouch A, et al. Patients' Perceptions of Waiting Times and the Effect on Patient Satisfaction in the Emergency Department. *Ann Emer Med* 2013;62,4 Supplement:S82

46. Ross N, Parle J, Begg P et al. The case for the physician assistant. *Clinical Medicine* 2012;12:200–6.

47. Wheeler C, Halter M, Drennan VM, et al. Physician associates working in secondary care teams in England: Interprofessional implications from a national survey. *J Interprof Care* 2017;6:1-3. doi: 10.1080/13561820.2017.1341390.

48. Halter M, Wheeler C, Drennan V, et al. Physician Associates in England's hospitals: a survey of medical directors exploring current usage and supporting/inhibiting factors to their recruitment. *Clin Med* 2017;17:126-131. doi: 10.7861/clinmedicine.17-2-126.

49. Ritsema T. (2016) Faculty of Physician Associates Census Results 2016. Accessed last 1-11-2016 at http://www.fparcp.co.uk/censusresults

For peer review only - http://bmjopen.bmj.com/site/about/guidelines.xhtml

50. Lind S. Physician associate legislation waiting for 'parliamentary slot', says Jeremy Hunt. Pulse, 21 December 2016. http://www.pulsetoday.co.uk/your-practice/regulation/physician-associate-legislation-waiting-for-parliamentary-slot-says-jeremy-hunt/20033474.article Accessed 7th Feb 2017.

51. Department of Health and The Rt Hon Jeremy Hunt MP. New Deal for General Practice, https://www.gov.uk/government/speeches/new-deal-for-general-practice (2015, accessed 8th August 2016).

52. Health Education England. Medical Associate Professionals. https://hee.nhs.uk/printpdf/our-work/developing-our-workforce/medical-associate-professions. Accessed 7 February 2017.

53. Timmermans MJC, van Vught AJAH, Van den Berg M, et al. Physician assistants in medical ward care: a descriptive study of the situation in the Netherlands. *J Eval Clin Pract* 2016;22:395-402.

54. Timmermans MJC, van den Brink GT, van Vught AJAH, et al. The involvement of physician assistants in inpatient care in hospitals in the Netherlands: a cost-effectiveness analysis. *BMJ Open*. 2017;7:e016405. doi: 10.1136/bmjopen-2017-016405.

55. Fletcher A, Jamal F, Moore G, et al. Realist complex intervention science: Applying realist principles across all phases of the Medical Research Council framework for developing and evaluating complex interventions. *Evaluation* 2016;22:286-303

56. Moore GF, Audrey S, Barker M, et al. Process evaluation of complex interventions: Medical Research Council guidance. *BMJ* 2015;350:p.h1258.

For peer review only - http://bmjopen.bmj.com/site/about/guidelines.xhtml

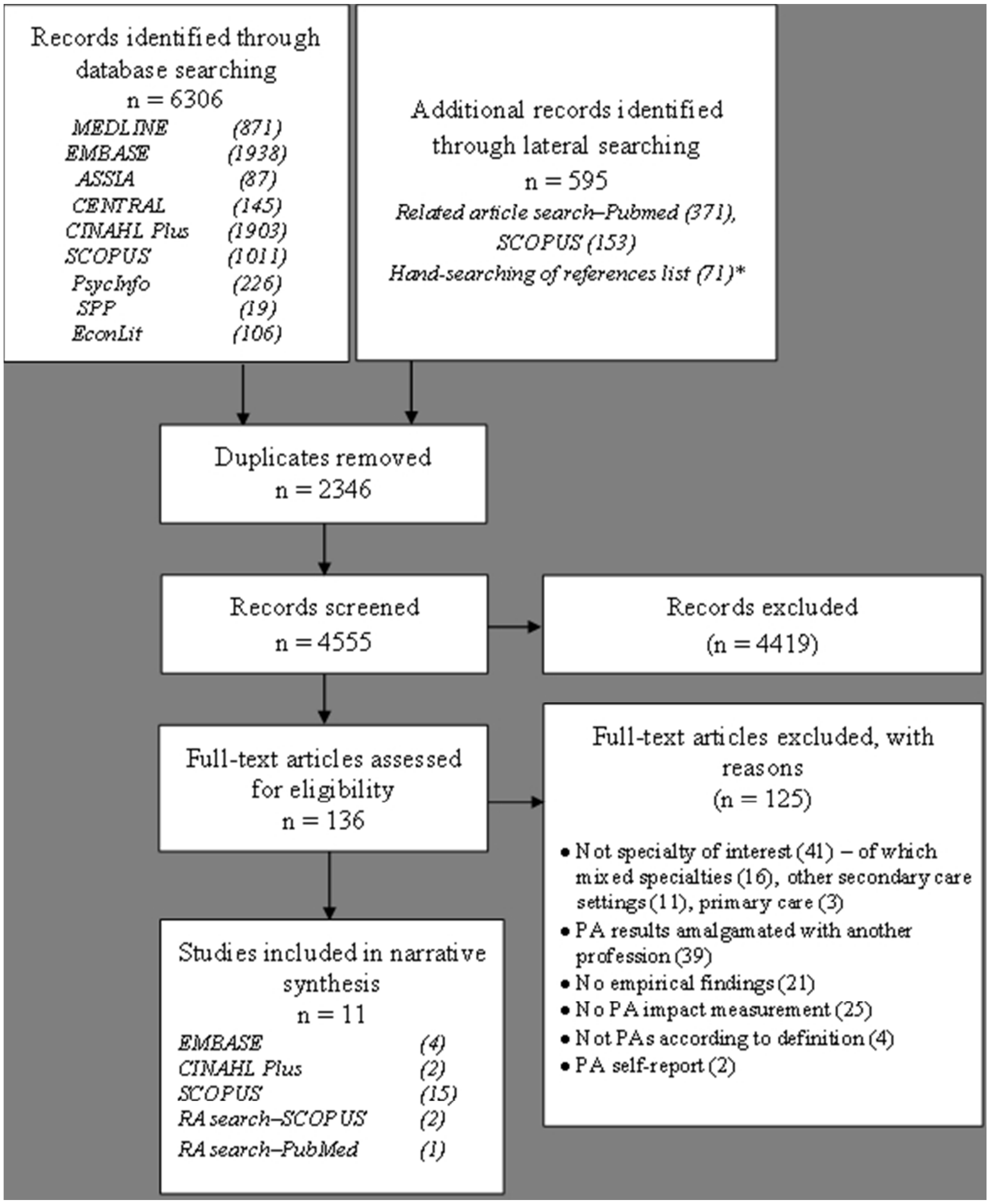

Figure 1: PRISMA flow chart

154x187mm (96 x 96 DPI)

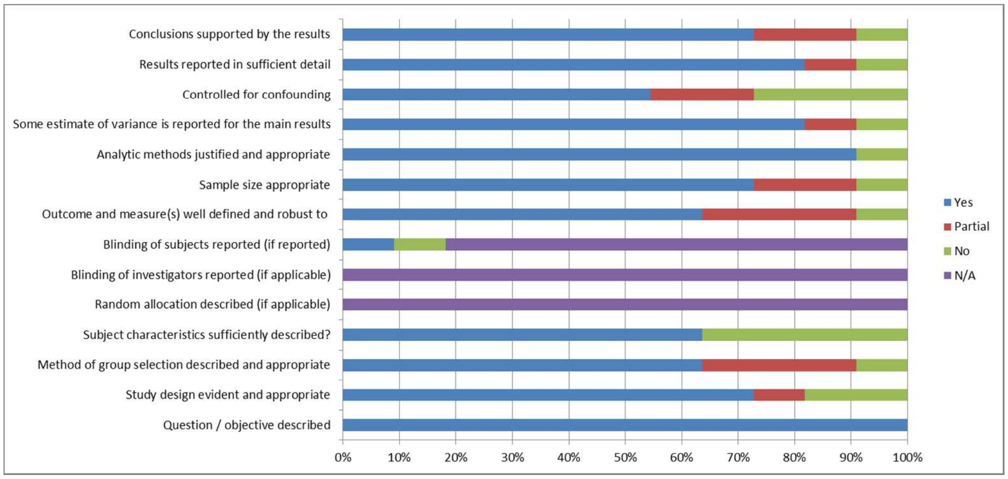

Figure 2: 'Risk of bias' graph: review authors' judgements about each risk of bias item presented as percentages across all included studies

SUPPLEMENTARY FILE 1: Scoping review (Preliminary Medline search strategy – 24/11/2015)

#	Concept		Search Terms	Results
1	**Physician Associates**		exp Physician Assistants/	2410
2			exp Pediatric Assistants/	26
3			Physician Assistant$.tw.	1498
4			Feldsher$.tw.	17
5			Clinical Officer$.tw.	135
6			Paramedical Practitioner&.tw.	0
7			Medical Assistant$.tw.	324
8			Allied Health Personnel.tw.	48
9			physician associate$.tw.	37
10			(mid level adj3 provider$).tw.	124
11			((assistant* or technician* or officer* or associate$) adj2 (physician$ or surgical or clinical$ or practitioner$ or medical$ or provider$)).tw.	24985
12	**Secondary Care**	**Emergency Medicine**	exp Emergency Medicine/ and (speciali?ed or specialty or hospital$ or secondary or care or medicine).tw.	4983
13			((accident and emergency) or A&E department or emergency department or casualty or emergency Medicine).tw.	47842
14			(emergency adj3 (medic* or servic* or ward* or department)).tw.	54262
15			(exp critical care/ or exp intensive care/) and (speciali?ed or specialty or hospital$ or secondary or care or medicine).tw.	23791
16			((intensive adj3 care) and (speciali?ed or specialty or hospital$ or secondary or care or medicine)).tw.	71552
17		**Acute Medicine**	exp Internal Medicine/ and (speciali?ed or specialty or hospital$ or secondary or care or medicine).tw.	16968
18			(internal medicine and (speciali?ed or specialty or hospital$ or secondary or care or medicine)).tw.	10752
19			(Acute Medicine or acute internal medicine or acute medical unit$ or medical assessment unit$ or acute ward$).tw.	690
20		**Trauma or Orthopaedics**	(exp Orthopedics/ or exp Traumatology/) and (speciali?ed or specialty or hospital$ or secondary or care or medicine).tw.	3015
21			((Trauma or Orthop?dic$) adj3 (speciali?ed or specialty or hospital$ or secondary or care or medicine)).tw.	7280
22			(Orthop?dic surgery or trauma surgery).tw.	4466
23			((bone$ or joint$ or ligament$ or tendon$ or muscle$ or nerve$) adj3 (operation$ or surgery or replacement$)).tw.	13668
24		**Care of the Elderly**	(exp geriatrics/ or Aging/ or exp Aged/ or older people.mp. or exp Frail Elderly/) and (speciali?ed or specialty or hospital$ or secondary or care).tw.	361294
25			((Older adult or Aged or elderly or geriatric* or older people* or ag?ng) adj3 (speciali?ed or specialty or hospital$ or secondary or care or medicine)).tw.	15561

For peer review only

26			or/12-25	508965
27			exp Primary Health Care/ or exp preventive medicine/ or exp physicians, Primary Care/	75166
28			(primary care or primary healthcare or primary health care or primary health service$).tw.	68593
29			27 or 28	111510
30		**Primary care**	exp Family Practice/ or exp Physicians, Family/ or exp General Practitioners/ or exp General Practice/	47498
31			(family practice$ or family practitioner$ or family physician$ family medicine$ or General practice$ or General practitioner$ or GPs).tw.	47129
32			30 or 31	72038
33			29 not 32	91680
34		**Outpatient and inpatient care**	(exp Outpatients/ or Outpatient Clinics, Hospital/ or ambulatory care/) and (speciali?ed or specialty or hospital$ or secondary or care or medicine).tw.	18427
35			(exp Inpatients/ or Hospitalization/) and (speciali?ed or specialty or hospital$ or secondary or care or medicine).tw.	49797
36			(ambulatory care or ambulatory emergency care).tw.	3948
37			((outpatient$ or out-patient$) adj3 (speciali?ed or specialty or hospital$ or secondary or care or medicine)).tw.	11455
38			((inpatient$ or in-patient$) adj3 (speciali?ed or specialty or hospital$ or secondary or care or medicine)).tw.	24157
39			Treatment Outcome/ or "Outcome and Process Assessment (Health Care)"/ or "Outcome Assessment (Health Care)"/ or Medical Audit/ or Program Evaluation/	769470
40			exp Patient Readmission/ or exp Length of Stay/ or exp Clinical Audit/ or exp Medical Audit/	68267
41			Health Planning/ and (organi?ation* or system* or hospital* or Physician* or workforce or staff or professional*).tw.	2686
42			Efficiency, Organizational/ and (organi?ation* or system* or hospital* or Physician* or workforce or staff or professional*).tw.	8952
43	**Impact**		Resource Allocation/ and (organi?ation* or system* or hospital* or Physician* or workforce or staff or professional*).tw.	1377
44			Health Personnel/ and (organi?ation* or system* or hospital* or Physician* or workforce or staff or professional*).tw.	11958
45			Health Manpower/ and (organi?ation* or system* or hospital* or Physician* or workforce or staff or professional*).tw.	2123
46			Medical Staff/ and (organi?ation* or system* or hospital* or Physician* or workforce or staff or professional*).tw.	899
47			Delivery of Health Care/ and (productivity or efficiency or performance or guideline* or quality).tw.	8411
48			((equity or difference$ disparit$ or inequalit$ or inequit$) adj5 (experience$ or perception$ or view$ or rates or rating or review or audit or impact or influence or effect or outcome or performance or quality)).tw.	2048
49			((Acceptability or compassion or dignity or satisfaction or dissatisfaction) adj5 (experience$ or perception$ or view$ or rates or rating or review or audit or impact or influence or effect or outcome or performance or quality)).tw.	16604

For peer review only

50		((Efficiency or productivity or economic$ or benefit) adj5 (experience$ or perception$ or view$ or rates or rating or review or audit or impact or influence or effect or outcome or performance or quality)).tw.	34565
51		((Effectiveness or efficacy or effectivity or capability) adj5 (experience$ or perception$ or view$ or rates or rating or review or audit or impact or influence or effect or outcome or performance or quality)).tw.	35758
52		((Effectiveness or efficacy or effectivity or capability) adj5 (experience$ or perception$ or view$ or rates or rating or review or audit or impact or influence or effect or outcome or performance or quality)).tw.	35758
53		((Access$ or responsiveness or timely or timeliness) adj5 (experience$ or perception$ or view$ or rates or rating or review or audit or impact or influence or effect or outcome or performance or quality)).tw.	16251
54		((Appropriate$ or relevance or relevant) adj5 (experience$ or perception$ or view$ or rates or rating or review or audit or impact or influence or effect or outcome or performance or quality)).tw.	32405
55		((Cost$ or afford$ value for money or financ$) adj5 (experience$ or perception$ or view$ or rates or rating or review or audit or impact or influence or effect or outcome or performance or quality)).tw.	33373
56	**Impact in Secondary Care of Physician Associates**	or/1-11	26515
57		26 or 33 or 34 or 35 or 36 or 37 or 38	621770
58		or/39-55	959419
59		56 and 57 and 58	1575
60		limit 59 to (english language and last 20 years)	1513

For peer review only

SUPPLEMENTARY FILE 1 continued: MeSH --Medical Subject Headings definition of search terms used

(alphabetical [US spellings])

http://www.ncbi.nlm.nih.gov/mesh

Aged: A person 65 through 79 years of age. For a person older than 79 years, AGED, 80 AND OVER is available. Year introduced: 1966. By exploding this term, we do include MeSH terms found below it in the MeSH hierarchy as follows: Aged, 80 and over; Frail Elderly.

Aging: The gradual irreversible changes in structure and function of an organism that occur as a result of the passage of time. By exploding this term, we do include MeSH terms found below it in the MeSH hierarchy as follows: Longevity.

Ambulatory care: Health care services provided to patients on an ambulatory basis, rather than by admission to a hospital or other health care facility. The services may be a part of a hospital, augmenting its inpatient services, or may be provided at a free-standing facility. Year introduced: 1968(1966)

Behavioral Disciplines and Activities: The specialties in psychiatry and psychology, their diagnostic techniques and tests, their therapeutic methods, and psychiatric and psychological services. Year introduced: 1998

Clinical Audit: A detailed review and evaluation of selected clinical records by qualified professional personnel to improve the quality of patient care and outcomes. The clinical audit was formally introduced in 1993 into the United Kingdom's National Health Service. Year introduced: 2008

Critical Care: Health care provided to a critically ill patient during a medical emergency or crisis. Year introduced: 1975

Emergency medicine: The branch of medicine concerned with the evaluation and initial treatment of urgent and emergent medical problems, such as those caused by accidents, trauma, sudden illness, poisoning, or disasters. Emergency medical care can be provided at the hospital or at sites outside the medical facility.

Family Practice: A medical specialty concerned with the provision of continuing, comprehensive primary health care for the entire family.

For peer review only

PA-SCer systematic review narrative synthesis: first draft T&O and internal med 160806

Frail Elderly: Older adults or aged individuals who are lacking in general strength and are unusually susceptible to disease or to other infirmity. Year introduced: 1991

General Practice: Patient-based medical care provided across age and gender or specialty boundaries. Year introduced: 2011

General Practitioners: Physicians whose practice is not restricted to a specific field of medicine

Geriatrics: The branch of medicine concerned with the physiological and pathological aspects of the aged, including the clinical problems of senescence and senility.

Hospitalization: The confinement of a patient in a hospital.

Inpatients: Persons admitted to health facilities which provide board and room, for the purpose of observation, care, diagnosis or treatment.

Intensive care: Advanced and highly specialized care provided to medical or surgical patients whose conditions are life-threatening and require comprehensive care and constant monitoring. It is usually administered in specially equipped units of a health care facility. Year introduced: 1992

Internal Medicine: A medical specialty concerned with the diagnosis and treatment of diseases of the internal organ systems of adults. By exploding this term, we do include MeSH terms found below it in the MeSH hierarchy as follows: Cardiology; Cardiac electrophysiology; Endocrinology; Gastroenterology; Hematology; Transfusion Medicine; Infectious Disease Medicine; Medical Oncology Radiation; Oncology; Nephrology; Pulmonary Medicine; Rheumatology; Sleep Medicine Specialty.

Medical Audit: A detailed review and evaluation of selected clinical records by qualified professional personnel for evaluating quality of medical care. Year introduced: 1968

Mental Disorders: Psychiatric illness or diseases manifested by breakdowns in the adaptational process expressed primarily as abnormalities of thought, feeling, and behavior producing either distress or impairment of function. Year introduced: use pre-explosion 1974-1997

Mental Health Services: Organized services to provide **mental health** care. Year introduced: 1967
Mental Health: The state wherein the person is well adjusted. Year introduced: 1967

Orthopedics: A surgical specialty which utilizes medical, surgical, and physical methods to treat and correct deformities, diseases, and injuries to the skeletal system, its articulations, and associated structures.

For peer review only

Outcome and Process Assessment (Health Care): Evaluation procedures that focus on both the outcome or status (OUTCOMES ASSESSMENT) of the patient at the end of an episode of care - presence of symptoms, level of activity, and mortality; and the process (ASSESSMENT, PROCESS) - what is done for the patient diagnostically and therapeutically. Year introduced: 1979. By exploding this term, we do include MeSH terms found below it in the MeSH hierarchy as follows: Outcome Assessment (Health Care); Patient Outcome Assessment; Treatment Outcome; Process Assessment (Health Care)

Outcome Assessment (Health Care): Research aimed at assessing the quality and effectiveness of health care as measured by the attainment of a specified end result or outcome. Measures include parameters such as improved health, lowered morbidity or mortality, and improvement of abnormal states (such as elevated blood pressure). Year introduced: 1992

Outpatient Clinics, Hospital: Organized services in a hospital which provide medical care on an outpatient basis. Year introduced: 1978

Outpatients: Persons who receive ambulatory care at an outpatient department or clinic without room and board being provided. Year introduced: 1991(1980)

Pediatric Assistants: Persons academically trained to provide medical care, under the supervision of a physician, to infants and children. Year introduced: 1991(1975)

Physician Assistants: Health professionals who practice medicine as members of a team with their supervising physicians. They deliver a broad range of medical and surgical services to diverse populations in rural and urban settings. Duties may include physical exams, diagnosis and treatment of disease, interpretation of tests, assist in surgery, and prescribe medications. (from http://www.aapa.orglabout-pas accessed 2114/2011) Year introduced: 1995

Physicians, Family: Those physicians who have completed the education requirements specified by the American Academy of Family Physicians. Year introduced: 1974(1972)

Physicians, Primary Care: Providers of initial care for patients. These PHYSICIANS refer patients when appropriate for secondary or specialist care. Year introduced: 2011

Preventive medicine: A medical specialty primarily concerned with prevention of disease (PRIMARY PREVENTION) and the promotion and preservation of health in the individual. By exploding this term, we do include MeSH terms found below it in the MeSH hierarchy as follows: Environmental Medicine; Occupational Medicine; Preventive Psychiatry.

For peer review only

Primary Health Care: Care which provides integrated, accessible health care services by clinicians who are accountable for addressing a large majority of personal health care needs, developing a sustained partnership with patients, and practicing in the context of family and community. (JAMA 1995;273(3):192) Year introduced: 1974(1972).

Program Evaluation: Studies designed to assess the efficacy of programs. They may include the evaluation of cost-effectiveness, the extent to which objectives are met, or impact. Year introduced: 1989. By exploding this term, we do include MeSH terms found below it in the MeSH hierarchy as follows: benchmarking.

Psychiatry: The medical science that deals with the origin, diagnosis, prevention, and treatment of mental disorders.

Traumatology: The medical specialty which deals with wounds and injuries as well as resulting disability and disorders from physical traumas.

Treatment Outcome: Evaluation undertaken to assess the results or consequences of management and procedures used in combating disease in order to determine the efficacy, effectiveness, safety, and practicability of these interventions in individual cases or series. Year introduced: 1992

For peer review only

SUPPLEMENTARY FILE 2: DEFINITIONS used in this review

As this review question contained broad terms, these were defined at the outset, as follows:

- Physician Associates: trained in a medical model to work in all settings and undertake physical examinations, investigations, diagnosis, treatment, and prescribe within their scope of practice as agreed with their supervising doctor.[1,2] Physician Associates are sometimes described within the term 'mid-level providers' in developed economies: '…..the term mid-level practitioner means an individual practitioner, other than a physician, dentist, veterinarian, or podiatrist, who is licensed, registered, or otherwise permitted by the United States or the jurisdiction in which he/she practices, to dispense a controlled substance in the course of professional practice. Examples of mid-level practitioners include, but are not limited to, health-care providers such as nurse practitioners, nurse midwives, nurse anaesthetists, clinical nurse specialists and physician assistants who are authorized to dispense controlled substances by the state in which they practice.' [3] While this term is contested as an appropriate umbrella term due to its hierarchical connotations [4,5] and international variation in usage,[6] it appears in the literature regarding Physician Associates.
- Impact: using the broad headings of the components of quality as suggested by Maxwell (1992),[7] augmenting that of Donabedian,[8] that is, effectiveness, efficiency, acceptability, access, equity and relevance; further consolidated in the aspects of quality set out in the NHS Next stage Review (2008)[9]: patient safety, patient experience and effectiveness of care.
- Specialties most frequently employing PAs in England:
  - acute medicine

    'Acute medicine is the part of general (internal) medicine concerned with the immediate and early specialist management of adult patients who present to, or from within, hospitals as urgencies or emergencies'.[10]
  - care of the elderly

    '…geriatric medicine is mainly concerned with people over the age of 75, although many 'geriatric' patients are much older. However, geriatric medicine in the UK is broadly from the age of 65 onwards. Frail older people are those with multiple diseases, that often includes dementia, with reduced functional reserve who tend to present to hospital with 'geriatric syndromes' such as falls, confusion and immobility.'[11]
  - emergency medicine

    'Emergency medicine is a field of practice based on the knowledge and skills required for the prevention, diagnosis and management of acute and urgent aspects of illness and injury affecting patients of all age groups with a full spectrum of episodic undifferentiated physical and behavioural disorders; it further encompasses an understanding of the development of

For peer review only

prehospital and in hospital emergency medical systems and the skills necessary for this development.' [12]

– mental health /psychiatry

'Mental health problems can take many forms including depression, schizophrenia, eating disorders, anxieties, phobias, drug and alcohol abuse, post-traumatic stress disorder, and dementia.'[13] Psychiatry includes the sub specialties of child and adolescent, forensic, general adult, old age, psychotherapy and psychiatry of learning disabilities. [14]

- trauma and orthopaedics

Trauma and orthopaedics is an area of surgery concerned with injuries and conditions that affect the musculoskeletal system (the bones, joints, ligaments, tendons, muscles and nerves).[15]

References

1. Department of Health. The Competence and Curriculum Framework for the Physician Assistant. London: Department of Health 2006.
2. Physician Assistant Managed Voluntary Register The Revised Competence and Curriculum Framework for the Physician Assistant. 2012 Accessed at http://www.ukapa.co.uk/files/CCF-27-03-12-for-PAMVR.pdf.
3. United States Department of Justice. (n.d.). Mid-level practitioners authorization by state. Retrieved from http://www.deadiversion.usdoj.gov/drugreg/practioners/index.html
4. Bishop CS. Advanced Practitioners Are Not Mid-Level Providers. Vol 3 No 5 Sep/Oct 2012 287-8. J Adv Pract Oncol AdvancedPractitioner.com http://www.advancedpractitioner.com/media/152572/287.pdf
5. Pappas MD. Stop calling nurse practitioners mid-level providers. 2014 Kevin MD.com. http://www.kevinmd.com/blog/2014/07/stop-calling-nurse-practitioners-mid-level-providers.html. Accessed 24 November 2015.
6. Brown A, Cornetto G, Cumbi A, de Pinho H, Kamwendo F, Lehmann U et al. Mid-level health providers: a promising resource. Rev Peru Med Exp Salud Publica. 2011; 28(2): 308-15.
7. Maxwell R. Dimensions of quality revisited: from thought to action. Quality in Health Care 1992:1:171-177.
8. Donabedian A. The quality of care. How can it be assessed? JAMA 1988;260:1743–8. http://dx.doi.org/10.1001/jama.1988.03410120089033

For peer review only

9. Department of Health. High Quality Care For All NHS Next Stage Review Final Report. 2008. https://www.gov.uk/government/uploads/system/uploads/attachment_data/file/228836/7432.pdf Accessed 10 December 2015.
10. Royal College of Physicians. *Acute medical care; the right person, in the right setting—first time.* Report of a working party. London: RCP, 2007.
11. Conroy S, Cooper N. Executive summary. British Geriatrics Society, 2012. http://www.bgs.org.uk/index.php/topresources/publicationfind/goodpractice/44-gpgacutecare%20 Accessed 24 Nov 2015.
12. International Federation for Emergency Medicine. About IFEM IFEM Definition of Emergency Medicine. 2008. http://www.ifem.cc/About_IFEM.aspx Accessed Nov 24 2015.
13. Royal College of Psychiatrists. Glossary of Terms Professionals involved in the care of people with mental health problems. 2015. http://www.rcpsych.ac.uk/healthadvice/moreinformation/definitions/professionalsinpsychiatry.aspx. Accessed 10 December 2015.
14. Royal College of Psychiatrists. A Career in Psychiatry. 2015. http://www.rcpsych.ac.uk/discoverpsychiatry/acareerinpsychiatry.aspx Accessed 10 December 2015.
15. NHS Choices. Trauma and Orthopaedics (Orthopeadic Surgery) 2015. http://www.nhs.uk/Conditions/orthopaedics/Pages/Introduction.aspx

For peer review only

PRISMA 2009 Checklist

Section/topic	#	Checklist item	Reported on page #
**TITLE**			
Title	1	Identify the report as a systematic review, meta-analysis, or both.	1
**ABSTRACT**			
Structured summary	2	Provide a structured summary including, as applicable: background; objectives; data sources; study eligibility criteria, participants, and interventions; study appraisal and synthesis methods; results; limitations; conclusions and implications of key findings; systematic review registration number.	4
**INTRODUCTION**			
Rationale	3	Describe the rationale for the review in the context of what is already known.	6-7
Objectives	4	Provide an explicit statement of questions being addressed with reference to participants, interventions, comparisons, outcomes, and study design (PICOS).	9
**METHODS**			
Protocol and registration	5	Indicate if a review protocol exists, if and where it can be accessed (e.g., Web address), and, if available, provide registration information including registration number.	8
Eligibility criteria	6	Specify study characteristics (e.g., PICOS, length of follow-up) and report characteristics (e.g., years considered, language, publication status) used as criteria for eligibility, giving rationale.	8
Information sources	7	Describe all information sources (e.g., databases with dates of coverage, contact with study authors to identify additional studies) in the search and date last searched.	8
Search	8	Present full electronic search strategy for at least one database, including any limits used, such that it could be repeated.	Supplementary file
Study selection	9	State the process for selecting studies (i.e., screening, eligibility, included in systematic review, and, if applicable, included in the meta-analysis).	8-9
Data collection process	10	Describe method of data extraction from reports (e.g., piloted forms, independently, in duplicate) and any processes for obtaining and confirming data from investigators.	10
Data items	11	List and define all variables for which data were sought (e.g., PICOS, funding sources) and any assumptions and simplifications made.	10
Risk of bias in individual studies	12	Describe methods used for assessing risk of bias of individual studies (including specification of whether this was done at the study or outcome level), and how this information is to be used in any data synthesis.	10
Summary measures	13	State the principal summary measures (e.g., risk ratio, difference in means).	10-11
Synthesis of results	14	Describe the methods of handling data and combining results of studies, if done, including measures of consistency (e.g., I^2) for each meta-analysis.	10-11

For peer review only - http://bmjopen.bmj.com/site/about/guidelines.xhtml

PRISMA 2009 Checklist

Section/topic	#	Checklist item	Reported on page #
Risk of bias across studies	15	Specify any assessment of risk of bias that may affect the cumulative evidence (e.g., publication bias, selective reporting within studies).	
Additional analyses	16	Describe methods of additional analyses (e.g., sensitivity or subgroup analyses, meta-regression), if done, indicating which were pre-specified.	
**RESULTS**			
Study selection	17	Give numbers of studies screened, assessed for eligibility, and included in the review, with reasons for exclusions at each stage, ideally with a flow diagram.	11+figure1
Study characteristics	18	For each study, present characteristics for which data were extracted (e.g., study size, PICOS, follow-up period) and provide the citations.	Table 1
Risk of bias within studies	19	Present data on risk of bias of each study and, if available, any outcome level assessment (see item 12).	Table 2 + Figure2
Results of individual studies	20	For all outcomes considered (benefits or harms), present, for each study: (a) simple summary data for each intervention group (b) effect estimates and confidence intervals, ideally with a forest plot.	Table 1 + 2 + pages 24-30
Synthesis of results	21	Present results of each meta-analysis done, including confidence intervals and measures of consistency.	n/a
Risk of bias across studies	22	Present results of any assessment of risk of bias across studies (see Item 15).	24
Additional analysis	23	Give results of additional analyses, if done (e.g., sensitivity or subgroup analyses, meta-regression [see Item 16]).	n/a
**DISCUSSION**			
Summary of evidence	24	Summarize the main findings including the strength of evidence for each main outcome; consider their relevance to key groups (e.g., healthcare providers, users, and policy makers).	30-31
Limitations	25	Discuss limitations at study and outcome level (e.g., risk of bias), and at review-level (e.g., incomplete retrieval of identified research, reporting bias).	32-33
Conclusions	26	Provide a general interpretation of the results in the context of other evidence, and implications for future research.	33-34
**FUNDING**			
Funding	27	Describe sources of funding for the systematic review and other support (e.g., supply of data); role of funders for the systematic review.	36

From: Moher D, Liberati A, Tetzlaff J, Altman DG, The PRISMA Group (2009). Preferred Reporting Items for Systematic Reviews and Meta-Analyses: The PRISMA Statement. PLoS Med 6(7): e1000097. doi:10.1371/journal.pmed1000097

For peer review only - http://bmjopen.bmj.com/site/about/guidelines.xhtml

PRISMA 2009 Checklist

For more information, visit: www.prisma-statement.org.

For peer review only

BMJ Open

The contribution of physician assistants/associates to secondary care: a systematic review

Journal:	*BMJ Open*
Manuscript ID	bmjopen-2017-019573.R1
Article Type:	Research
Date Submitted by the Author:	08-Feb-2018
Complete List of Authors:	Halter, Mary; St Georges University Hospital, Faculty of Health and Social Care Sciences<br>Wheeler, Carly; Kingston University Faculty of Health Social Care and Education, Centre for Health and Social Care Research<br>Pelone, Ferruccio; Royal College of Obstetricians and Gynaecologists, National Guidelines Alliance<br>Gage, Heather; University of Surrey, School of Economics<br>de Lusignan, Simon; University of Surrey, Department of Health Care Management and Policy; St. Georges University of London, Division of Population Health Sciences and Education<br>Parle, Jim; University of Birmingham, Institute of Clinical Sciences<br>Grant, Robert; Kingston University Faculty of Health Social Care and Education, Centre for Health and Social Care Research<br>Gabe, Jonathan; Royal Holloway, University of London, Criminology and Sociology<br>Nice, Laura; University of Birmingham, Institute of Clinical Sciences<br>Drennan, Vari; Kingston University Faculty of Health Social Care and Education, Centre for Health and Social Care Research
**Primary Subject Heading**:	Health services research
Secondary Subject Heading:	Emergency medicine, Geriatric medicine, Mental health, Surgery
Keywords:	GENERAL MEDICINE (see Internal Medicine), Quality in health care < HEALTH SERVICES ADMINISTRATION & MANAGEMENT, ORTHOPAEDIC & TRAUMA SURGERY, Physician Assistant

SCHOLARONE™
Manuscripts

The contribution of physician assistants/associates to secondary care: a systematic review

Authors

Corresponding author

Halter M, PhD

Faculty of Health, Social Care and Education

Kingston University and St George's, University of London

Cranmer Terrace

London SW17 0RE

00 44 (0)20 8725 0337

m.halter@sgul.kingston.ac.uk

ORCID: 0000-0001-6636-0621

Wheeler C, PhD

Faculty of Health, Social Care and Education, Kingston University and St George's, University of London, London UK

For peer review only - http://bmjopen.bmj.com/site/about/guidelines.xhtml

Pelone F, PhD

National Guideline Alliance, Royal College of Obstetricians and Gynaecologists, London UK

Gage H, PhD

School of Economics, University of Surrey, Guildford, UK

de Lusignan S, MD, FRCGP

Department of Clinical and Experimental Medicine, University of Surrey, Guildford, UK

Parle J, MD, FRCGP

Institute of Clinical Sciences, University of Birmingham, Birmingham, UK

Grant R, MSc

Faculty of Health, Social Care and Education, Kingston University and St George's, University of London, London UK

Gabe J, PhD

For peer review only - http://bmjopen.bmj.com/site/about/guidelines.xhtml

Centre for Criminology & Sociology, School of Law, Royal Holloway, University of London, Egham, UK

Nice L, PhD

Institute of Clinical Sciences, University of Birmingham, Birmingham, UK

Drennan VM, PhD

Faculty of Health, Social Care and Education, Kingston University and St George’s, University of London, London UK

For peer review only

Word count: 5832 excluding tables

For peer review only - http://bmjopen.bmj.com/site/about/guidelines.xhtml

For peer review only

ABSTRACT

Objective: to appraise and synthesise research on the impact of physician assistants/associates in secondary care, specifically acute internal medicine, care of the elderly, emergency medicine, trauma and orthopaedics, and mental health.

Design Systematic review

Setting: Electronic databases (Medline, Embase, ASSIA, CINAHL, SCOPUS, PsycINFO, Social Policy and Practice, EconLit and Cochrane database), reference lists and related articles.

Included articles: Peer reviewed articles of any study design, published in English, 1995 to 2017.

Interventions: Blinded parallel processes were used to screen abstracts and full text, data extractions and quality assessments against published guidelines. A narrative synthesis was undertaken.

Outcome measures: Impact on: patients' experience and outcomes, service organisation, working practices, other professional groups and costs.

Results: 5472 references were identified and 161 read in full; 16 were included - emergency medicine (seven), trauma and orthopaedics (six), acute internal medicine (two), mental health (one) and care of the elderly (none). All studies were observational, with variable methodological quality.

In emergency medicine and in trauma and orthopaedics, when PAs are added to teams, reduced waiting and process times, lower charges, equivalent readmission rate and good acceptability to staff and patients are reported. Analgesia prescribing, operative complications

For peer review only - http://bmjopen.bmj.com/site/about/guidelines.xhtml

and mortality outcomes were variable. In internal medicine outcomes of care provided by PAs and doctors were equivalent.

Conclusions: PAs have been deployed to increase the capacity of a team, enabling gains in waiting time, throughput, continuity and medical cover. When PAs were compared to medical staff, reassuringly there was little or no negative effect on health outcomes or cost. The difficulty of attributing cause and effect in complex systems where work is organised in teams is highlighted. Further rigorous evaluation is required to address the complexity of the PA role, reporting on more than one setting, and including comparison between PAs and roles for which they are substituting.

ARTICLE SUMMARY

STRENGTHS AND LIMITATIONS OF THIS STUDY

- This study's strengths lie in systematically analysing the empirical evidence for the contribution of physician associates to secondary care, following international guidelines.
- Focusing on specialties in which physician associates are increasingly deployed in the UK, while aiming for international applicability. This methodological approach carries limitations in excluding closely related and sometimes high quality studies that did not meet our strict inclusion criteria, but that are relevant to understanding the impact of PAs in secondary care settings.
- The review was strengthened by using established guidelines to carry out quality assessment of the included studies. Although our approach can be considered

For peer review only

For peer review only - http://bmjopen.bmj.com/site/about/guidelines.xhtml

reductionist, it provides decision makers with consistent information about the quality of the evidence against which to weight the value of individual findings.

For peer review only

For peer review only - http://bmjopen.bmj.com/site/about/guidelines.xhtml

THE CONTRIBUTION OF PHYSICIAN ASSISTANTS/ASSOCIATES TO SECONDARY CARE: A SYSTEMATIC REVIEW

Introduction

Health care systems internationally face substantial medical workforce challenges. [1] An approach used in many countries has been to develop advanced clinical practitioner roles (also sometimes known as mid-level non-physician clinicians), who undertake some of the activities of doctors. [2] One of these roles is the physician assistant/associate (PA). The PA role was first developed, by physicians, in the 1960s in the United States (US) in response to medical shortages in certain specialties and regions.[3] As of the end of 2016, there were 115,547 nationally certified and state-licensed PAs in the US,[4] following 44% growth since 2010. In the US PAs practice as medical professionals in healthcare teams with physicians and other providers in all 50 states.[5] Over the last two decades other countries have been introducing PAs into their health workforce, including Australia, Canada, Germany, Ghana, India, Kenya, the Netherlands, Saudi Arabia, South Africa, Taiwan, and the UK,[6] where they are known as physicians associates. Some countries, including the UK, have national or federal policy commitments to develop PA education programmes and significantly increase their availability,[7,8] while others are determining the value of such roles through demonstration projects.[9] The role has received increasing attention as a potential growth area from the UK government, particularly in primary care[10] where there is evidence that physician associates can be complementary to general practitioner and nursing roles, albeit with limitations due to not currently having prescribing rights.[11] However, in the USA only 21% of physician assistants work in family medicine/general practice;[4] similarly in the UK and the Netherlands they report working in a range of secondary care specialties.[12,13]

For peer review only - http://bmjopen.bmj.com/site/about/guidelines.xhtml

Like many aspects of workforce innovation and change, there is very limited published evidence as to the contribution and impact PAs have within this setting. Existing systematic reviews of the contribution PAs make to health care have considered evidence from primary and secondary care together [14] just primary care, [15] rural healthcare and emergency department [16] or considered PAs and nurse practitioners together in surgical services.[17]. Given the recent trends to utilise PAs internationally in secondary care, our purpose in conducting this new review was to systematically summarise the current evidence in secondary care.

The objective of the review was to appraise and synthesise the published literature on the impact of physician associates on patient experience and outcomes, service organisation, working practices, other professional groups and cost. The review was bounded by consideration of the secondary care specialties in which PAs were most frequently reported to be employed in the UK. Using the annual UK Association of Physician Associates Census (conducted in 2016 with 150 PA respondents),[18] four specialties with relatively larger numbers of PAs replying to the survey were clearly identifiable: acute internal medicine (n=23), emergency medicine (n=23), care of the elderly (n=12) and trauma and orthopaedics (n=10). While three other specialties (cardiology, neurology and general surgery) reported five PAs in each, we selected mental health as our fifth specialty to explore, with four PAs reported,[18] to provide a contrast to the focus on physical health in the other four specialties selected. The concentration of PAs in these clinical areas is consistent with evidence from other European countries developing a PA workforce.[19] The review is intended to inform clinicians and managers considering innovation and change in their secondary care workforce.

For peer review only - http://bmjopen.bmj.com/site/about/guidelines.xhtml

METHODS

Search strategy

This systematic review was designed and reported to meet international guidelines: the Preferred Reporting Items for Systematic Reviews and Meta-Analyses (PRISMA).[20] Full details of the overall search strategy can be found in the research protocol, registered with the International Prospective Register of Systematic Reviews (PROSPERO), CRD42016032895.[21]

Studies addressing the research question were identified by systematic searching for keywords in the following electronic databases: Medline (Ovid), Embase (Ovid), Applied Social Sciences Index and Abstracts (ASSIA), Cumulative Index to Nursing and Allied Health Literature (CINAHL) Plus (EBSCO), SCOPUS –V.4 (Elsevier), PsycINFO, Social Policy and Practice (Ovid), EconLit (EBSCO), and Cochrane Central Register of Controlled Trials (CENTRAL) from the beginning of January 1995 to the beginning of January 2018. The search strategy was performed on the 14th December 2015 and updated on 5th January 2018.. No language or publication status restrictions were imposed at the electronic search strategy stage. We present the Medline search strategy, and the definitions of the MeSH terms employed, in Supplementary file 1.

In addition, we used 'lateral searching' techniques[22] including checking reference lists of systematic reviews identified at the abstract screening stage and papers selected for inclusion after full text reading; using the 'Cited by' option on Scopus, and the 'Related articles' option on PubMed, and tracking citations.

Inclusion criteria and study selection

For peer review only - http://bmjopen.bmj.com/site/about/guidelines.xhtml

Relevant studies were selected according to eligibility criteria using a two-step screening process: 1) title and abstract screening; and 2) full-text screening. First, two authors (CW and FP) in parallel sifted titles and abstracts of all the articles resulting from the searches to ascertain their potential relevance, with disagreements resolved by a third author (MH or VMD). All the full-texts of the potentially relevant citations were further examined in parallel by two authors (pairings amongst CW, FP, or MH) to analyse whether they met all the inclusion criteria. Disagreements were resolved by peer discussion and a third view from the project lead (VMD) if required.

Peer-reviewed articles were considered for analysis if they fitted the following inclusion criteria:

- Population: Physician Associates (PAs) according to the UK definition [23]
- Intervention: The implementation of PAs in the following secondary health care specialties: acute medicine, care of the elderly, emergency medicine, mental health, and trauma and orthopaedics (see supplementary file 2 for the definitions used).
- Comparison: The comparison group was any health care professional to whom PAs were compared.
- Outcome: Any measure of impact, informed by recognised dimensions of quality - effectiveness, efficiency, acceptability, access, equity and relevance.[24]
- Study design: Any study design that allowed measurement of impact of PAs in secondary care utilising a primary study.

Screening exclusion criteria

Articles were excluded if they did not fulfil one or more inclusion criteria or if they: 1) were not published in the English language, 2) reported on PAs working in countries that are not defined by the International Monetary Fund as advanced economies;[25] 3) did not report

For peer review only - http://bmjopen.bmj.com/site/about/guidelines.xhtml

empirical findings or were published only in abstract form; 4) presented their results for PAs in an amalgamated form with the results for other professions/mid-level providers or did not describe the specialties they were reporting on; 5) contained only descriptive accounts of PA demography, workload, clinical practice or productivity or PA self-report of any aspect of their role; 6) focused on and measured an intervention delivered by PAs rather than PAs as the intervention; 7) focused on and measured PA clinical practice or productivity before and after a service redesign or educational intervention; 8) focused solely on educational processes; and 9) presented literature reviews, commentaries, and/or non-peer-reviewed articles.

Data collection and quality assessment

Two authors (pairings amongst FP, CW and MH) independently extracted the data from selected papers, with any disagreement resolved through discussion. A checklist was used to extract the following information from the selected papers: 1) general characteristics of studies and 2) results, limitations and conclusions as noted by authors and reviewers.

The same author pairings appraised the quality of included studies using the QualSyst quality checklists for quantitative and qualitative studies, selected as a validated tool for the evaluation of primary research papers from a variety of fields,[26] with additional questions from the Mixed Methods Appraisal Tool, selected as a tool tested for its efficiency and reliability,[27] where appropriate. For the quantitative studies, 12 items (Figure 1) were scored depending on the degree to which the specific criteria were met ("yes" = 2, "partial" = 1, "no" = 0). Scores for the qualitative studies were calculated in a similar fashion, based on the scoring of ten items. Any items not applicable to a particular study design were marked "n/a" and were excluded from the calculation of the summary score. No study was excluded on the basis of its quality score; the limitations of lower quality evidence are however

For peer review only - http://bmjopen.bmj.com/site/about/guidelines.xhtml

explored in considering how much weight can be given to the evidence when we synthesise studies. [28]

Data analysis

A meta-analysis was not performed due to the heterogeneity of the included studies in terms of scope and outcomes investigated as found during data extraction. Therefore, narrative synthesis was undertaken [29] conducted against the four elements in published, accepted guidance on the conduct of narrative synthesis in systematic reviews [30,31]: developing a theory of how the intervention works, why and for whom; developing a preliminary synthesis of findings of included studies; exploring relationships within and between studies; assessing the robustness of the synthesis (through formal quality assessment as well as reflection). For the synthesis the included studies were grouped into specialty (that is, acute medicine, care of the elderly, emergency medicine, mental health and trauma and orthopaedics) and then sub-grouped into the outcomes they measured.

RESULTS

Search results

The overall search strategy identified 5,472 references, from which we selected 161 articles for more detailed reading. Figure 2 presents the PRISMA flowchart, illustrating the literature search and selection process, and reasons for study exclusion on full text reading. A total of 16 articles were included for data collection, quality appraisal and data analysis.

A summary of the included evidence is presented below in three subsections: characteristics of included studies, methodological quality, and synthesis of findings on the impact of PAs.

For peer review only - http://bmjopen.bmj.com/site/about/guidelines.xhtml

Characteristics of included studies

Table 1 presents the characteristics for each study in terms of the specialties they were drawn from.

For peer review only

For peer review only - http://bmjopen.bmj.com/site/about/guidelines.xhtml

245 **Table 1: Characteristics of studies included in full – studies presenting comparisons of PAS with other health care professionals**

246

Specialty	Aim(s)	Study Setting	Intervention	Comparison	Participants	Study design	Outcome measures	First author and year
Emergency medicine	To determine whether PAs are an appropriate option for providing services rendered by physicians in the ED	USA<br><br>Walk in urgent care facility (satellite of an inner-city teaching hospital level 1 trauma centre)	PAs (n=5) rotate through the ED. PAs work solo from 08.00-12.00. No written diagnostic or therapeutic guidelines were followed.	25 physicians rotate through the ED. Physicians work solo from 17.00-21.00. No written diagnostic or therapeutic guidelines were followed.	n= 5345 (seen by PAs)<br>n = 4256 (seen by physicians) during times of single coverage June 1995-June 1996	Comparative<br><br>retrospective	• Length of visit<br>• Total charge	Arnopolin 2000[32]

For peer review only - http://bmjopen.bmj.com/site/about/guidelines.xhtml

Emergency medicine	To examine the impact of PAs and nurse practitioners in EDs	Canada<br><br>Six community hospitals with ED volumes between 23 and 66,000	PAs were introduced as an unregulated provider without medical directives and worked under the supervision of a registered physician who was responsible for all patient care on predetermined busiest periods for each ED	Baseline two weeks	All ED patients: Baseline n=9,585; two week period six months post implementation June 2007 n=10,007, of which PAs were on duty for 1,076 visits and directly involved in n=376	Descriptive retrospective	• Leaving without being seen<br>• Wait time (triage to initial assessment)<br>• Length of stay in ED	Ducharme 2009[33]

For peer review only - http://bmjopen.bmj.com/site/about/guidelines.xhtml

Emergency medicine	To understand trends in emergency medicine and interprofessional roles in delivering this care […] The focus was on how doctors, PAs and nurse practitioners share emergency medicine visits	USA<br><br>National sample EDs of non-institutional general and short-stay hospitals in the 50 States and the District of Columbia from the National Hospital Ambulatory Medical Care Survey	PAs as providers of ED care and prescribers of medication in emergency medicine (7.9% of patients seen by PAs in 2004)	Physicians and Nurse Practitioners	Random sample of patient visits to hospital EDs (n= 1,034,758,313), 1995-2004	Longitudinal	• Proportion of visits in which medications are prescribed<br>• Mean number of prescriptions written per visit<br>• Non-narcotic analgesics prescriptions<br>• Narcotic analgesics/NSAIDS prescription by type of provider<br>• Patient contact growth by provider	Hooker 2008[34]

For peer review only - http://bmjopen.bmj.com/site/about/guidelines.xhtml

Emergency medicine	To compare the analgesic practices of emergency physicians with that of PAs	USA<br><br>ED within a suburban teaching hospital in Michigan with 90,000 annual visits	PAs were deployed for seeing patients presenting at the ED with isolated lower extremity trauma. PAs work closely with emergency physicians in the Prompt Care Area of the ED	Emergency physicians	n=384 survey respondents of patients of all ages who presented at the ED with an isolated lower extremity injury evaluated with a foot or ankle radiograph, n=227 PA patients, n=153 emergency physician patients in a nine week period	Prospective cohort	• Analgesia prescribing	Kozlowski 2002[35]
Emergency medicine	To evaluate PAs' management of paediatric patients in a general ED through examination of the 72-hour recidivism rates of their younger paediatric patients	USA<br><br>General urban ED treating approximately 58000 patients annually, 20% of which are under 18 years	PAs evaluate, treat and discharge patients of any age independent of emergency physicians and PAs treating patients with consult from the emergency physician	Attending emergency physician only	n=2798 PA only cases; n=984 PA with emergency physician; n=6587 emergency physician only	Comparative retrospective	• 72-hour revisits to the ED	Pavlick 2017[36]

For peer review only - http://bmjopen.bmj.com/site/about/guidelines.xhtml

Emergency medicine	To compare the quality of ED pain management before and after implementation of the Joint Commission on the Accreditation of Healthcare Organizations' standards in 2001	USA<br><br>National sample EDs included in the National Hospital Ambulatory Medical Care Survey	The use of PAs in the care of patients presenting to the ED with a long bone fracture	Patients presenting to the ED with a long bone fracture not seen by PAs (medical residents, internists)	n=2064 Patients presenting at the ED with a long bone fracture (femur, humerus, tibia, fibula, radius, or ulna) in two time periods: 1998-2000, n= 834 of which 3% were seen by a PA, 9% by resident/intern and 90% by staff physician ; 2001-2003 8% PA, 10% resident/intern, 90% staff physician	Retrospective cohort	• Proportion of patients with long bone fracture receiving analgesia	Ritsema 2007[37]

For peer review only - http://bmjopen.bmj.com/site/about/guidelines.xhtml

Emergency medicine	To compare the wound care practices and infection rates of wounds managed in the ED by practitioners with varying levels of medical training.	USA<br><br>Department of Emergency Medicine within a teaching hospital in New York	All patients with lacerations were evaluated by an attending physician who determined whether wound could be managed by a junior practitioner (PAs, students, interns, and residents)	ED patients whose wounds were managed by other providers (students, interns, and residents)	All patients with lacerations attending the ED n=1163, n=901 seen by a PA, n=262 by other providers October 1992 – November 1993	Prospective observational	• Patient wound infection rate	Singer 1995[38]

For peer review only

Trauma and orthopaedics	To define the clinical and financial impact of hospital-based PAs on orthopaedic trauma care at a level II community hospital.	USA<br><br>Orthopaedic trauma care at a level II community hospital.	Hospital-employed PAs (n=2) were utilised to cover all orthopaedic trauma needs, under the supervision of one of 18 orthopaedic surgeons. Each PA performed 12-hour day shifts for three consecutive days, January to December 2007. PAs on call carried trauma pagers and reported to the emergency room as soon as possible.	Attending surgeon as the primary orthopaedic responder for emergency department consults	n=1104<br>• n=310: PA<br>• n=687: No PA	Comparative retrospective	• Triage time to time seen by orthopaedic service in emergency department (minutes)<br>• Triage time to time of surgery (minutes)<br>• Operating room complication rates (%)<br>• The use of deep vein thrombosis prophylaxis (%)<br>• Post-operative antibiotic administration (%)<br>• Postoperative complications (%)<br>• Triage time to out of emergency department (minutes)<br>• Operating room set up time (minutes)<br>• Average operating room time (minutes)<br>• Time from wound closure to wheels out (operating room) (minutes)<br>• Hospital length of stay (minutes)<br>• Cost savings (emergency department) ($)	Althausen 2013[39]

For peer review only

For peer review only - http://bmjopen.bmj.com/site/about/guidelines.xhtml

Trauma and orthopaedics	To describe the effect of PAs working in an arthroplasty practice from the perspective of patients and health care providers<br>To describe the costs, time savings for surgeons and effects on surgical throughput and waiting times	Canada<br><br>High-volume academic arthroplasty programme employing PAs (The Concordia Joint Replacement Group)	Addition of PAs (n=3) to the operating room team. The PAs were added to the team, replacing surgical assists (usually general practitioners). The PAs took first call with their supervising physician, provided first-assist services in the operating room (OR), write postoperative tests/investigations, generate operative notes, undertake daily working rounds and complete discharge summaries.	-Costs: GP first assists in the operating room<br><br>-Waiting times: Patients on the arthroplasty waiting list in 2004 and 2005	Sample size varying by outcome:<br>-Patient satisfaction n=1070<br>-Perceptions of healthcare providers and patients n=44<br>-Costs n=402 surgical procedures performed in 2006<br>-Time savings n=1409 procedures carried out 2006<br>-Waiting times in 2006	Mixed-methods	• Patient satisfaction<br>• Perceptions of PAs among healthcare providers and patients<br>• Costs<br>• Time savings<br>• Waiting times<br>• Throughput	Bohm 2010[40]
Trauma and Orthopaedics	To assess whether the type of	USA<br><br>Children's	PAs carrying out nonoperative management of	Attending physician	Patient charts of those aged 3 to 17 years	Comparative retrospective	• Fracture malunion (maximum angulation criteria) at last clinic	Garrison 2017[41]

For peer review only - http://bmjopen.bmj.com/site/about/guidelines.xhtml

	provider (attending physician versus PA) or number of providers involved in the nonoperative management of a paediatric forearm fracture influenced the risk of that fracture healing as a malunion.	hospital medical centre	forearm fractures at orthopaedic clinic visits		seen at the orthopaedics department February 2012 to January 2013 n = 141		visit	
Trauma and Orthopaedics	To describe the role of the PA in the upper-extremity surgical programme; describe the role of the PA in an operating room study; and show the	Canada<br><br>Subspecialised upper-extremity surgical programme at a peripheral hospital, as part of a Physician Assistant Demonstration project where 12 PAs were	One PA filling provider gaps in four areas: preoperative patient screening, assisting in operating room care (including a double room experiment), aiding in aftercare of surgery and attending to post	Pre-operative – surgeon working alone; operating room – team with surgical assistant or role unfilled and single operating room; surgery aftercare –	n=38 interviews; n=75 surveys (n= 28 from health care provider and 47 from patients	Mixed methods	• Perceptions and experiences with the PA<br>• Patient rating of quality of care<br>• Expected and actual operating room times<br>• Total new patients seen	Hepp 2017[42]

For peer review only - http://bmjopen.bmj.com/site/about/guidelines.xhtml

	impact of the PA role on patients, providers and the system	introduced to various healthcare settings	discharge follow up care	replacing a post unfilled surgical extender; post discharge – surgeon only				
Trauma and orthopaedics	To assess whether staffing changes within a Level 1 trauma centre improved mortality and shortened hospital and ICU length of stay for patients with trauma.	USA<br><br>Urban, community-based level I trauma centre	core trauma panel (consisting of full-time, in-house trauma surgeons) and PAs	Group 1: general surgery residents (staffed by full-time, in-house post-graduate year-4 general surgery residents with attending back up from home, followed by a transition to a trauma service staffed with in-house independent general surgeon attendings) ; Group 2: core trauma panel (consisting of	n=15297 Trauma patients 18 years or older and not transferred from the ED to another acute care facility	Prospective cohort	• Overall mortality<br>• Mortality for patients with injury severity score (ISS) >15<br>• Hospital LOS	Mains 2009[43]

For peer review only - http://bmjopen.bmj.com/site/about/guidelines.xhtml

				full-time, in-house trauma surgeons, without PAs or residents)				
Trauma and orthopaedics	To analyze patient outcomes and efficiency of care provided for trauma patients during transition from resident physician support to PA support	USA<br><br>Level I Trauma Center	PAs substituting for doctors in trauma alerts: PA's role was to assist the trauma surgeon at trauma alerts and trauma patient rounds, update the trauma patient census list	General and orthopaedic residents who attend in trauma alerts	n=293-before n=476-after All patients evaluated by the trauma surgeons and on the trauma registry, excluding those transferred to another facility for treatment of severe burns	Before-after	• Collaborative relationship<br>• Transfer time<br>• LOS<br>• Mortality rate	Oswanski 2004[44]
Internal medicine	To compare outcomes directly from the expanded use of PAs to those of a hospitalist group staffed with a greater proportion of attending	USA<br><br>Community hospital with 26,000 adult patient discharge annually	Expanded PA group: used three physicians and three PAs daily for ward rounds with PAs expected to see 14 patients daily plus one more PA responsible for day shift	Conventional group: Used nine physicians and two PAs for rounding, with PAs expected to see nine patient daily, plus day shift	Patients discharged between January 2012 and June 2013; n=6612 expanded PA group and n=10352 in the conventional	Retrospective comparative	• 30 day all-cause readmission<br>• Inpatient mortality<br>• Cost of care<br>• Consultant/attending use<br>• Length of stay	Capstack 2016[45]

For peer review only - http://bmjopen.bmj.com/site/about/guidelines.xhtml

	physicians at the same hospital during the same time		admissions. PAs worked in dyads with ward round physician; PAs discussed the treatment plans at least once a day with the physician to a written protocol for PA-physician dyad expectations	admissions by the physician. PAs worked in dyads with ward round physician; PAs discussed the treatment plans at least once a day with the physician. No written protocol for PA-physician dyad expectations	group			
Internal medicine	To examine and compare costs, between a PA service and an intern/resident (teaching) service in the provision of inpatient care for five high-volume internal medicine	USA<br><br>Two general internal medicine units, teaching hospital	The use of PAs (n=16) in the provision of care within internal medicine department (64 attending physicians on rotation coverage, scheduled to admit to either a PA or teaching service, with group assignment determined one	The teaching service (32 intern/residents with an average experience of one year post-medical school)	Adult patients discharged in the following diagnostic-related groups: cerebrovascular accident/stroke, pneumonia, acute myocardial infarction discharged alive, congestive	Prospective cohort study	• Relative value units (costs)<br>• Length of stay	Van Rhee 2002[46]

For peer review only

For peer review only - http://bmjopen.bmj.com/site/about/guidelines.xhtml

	diagnostic related groups		year in advance).		heart failure, gastro-intestinal haemorrhage: n=923, of which n=409 PA and n=514 teaching service			
Mental health	To examine the role of PAs in the care of patients with severe and persistent mental illness	Canada<br><br>Assertive community treatment team, providing multidisciplinary care to patients with severe and persistent mental illness	A PA was hired to assist with intake psychiatric assessments, physical examinations, preventive care, and follow-up of psychiatric and medical complaints in a model of PA supervised by a psychiatrist	No comparison	Assertive community treatment team members (three social workers, one psychiatrist, two psychiatric nurses, one occupational therapist, one recreational therapist, the PA)	Qualitative interview	• Perceived effect and challenges of delivering psychiatric care with the PA model	McCutchen 2017[47]

For peer review only - http://bmjopen.bmj.com/site/about/guidelines.xhtml

In summary, seven studies were included from emergency medicine,[32-38] six studies reported from trauma and orthopaedics,[39-44] two from acute internal medicine,[44,45] and one from mental health.[47] No studies were identified from care of the elderly medicine.

.

The publication year ranged from 1995[38] to 2017,[36,41,42,47]. The majority were from the USA (n=12), with four from Canada.[32,38,42,47]. The studies measured a number of outcomes; results are shown in Table 2.

For peer review only

For peer review only - http://bmjopen.bmj.com/site/about/guidelines.xhtml

Table 2: Main findings of included studies

Specialty	Outcome measures	Finding(s)	Quality score	Key limitations	Study details
Emergency medicine	Length of visit (LOV)	Small but clinically insignificant differences (regression coefficient -8): LOV was 8 minutes longer when patients were treated by a PA (mean 82 minutes) than a physician (mean 75 minutes) (95% CI -10 to -6, p<0.001), although difference ranged from 5 to 32 minute difference dependent on patient condition	82%	• Not randomised<br>• Differences by patient condition not explained<br>• Limited control for confounders	Arnopolin 2000[32]
	Total charge	Mean total charge was $159 when patients were treated by a PA and $164 by a physician (95% CI: 2 to 14, p=0.013), regression coefficient -8			
Emergency medicine	Leaving without being seen	Absolute improvement (not controlling for hospital or acuity) from 6.5 to 4.9%; when a PA was on duty, the likelihood that a patient left without being seen was less than half (44% [95% CI 31% to 63%] p < 0.01), controlling for hospital and patient acuity	73%	• Two months data<br>• Sample size unclear	Ducharme 2009[33]
	Wait time (triage to initial assessment)	When a PA was involved in patient care, the odds of the patient being seen within the benchmark wait time was 1.6 times greater than when the PA was not involved (95%CI 1.3 to 2.1) p <0.05, adjusting for hospital, acuity and time of day			
	LOS in ED	When a PA was involved in patient care, the LOS in the ED was shorter (mean: 262.4 mins versus 182.9 mins) than when a PA was not present (30.3% [95% CI 21.6% to 39%]), p < 0.01			
Emergency medicine	Proportion of visits in which medications are prescribed	Significant differences were observed between PAs if compared to physicians and to NPs in the proportion of visits in which medication was prescribed: PAs 77.9%, physicians 75.5%, nurse practitioners 75.4% (p=0.001)	73%	• Secondary data analysis<br>• No adjustment<br>• Treatment outcomes/appropriate	Hooker 2008[34]
	Mean number of	There were no significant differences among the three providers in mean			

For peer review only - http://bmjopen.bmj.com/site/about/guidelines.xhtml

Specialty	Outcome measures	Finding(s)	Quality score	Key limitations	Study details
	prescriptions written per visit	number of prescriptions per visit (PA and physician 1.7, nurse practitioner 1.6)		ness not assessed	
	Non-narcotic analgesics prescriptions	There were no significant differences among the three providers in the frequency of prescribing non-narcotic analgesics (p=0.16).			
	Narcotic analgesics/NSAIDS prescription by type of provider	There were no significant differences among the three prescribers in the frequency of narcotic analgesics or NSAIDS recorded (p=0.15 and p=0.06, respectively)			
Emergency medicine	Analgesia prescribing	Emergency physicians gave some form of ED analgesia to 29% of patients, as compared with 10% of patients seen by PAs (OR=3.58 [CI 95% 2.05 to 6.24]), adjusting for sex, reported degree of pain and fracture	92%	• Dependent on patient recall	Kozlowski 2002[35]
Emergency medicine	72-hour revisits to the ED	Patients treated only by PAs had significantly lower return rates (6.8%) than for the PA/emergency physician combined group (9.3%) and the emergency physician only group (8.0%), p=0.03.	77%	• No adjustment for significant differences in patient age, admission rate or patient complexity	Pavlick 2017[36]
Emergency medicine	Proportion of patients with long bone fracture receiving analgesia	Patients seen by PAs had more than twice the odds of receiving opiates/narcotics (OR=2.05% [95%CI 1.24 to 3.29]) and were more likely to receive other analgesics (OR=1.72% [95%CI 0.94 to 3.17]) compared with those not seen by PAs	100%	• Changes in workload and documentation could have confounded results	Ritsema 2007[37]
Emergency medicine	Patient wound infection rate	There were no significant differences in wound infection rates by practitioner level of training (medical students, 0/60[0%]; all residents, 17/547[3.1%]; physician assistants, 11/305[3.6%]; and attending	67%	• Hawthorne effect<br>• Differences in wounds not controlled for	Singer 1995[38]

For peer review only

For peer review only - http://bmjopen.bmj.com/site/about/guidelines.xhtml

		physicians 14/251[5.6%]; p=0.14)			
Trauma and orthopaedics	Triage time to time seen by orthopaedic service (emergency department) (mins)	PA presence resulted in a 205 minutes faster orthopaedic service response time (366 versus 571 mins; p=0.0006)	91%	• Exact cost savings difficult to determine<br>• Did not have a way of calculating savings for the time it took for patients to reach the OR from the time of triage<br>• Single site with two PAs	Althausen 2013[39]
	Triage time to time of surgery (ER) (mins)	PA presence resulted in a 360 minutes improvement in time to surgery (1139 versus 1499 mins; p=0.03)			
	Operating room complication rates (%)	There was no significant difference in the proportion of operating room complications with or without PAs (both 0.65%; p=0.9972)			
	The use of deep vein thrombosis prophylaxis (%)	The use of deep vein thrombosis prophylaxis increased by a mean of 6.73 percentage points (60.69 versus 53.96%; p = 0.0084) with PA presence.			
	Post-operative antibiotic administration (%)	Post-operative antibiotic administration increased by 2.88 percentage points with PA presence (94.35 versus 91.47%; p=0.0302)			
	Postoperative	There was a 4.67 percentage points decrease in postoperative			

For peer review only - http://bmjopen.bmj.com/site/about/guidelines.xhtml

	complications (%)	complications with PA presence (8.16 versus 12.83%; p=0.0034)			
	Triage time to out of emergency department (mins)	There was a 176 minutes decrease in total ER time with PA presence (270 versus 446 mins; p<0.001)			
	Operating room set up time (mins)	There was a marginally improved operating room set up time by 0.43 minutes with PA presence (26.6 versus 24 mins; p=0.0034)			
	Time from wound closure to wheels out (operating room) (mins)	There was no significant difference for this outcome when the PA was present (7.8 versus 7.6 mins; p=0.5914)			
	Average operating room time (mins)	There was no significant difference in the average operating room time when the PA was present (70 versus 74 mins; p=0.44)			
	Cost savings (emergency department) ($)	Based on 50% collection of PA charges and emergency department time savings, per orthopaedic trauma patient seen, PAs saved the hospital $133.53 per patient, resulting in $41,394 in one year (310 patients)			
	Cost savings (operating room) ($)	The presence of a PA in the operating room resulted in savings of $3,207 based on operating room costs (only set up time was decreased with presence of the PA).			
	Hospital length of stay (days)	There was no significant difference in the hospital LOS when the PA was present if compared to when the presence and the absence of PAs (7.96 versus 8.57 days; p=0.2662)			
Trauma and orthopaedics	Patient satisfaction	91.3% of hip patients (total= 626, 58.5% response) reported being satisfied or very satisfied and 87.7% of knee patients reported being satisfied or very satisfied with PAs at one year follow-up (after surgery)	32%	• Methods are not fully described e.g. no description of data analysis<br>• Sample is not described<br>• Is this a study about PAs or about the two	Bohm 2010[40]
	Perceptions of healthcare providers and patients about PAs	Patients: Overall patients expressed very positive opinions of PAs who were helpful in providing information and explaining aspects of their care<br>Ward nurses: felt that patient care, information flow and patient rounds were enhanced by the PAs; ambiguous as to whether PA tasks fell within the scope of nursing			

For peer review only

For peer review only - http://bmjopen.bmj.com/site/about/guidelines.xhtml

		Orthopaedic surgeons: overall the surgeons had very positive opinions of PAs – 100% agreement with all survey items: 'a fully trained PA provides surgical assistance equal to an R5 (fifth year of a residency programme)'; 'the presence of PA has improved your job satisfaction'; 'the presence of a PA has safely allowed you to do more surgical volume'; 'the care of your patients in the OR is improved by the assistance of PAs'; 'PAs greatly decrease the amount of "scut work" that you have to do'<br>Operating room nurses: overall OR nurses reported that PAs were valuable team members; improved the care of orthopaedic surgery patients in the operating room; provided surgical assistance superior to family practitioners; and were necessary to run two operating rooms<br>Orthopaedic residents: nearly unanimous that PAs reduced their workload and they generally felt that PAs relieved them of clinical responsibilities so that they could attend teaching.		room operating model?<br>• Patient satisfaction with the surgery at one year cannot be attributed to the PA	
	Costs	The cost of employing three PAs in 2006 (between $270,000 AND $327000) was found to be similar to the foregone general practitioner (GP) surgical assist fees of $270226.88.			
	Time savings	PAs were found to "free up" 204 hours per year (the equivalent of four 50-hour work weeks), for their supervising physician (p=not reported). Furthermore, they potentially freed GPs from the operating room to spend more time delivering primary care			
	Throughput	Increased the volume from three to seven primary joint surgeries per day through the use of double rooms in 2006			
	Waiting time	Median wait time for surgery decreased from 44 to 30 weeks			
Trauma and orthopaedics	Fracture malunion (maximum angulation criteria) at last clinic visit	Likelihood of malunion did not differ significantly if the providers included a PA or not (28% versus 56%, Fishers exact p=0.13) or by number of PAs (p=0.11).	82%	• Unadjusted comparisons<br>• Difficult to assess how much of the care was carried out by	Garrison 2017[41]

For peer review only - http://bmjopen.bmj.com/site/about/guidelines.xhtml

				PAs (analysis is cases with any PA involvement versus cases with no PA involvement)	
Trauma and orthopaedics	• Perceptions and experiences with the PA	• Preoperative care: PA triages, conducts most activities without direct supervision<br>• Operating room: PAs' integration into the OR went well; staff appreciate consistency of the PA; PA acquired skills in a graduated manner – now "preps and closes with patients in OR"<br>• Postoperative care: takes on some of surgical extender role but the role is missed after hours; PA sees 60-70% of all inpatients, freeing up the surgeon; full integration limited by needs for co-signature and verification of orders<br>• Follow-up outpatient care: clinic flow improved<br>• PA is a collaborative member of the team (most mean ratings >4 out of 5.	55%	• Unable to ascertain which data are descriptive quantitative or gained from qualitative interviews	Hepp 2017[42]
	• Patient rating of quality of care	All patients responded positively to the PA role; overall rating of PA care of 9.65 of 10			
	• Expected and actual operating room times	Double room experiment: actual preparation time 39% longer than expected and postsurgery time 37% less than expected (absolute times not given) surgeon time 21% less; two hour/day saving			
	Total new patients seen	Preoperative care: 30% increase in numbers of patients seen, noticed in the first year			
Trauma and orthopaedics	Overall mortality	The introduction of PAs to the core trauma panel (group 3 versus group 2) decreased overall mortality (2.80% versus 3.76%, adjusted OR=0.74 [CI95% 0.55 to 0.99], p=0.05). Furthermore, the introduction of PAs to general surgery residents (group 3 versus group 1) decreased overall mortality (2.32% versus 3.82%, adjusted OR=0.6 [CI95% 0.45 to 0.81], p=0.003)	100%	• Not all the covariates which could be significantly associated with outcomes were	Mains 2009[43]

For peer review only - http://bmjopen.bmj.com/site/about/guidelines.xhtml

	Mortality for patients with injury severity score (ISS) >15	The introduction of PAs to the core trauma panel (group 3 versus group 2) decreased overall mortality for patients with injury severity score (ISS) >15 (9.67% versus 12.21%, adjusted OR=0.77 [CI95% 0.55 to 0.99], p=0.13). Furthermore, the introduction of PAs to general surgery residents (group 3 versus group 1) decreased overall mortality in this patients (9.03% versus 14.83%, adjusted OR=0.6 [CI95% 0.41 to 0.80], p=0.003)		collected (e.g. changes in care)<br>• The group 1 period was characterised by a transition from on-call attending surgeons to in-house surgeons and the outcomes may not be homogenous across the study period<br>• Other changes were made, not just individual staff type	
	Hospital LOS	The introduction of PAs to the core trauma panel (group 3 versus group 2) reduced mean and median hospital LOS (4.32 days versus 4.69 days, p=0.05; and 3.74 days versus 3.88 days, p= 0.02, respectively). As well, the introduction of PAs to general surgery residents (group 3 versus group 1) reduced mean and median hospital LOS (4.32 days versus 4.62 days, p=0.05; and 3.74 days versus 3.94 days, p= 0.003, respectively)			
Trauma and orthopaedics	Collaborative relationship	Participation during trauma alert calls: PA 100%; resident 51% overall, 88% during on duty hours; Involvement in minor procedures PA 100% when residents off-duty, 91% overall; resident 95% during on duty hours, 83% overall.	82%	• Investigators not blinded and all work in the trauma centre investigated.<br>• No sample size calculation<br>• Single site with two PAs<br>• Minimal description of data collection method	Oswanski 2004[44]
	Transfer time	After controlling for age, gender, race and severity of illness, there was no significant difference in the mean transfer rate overall or for any subpopulation (destination) between years 1998 and 1999			
	LOS	After controlling for age, gender, race and severity of injury, there was no significant difference in the mean LOS overall between years 1998 and 1999			
	Mortality rate	Mortality rate for all patients admitted to the trauma service was 2.2% for both 1998 (8/293) and 1999 (13/479)			
Internal medicine	30 day all-cause readmission	No statistically significant difference in odds of readmission between expanded PA (14%) and conventional PA (13.7%) groups (OR 0.95 [95% CI, 0.87 -1.04]; p=0.27)	91%	• Non randomised patient allocation	Capstack 2016[45]

For peer review only - http://bmjopen.bmj.com/site/about/guidelines.xhtml

	Inpatient mortality	No statistically significant difference in odds of mortality between expanded PA (1.3%) and conventional PA (0.99%) groups (OR 0.89 [95% CI, 0.66 -1.19]; p=0.42)		• Use of secondary data<br>• Readmission to the same hospital only	
	Cost of care	Statistically significant difference in mean patient charge between expanded PA ($7822) and conventional PA ($7755) groups (3.52% lower [95% CI, 2.66% -4.39%]; p<0.001)			
	Consultant use	No statistically significant difference in utilisation of consultants between expanded PA (1.3%) and conventional PA (0.99%) groups (OR 1.0 [95% CI, 0.94 -1.07]; p=0.90)			
	Length of stay	No statistically significant difference in length of stay between expanded PA (4,1 +/- 3.9 days) and conventional PA (4.3 +/- 5.6 days) groups (effect size, 0.99 days shorter [95% CI, 0.97-1.01 days]; p=0.90)			
Internal medicine	Relative value units (RVUs i.e. costs)	1) Radiology RVUs: There were no statistically significant differences between PAs and residents; 2) Total RVUs (excluding pharmacy data): PAs used significantly fewer resources when compared to resident services for pneumonia care (p = .004), although had a higher mortality rate (% and p value not reported). For all other diagnoses there were no statistically significant differences in total RVUs between PAs and residents; 3) Laboratory RVUs: There were statistically significant differences between PAs and residents in laboratory relative value units for stroke (p = .015), pneumonia (p = .003) and CHF (p = .004). In each case PAs' RVUs were lower than those of residents.	86%	• RVU figures are not explained<br>• Non-random group assignment<br>• Single centre	Van Rhee 2002[46]
	Length of Stay (LOS)	There were no significant differences in LOS between PAs and residents after adjusting for admitting physician effect and other covariates			
Mental health	Perceived effect and challenges of delivering psychiatric care with the PA model	Participants described: improved access to primary care for patients; more timely access to psychiatric appointments and longer appointments; equal team cohesion for the PA or the psychiatrist; decreased wait times and improved access to tertiary care and screening programmes; and implementation challenges of triage hierarchy and patient understanding of the term physician assistant	45%	• Qualitative analysis methods described without detail<br>• Short report with overview of themes; no quotations	McCutcheen 2017[47]

For peer review only - http://bmjopen.bmj.com/site/about/guidelines.xhtml

For peer review only

For peer review only - http://bmjopen.bmj.com/site/about/guidelines.xhtml

Two studies employed mixed methods [40,42]; one study used a qualitative analysis [47], the remainder employed quantitative approaches. Five quantitative studies analysed prospectively collected data [35,38,40,43,46,] and seven used a retrospective analysis.[32,33,34,36,39,42,45]. All studies but one [46] were observational.

Methodological quality

The studies were of variable methodological quality. The mean quality score was 79% (SD 0,20), median 82%, minimum 32%, [40] maximum 100%, [37,43] IQR 73,92. Figure 1 presents a summary of the degree to which the included evidence met the criteria of methodological quality and shows that the most important methodological flaws in the included quantitative studies were the failure to adjust the analysis for confounding variables, the absence of information to evaluate participants' selection adequacy, and the lack of information about baseline and/or demographic information of the investigated participants. Overall, the quality of the included qualitative evidence was low, mainly due to insufficient description of the sampling strategy, data collection and analysis methods.[40,44,47]

Synthesis of findings on the impact of physician associates

We organised our findings by secondary care specialty. Within each specialty, we described the findings within the quality dimensions, [24] presenting the dimension with the largest number of studies within each specialty.

Emergency medicine

The seven studies in emergency medicine variously compared clinical care offered by PAs and physicians of various grades[34,35, 36,37] and operational/service measures.[32,33] In only two of these studies was the comparison of PAs and other physicians in a system where

For peer review only - http://bmjopen.bmj.com/site/about/guidelines.xhtml

the PAs were described as working 'solo', substituting for physicians at particular times of the day[32] or seeing patients without the input of the attending physician.[36]

Waiting or access_outcomes were reported in one Canadian study; [33] the outcomes were leaving without being seen and waiting times. The presence of a PA was reported as significantly reducing the likelihood of a patient leaving without being seen by 44% (95% CI 31% to 63%, p < 0.01), the crude rate being 6.5 without and 4.9% with a PA). The odds of a patient being seen within their benchmark wait time was 1.6 times greater (95% CI 1.3 to 2.1, p <0.05) when the PA was involved in the patient's care, with these analyses strengthened by adjustment for hospital, time of patient visit and acuity level.[33] However, the PA was an additional staff resource rather than a substitute in this study, giving extra coverage at the busiest times, alongside also newly appointed nurse practitioners, who increased the odds of being seen on target more than the PAs did, with an odds ratio of 2.1.

Length of stay was considered in two studies,[32,33] with contradictory results in the comparison against physicians, from different interventions in terms of PAs. Arnopolin and Smithline (2000)[32] reported experienced ED PAs and physicians working solo at different times of day in a satellite unit. This study provided a direct comparison (and control for patient age in the analysis), with a result of a statistically significantly mean longer length of visit (eight minutes) for patients of PAs (82 minutes versus the physicians' 75 minutes, 95% CI -10 to -6, p<0.001), but also noted that differences in length of visit varied by diagnostic group, with PAs' patients between five and 32 minutes longer. In contrast, Ducharme et al[33] reported that where PAs were an additional staff resource alternating with nurse practitioners, PAs reduced their length of stay by 30% (mean 80 minute reduction, 183 minutes versus 262 minutes, 95% CI 21.6% to 39%, p < 0.01) .

For peer review only - http://bmjopen.bmj.com/site/about/guidelines.xhtml

Cost was considered through total charge (hospital and physician charge) for the visit, [32] with a small but statistically significant decrease per patient reported when patients were treated by a PA, with differences (not statistically significant) by diagnostic groups.

Treatments offered, in terms of analgesia prescribing, were reported in three studies, [34,35,37] with conflicting findings. Secondary analysis of national (USA) ED survey data (1995 to 2004) reported no significant difference by type of provider in frequency of prescribing narcotic or non-narcotic analgesics and in the mean number of prescriptions per visit, but did observe a statistically significantly higher proportion of PAs' cases receiving a prescription compared with those of physicians and nurse practitioners (PAs 77.9%, physicians 75.5%, nurse practitioners 75.4%, p=0.001).[34] No adjustment for potential confounders was made. Using the same national survey data but for a subset for long bone fractures, secondary analysis for 1998 to 2003 reported similarly, with those seen by a PA having adjusted odds of 2.05 for receiving opiate analgesia in the ED (95% CI 1.24 to 3.29).[37] This well powered retrospective cohort study of high quality differs from another study of similar quality with somewhat contrasting findings [35] in which for patients contacted at an undefined time (average three days following their ED visit) those attended by an emergency physician had adjusted odds of 3.58 (95% CI 2.05 to 6.24) for receiving pain medication while in the ED (29% of their patients) compared to those attended by PAs (10% of their patients), in a prospective cohort study based on patient self-report.[35] Although the period of time for this study is not specified, it first reported in 1998, perhaps suggesting the same decade of data was involved. These three studies did not report the PAs' place in the team or whether they added to or substituted for members of the medical team, nor whether they saw patients as part of a team or solo.

Two studies considered clinical outcomes of care. One, the oldest study in the review [38], from 1995, reported that in a large sample of patients presenting with lacerations at the ED

For peer review only - http://bmjopen.bmj.com/site/about/guidelines.xhtml

and seen by PAs there was no statistically significant difference in wound infection rates, , compared to other medical staff providers (medical students, residents and attending physicians).[38] However, the authors noted a potential Hawthorne effect as all wounds had been evaluated by an attending physician prior to allocation to one of the medical team members, based on their level of training. It was noted that PAs in this study, with nine to 12 years' experience, were classified as experienced (not junior) practitioners. The other, newer, study[36] used a proxy measure of clinical safety, that is the 72-hour re-attendance (recidivism) rate to the ED for children aged six and younger, and reports that this was significantly lower for those patients treated only by a PA (6.8% versus emergency physician 8.0%, p=0.03), in a large study. However, these rates were unadjusted, and the characteristics of the study population show statistically significantly different mean ages and rate of admission in the patients treated in each group, with PAs seeing the older of the children who were much less likely to be admitted. Although analysis of the recidivism rates by Emergency Severity Index score for patients seen by PAs versus doctors found no statistically significant differences between groups and the authors conclude that PA providers deliver comparable care; the authors themselves consider that it is not known if PAs would have made the same decisions as physicians for the same group of patients.

Trauma and orthopaedics

For peer review only - http://bmjopen.bmj.com/site/about/guidelines.xhtml

Six papers reported on PAs working in trauma and orthopaedics. These spanned a 14 year period. Four [39,41,43,44] focused on an aspect of provision of a hospital trauma service; and two considered planned inpatient care.[38,42]

Three studies described how PAs were substituting for doctors, for residents [44] or surgical assistants [40,42], whilst the others presented service re-organisations of which PAs were a part, seemingly an addition to the pre-existing medical team [39,41,43] The outcomes assessed were numerous - patient satisfaction, perceptions of other clinical staff, costs, time of various aspects of care, patient throughput, length of stay, fracture malunion and operative complications and mortality. The strength of evidence for each outcome is now assessed.

Two prospective studies of the addition of PAs to surgical teams, pre-, intra- and post-operatively [40,42] reported both patient satisfaction and acceptability of PAs to other clinical staff from surveys of these groups. Positive results were presented from both studies' patient satisfaction surveys, in large[40] and small[42] response numbers, reporting 91.3% of hip and 87.7% of knee patients being satisfied or very satisfied[40] and an overall rating of PA care of 9.65 out of 10[42] although no comparator data were collected. The reports of staff were more mixed by staff group in Bohm's study[40] with physician team members being positive (100% agreement with all survey items on the positive contribution of PAs) and nursing staff more equivocal, expressing concern about the overlap of tasks traditionally considered to be the responsibility of nurses; and by impact in different parts of the surgical journey in Hepp's[42] study, where staff ratings were mostly above four out of five, agreeing or strongly agreeing that the PA was a collaborative team member. Staff appreciated continuity and PA advances in skills in the operating room, but did not feel the role could

For peer review only - http://bmjopen.bmj.com/site/about/guidelines.xhtml

offer everything a previous surgical extender did post-operatively, despite being a collaborative team member.[42]

Operational measures were_addressed in five of the studies in this specialty, split into a number of outcomes pertaining to time [39,40,42-44] and to cost. [39,40]

The evidence of the impact of PAs on access times was equivocal. One study reported how the wait to be seen by the orthopaedic service in the emergency department section of their orthopaedic pathway were significantly shortened (366 minutes versus 571 mins; p=0.0006) when PAs were substituted directly for doctors , although the authors attributed this to a combination of factors, and not just to the PAs, including more registered nurse cover, introduction of a family practice resident and other changing practices.[39] Another found the same when PAs were added to the team as part of larger trauma team re-organisation.[39] Median number of weeks to wait for surgical procedures was also reported to be reduced from 44 to 30 weeks,[40] attributed by the authors to the use of two operating theatres by the surgeon, made possible by the PA preparing and finishing the case, similarly to the 30% increased throughput in the number of new patients in the pre-operative stage.[42]

In terms of time, two studies [39,42] reported in detail on operating room times– set up, wound closure to out of theatre, average operating room time, and post surgery time. Althausen et al[39] only noted a minimal (not statistically significant - (26.6 versus 24 mins; p=0.0034) difference for set up time in a direct comparison study, while Hepp[42] describes a 39% reduction in time at this stage. PAs also released time for supervising physicians – 204 hours per year (p=not reported)[40] or two hours a day,42] and for general practitioners (GPs) (not quantified), who had previously acted as surgical assistants [40], Three high quality studies [39,43,44] reported variably on length of hospital stay, with one showing a significant reduction (three to four hours, a fraction of one day) for all patients when PAs

For peer review only - http://bmjopen.bmj.com/site/about/guidelines.xhtml

were an addition to either the resident physician team (mean 4.32 days versus 4.62 days, p=0.05; and median 3.74 days versus 3.94 days, p= 0.003) or reorganised trauma panel (mean 4.32 days versus 4.69 days, p=0.05; and median 3.74 days versus 3.88 days, p= 0.02) [43] and two replacement studies finding no difference – when carrying out adjusted analyses of one year against another[44] or when PAs were present or not.[39]

Evidence regarding cost was again mixed. Bohm[40] suggests the actual costs of employment for three PAs (between $270,000 and $327000) were similar to those of the GPs they replaced ($270226.88) in the operating room but argue an opportunity cost for others through released time for the supervising physicians. However, a non-replacement model, Althausen[39] reported specific cost savings in the ED ($133.53 saving per patient, $41,394 in one year) and operating room ($3,207 saving) based on time reduction and PA charges (taking account that only 50% of PA costs were covered through charges).

As well as these operational measures, these studies also reported health outcomes, and all reported no difference[41] or improvement in these.[39,43,44] Two considered the rate of complication from procedures involving physician associates [37,41] and two reported on mortality.[43,44] In terms of operating room complication rates[39] or the likelihood of fracture malunion if the providers included a PA[41], these did not differ significantly from those of other providers, but postoperative complications were reported to have decreased (8.16 versus 12.83%, p=0.0034) and antibiotic use (94.35 versus 91.47%, p=0.0302) and DVT prophylaxis (60.69 versus 53.96%, p = 0.0084) increased (statistically significantly) for cases with a physician associate present (although it is noted that the tables in this paper presented findings contradictory to the text and abstract).[39] One study assessing mortality in two, year-long periods reported that involvement of PAs in the clinical team had no effect on overall mortality rates[44] while another found that mortality decreased by approximately one per cent with the introduction of PAs to a trauma panel (9.67% versus 12.21%, adjusted

For peer review only - http://bmjopen.bmj.com/site/about/guidelines.xhtml

OR=0.77 [CI 95% 0.55 to 0.99], p=0.13) and 1.5% to general surgery residents' teams (9.03% versus 14.83%, adjusted OR=0.6 [CI 95% 0.41 to 0.80], p=0.003).[43] However, this could not be directly attributable to the addition of the PA because contemporaneous improvements in efficiency of the trauma service occurred.

Acute internal medicine

The two studies considering PAs in acute internal medicine both examined resource use and clinical outcomes[45,46] in replacement studies, one prospectively examining the impact of PAs in place of interns/residents[46], the other retrospectively comparing outcomes where PAs made up a greater or lesser proportion of the medical team staff, in place of physicians.[45] Both studies measured length of stay, direct costs, and inpatient mortality for patients with diagnoses of cerebrovascular accident, pneumonia, acute myocardial infarction discharged alive, congestive heart failure and gastrointestinal haemorrhage[46] and those with a principal medical (non-surgical, non-obstetrical) diagnosis code.[45]; the latter study also measuring 30-day all cause readmission Neither study reported any significant differences in length of stay between groups, with length of stay considered to be a proxy for severity of illness. Cost in terms of relative value units (RVUs, based on billing information for physician-ordered items, excluding administrative costs outside of the physician's control) was also mostly similar although laboratory RVUs were lower for PAs, that is, they ordered fewer investigations after adjustment for demographics in each diagnostic group (for stroke, p = .015, pneumonia p = .003 and CHF p = .004). In each case PAs' RVUs were

For peer review only - http://bmjopen.bmj.com/site/about/guidelines.xhtml

lower than those of residents.[46] Similarly, Capstack et al[45] reported a statistically significantly lower mean patient charge for the expanded PA group ($7822 versus $7755 for the conventional PA group (3.52% lower [95% CI, 2.66% -4.39%]; p<0.001). Inpatient mortality was stated to be higher for the PA group in pneumonia care only[46], although the authors reported neither the percentage nor statistical values, and the larger study reported no significant differences in mortality or 30 day all-cause readmission.[45] The authors concluded that PAs used resources as effectively as, or more effectively than, residents[46] at the same time as providing similar clinical quality.[45]

DISCUSSION

Principal findings

This systematic review identified a large number of studies of PAs working in secondary care settings, internationally. However, once studies were excluded that did not meet the inclusion criteria, only 16 papers remained. Most of the included studies were from the emergency medicine and trauma and orthopaedics specialties, with two from acute internal medicine and one from mental health. We found no studies in our other specialty of interest – care of the elderly– where another larger grouping of PAs worked in the UK according to a national survey[18] at the time of planning this review. Several of the studies were of high quality, providing comparative data, and some contained statistical adjustments to address confounding; however all findings were observational. While we recognise that trials are rarely feasible in this type of workforce intervention, adjustment for confounding by indication is a serious challenge in this setting, especially when using a limited routine data source, and residual confounding from imperfect measures of severity[48] and bias from adjusting for co-variates that were not confounders[49] were likely. Quality also varied

For peer review only - http://bmjopen.bmj.com/site/about/guidelines.xhtml

widely. This is noteworthy considering that this was a relatively recent set of papers. In addition, comparison and synthesis has been limited by the mix in the papers of those who measure outcomes where PAs are an addition to a team (presenting difficulties in attributing the outcomes to PAs as opposed to any other increase in team capacity) and those where PAs substitute for other physicians where the contribution of PAs themselves is actually being measured. Although every paper reported the contribution of PAs in its speciality/subspecialty as overall positive, it is important that the following summary of the main findings of the review is considered in the context of the issues of method and methodological quality.

Results were spread across a number of outcomes, though those related to operational measures - waiting times or times taken for treatment, as well as patient satisfaction - were most prevalent. Outcomes reported when employing PAs in emergency medicine were varied. Operational performance results reported were decreased waiting time and reduced length of stay in the emergency department,[33] an increase in length of visit for those seen by PAs[32] and reduced charges.[32] Health care outcomes reported were no difference in 72-hour revisits to the ED[36] or wound infection rate,[39] and differences which were difficult to interpret, for example an increased prescription rate[34], or increase[37] or decrease in analgesia prescribing.[35] The messages are remarkably similar for trauma and orthopaedics. Operational measures highlighted no difference to [44] or reduced [39,40,42,43] waiting times in the emergency, operative and post-operative phases of care; released physician time[40,42] and reduced cost.[39] Here the evidence on health outcomes was mostly positive – increased adherence to treatment processes such as antibiotic administration[39], reduced post-operative complications[39], no difference in fracture malunion[41]and either no difference[44] or a reduction[43] in mortality. High patient satisfaction and staff acceptability, albeit with some caveats, were also reported.[40,42]

For peer review only

For peer review only - http://bmjopen.bmj.com/site/about/guidelines.xhtml

The two studies in internal (acute) medicine were of high quality and were among the few replacing physicians with PAs. Both found no differences in clinical outcomes between PAs and residents, or in length of stay, although lower costs were reported.[45,46] In mental health, the one study's qualitative evidence points also to acceptability of the role through team cohesion and improvements in whole system working.[47]

Summarising across the specialties we have reported five studies where PAs were an addition to the team.[33,39,42,43,47] In these more patients are reported to have been treated; waiting, ED and operating room times are said to have been shorter and mortality to be lower; however assessment of the contribution of PAs as opposed to any increase in team capacity is limited. Eight studies which compared outcomes of care by PAs and physicians either when one or the other was providing care or when PAs were substituting overall for physicians [32,35,36,38,40,44,45,46] presented mixed results: either no or a very small difference to length of stay, reduced resource used but at equal or reduced cost, some time savings to senior physicians, lower analgesia prescribing, no difference in wound infection rate, inpatient mortality or re-attendance, or in acceptability to staff and patients. In three of the studies we do not know if the PAs were additions or substitutions but two reported higher prescribing by PAs.[34,37] and one no difference in negative outcomes from fracture.[41]

Strengths and weaknesses

This review has systematically assessed the body of PA literature most immediately applicable to the current UK secondary care setting. We selected the five specialties in which PAs in the UK were mostly reported to be working[18] and therefore drew together the evidence of most relevance in that context and noted prominent gaps in evidence. However, this excluded evidence from other specialties. We excluded any studies including intensive

For peer review only - http://bmjopen.bmj.com/site/about/guidelines.xhtml

care data as this overlapped with acute medicine in many abstracts and we could not separately draw this out, and similarly we excluded studies with medical and surgical specialties combined. We note that this literature appeared to include a greater proportion of studies with stronger study designs, including prospective and randomised designs; in particular we have excluded the recent matched controlled large studies from the Netherlands in which several specialties – some within and some without our inclusion criteria - were studied.[50,51]

All of the included papers were from North America, with the majority from the USA, where health service organisation and the PA role may differ from that in other countries developing the PA role. In the USA PAs can prescribe and order ionising radiation, and are, as a body, more experienced than in countries more recently embracing this role.

We planned to carry out meta-analysis as appropriate to the literature included. The diversity of intervention as in initiation of PAs or change to PA practice being measured prevented this, as did identifying the effect of PAs when there were other simultaneous changes, even where a body of literature pertaining to a particular outcome measure, such as length of stay, was included. Although narrative review is more limited in its precision, in following a framework for this, we have aimed to provide a clear rationale for the synthesis and conclusions we draw from it.

Meaning of the study

This evidence is heavily weighted towards process times and patient satisfaction, with much less on health outcomes, although outcomes are crucial to assess safety of practice for all clinicians. Similar findings have been reported in a systematic review of new (non-medical)

For peer review only - http://bmjopen.bmj.com/site/about/guidelines.xhtml

roles in emergency medicine – reductions in waiting times in emergency departments, high level of patient satisfaction, confidence and acceptance of the roles. [52] Evidence also suggests that the perception of waiting times and satisfaction are correlated. [53]

Evidence from outside of the USA is very slim, as is evidence from multi-centre studies. The implications of this for policy can be seen in two ways.

Firstly, the limitations to evidence could be considered a cause for some concern, particularly in light of exponential growth in training numbers for PAs in England (alongside other UK countries),[54] government support for increased numbers (in primary care at least)[10] and for recent consultation on the introduction of statutory regulation for PAs, alongside judgment by employers and workforce planners of the role's value, alongside other medical associate professions.[55,56] Numbers of PAs are also rising rapidly in the US.[4] That said, the evidence presented is this review is positive and likely supportive of the direction of travel in policy. In addition, the case for PAs in the UK secondary care setting is made on the stability they might offer to medical teams and their broad knowledge in the face of hyper-specialisation[57] and recently-acquired knowledge – although not covered in this review due to its inclusion of PAs from across multiple specialties - suggests that PAs in England work in teams of multiple medical and other clinical staff grades[58] and that they are seen primarily as a resource where there are significant medical staffing issues.[59] High quality, multi-centre matched controlled substitution evidence from the Netherlands, [50,51] reassuringly also offers similar evidence to that included in our review regarding no difference in a large number of inpatient and post-discharge clinical outcomes, alongside an increase in patient satisfaction. The study found no difference in total healthcare costs or Quality Adjusted Life Years, despite lower personnel costs. The authors conclude that PA substitution appeared safe. The studies included in this review can be seen as complex interventions in complex systems and yet this has not been considered in the conclusions the

For peer review only - http://bmjopen.bmj.com/site/about/guidelines.xhtml

authors draw. Well-controlled studies are needed to fill in the gaps in our knowledge about the outcomes of PAs' contribution to the secondary care. More such evidence is required as well as further evaluation from a realist perspective – considering context, mechanisms and outcome - if PAs cannot be separated from service; measurement would utilise the principles of realist complex intervention science[60] or process evaluation to "Clearly describe the intervention and clarify causal assumptions (in relation to how it will be implemented, and the mechanisms through which it will produce change, in a specific context)."[61]

Conclusion

Modest research evidence exists on physician associates working in emergency medicine, trauma and orthopaedics. acute internal medicine; very limited evidence in mental health and none meeting our criteria in care of the elderly. The focus of the research is mainly on organisational and financial implications because increasing throughput of patients, whilst containing costs and without adversely affecting outcomes is fundamental to the rationale for the PA role. Evidence shows that use of PAs can achieve this objective. The PAs worked as additions as well as substitutes in complex systems where work is organised in teams which creates challenges for identifying cause and effect. Physician associate employment is also often part of wider service re-design or staffing changes in response to other changes, for example, availability of medical staff. The evidence here suggests that PAs can make a positive contribution to medical care and medical teams. Further research to the standard of more recent publications, is needed to elucidate the impact of PAs in different specialty areas, including comparators, and reporting on more than one setting, including countries in which the PA role is expanding rapidly. .

For peer review only

For peer review only - http://bmjopen.bmj.com/site/about/guidelines.xhtml

COMPETING INTERESTS

SdeL: University of Surrey runs a Physician Associate course.

- JP: chairs the UK and Ireland Board for Physician Associate Education and is director of the Physician Associate programme at the University of Birmingham.

FUNDING AND DISCLAIMER

This project was funded by the National Institute for Health Research Health Services and Delivery Research Programme (project number 14/19/26).

The views and opinions expressed herein are those of the authors and do not necessarily reflect those of the HS&DR Programme, NIHR, NHS or the Department of Health.

DATA SHARING STATEMENT

No further data are available

CONTRIBUTOR STATEMENT

MH led the design, execution and writing of this paper, under the direction of the study's PI VMD and supported by discussion with and written feedback from all co-authors'(CW, FP, HG, SdeL, JP, RG, JG, LN) on the design of the review and interpretation of findings. In addition, MH, CW, FP and VMD searched for literature and carried out data extraction and quality assessment. All authors (CW, FP, HG, SdeL, JP, RG, JG, LN, VMD) contributed intellectual content to the paper.

For peer review only - http://bmjopen.bmj.com/site/about/guidelines.xhtml

TABLES AND FIGURES:

Figure 1: 'Risk of bias' graph: review authors' judgements about each risk of bias item presented as percentages across all included studies

Figure 2: PRISMA flow chart

Supplementary file 1: Scoping review (Preliminary Medline search strategy – 24/11/2015) and MeSH (Medical Subject Headings) definition of search terms used

For peer review only

For peer review only - http://bmjopen.bmj.com/site/about/guidelines.xhtml

REFERENCES

1. World Health Organisation Global strategy on human resources for health: Workforce 2030. Draft (2015) Accessed last 12-09-2016 http://www.who.int/hrh/resources/pub_globstrathrh-2030/en/
2. World Health Organisation. Task shifting: rational redistribution of tasks among health workforce teams - global recommendations and guidelines. Geneva: World Health Organisation; 2008.
3. Mittman DE, Cawley JF, Fenn WH. Physician assistants in the United States. *BMJ* 2002;325:485-487.
4. NCCPA. 2016 Statistical Profile of Certified Physician Assistants an Annual report of the National Commission on Certification of Physician Assistants. 2017 NCCPA. Accessed January 25 2018 at https://prodcmsstoragesa.blob.core.windows.net/uploads/files/2016StatisticalProfileofCertifiedPhysicianAssistants.pdf
5. AAPA. What is a PA? Accessed March 22 2017 at https://www.aapa.org/what-is-a-pa/
6. Hooker RS, Hogan K, Leeker E. The globalization of the physician assistant profession. *J Physician Assist Educ* 2007;18:76–85.
7. Health Education England. HEE Business Plan 2015/16. 201515. Accessed 02-09-2016 https://hee.nhs.uk/sites/default/files/documents/HEE-Business-Plan-2015-16.pdf

For peer review only - http://bmjopen.bmj.com/site/about/guidelines.xhtml

8. Healthforce Ontario 2009 Ontario's Physician Assistant Initiative: An Overview. Accessed on 13-09-2016 at http://www.healthforceontario.ca/en/M4/Ontario%27s_Physician_Assistant_Initiative

9. Synergia Limited. 2015. Phase II of the Physician Assistant Demonstrations Evaluation Report: Report for Health Workforce New Zealand. Wellington: Ministry of Health. Accessed last 2-9-2016 at http://www.health.govt.nz/publication/phase-ii-physician-assistant-demonstrations-evaluation-report

10. Department of Health and The Rt Hon Jeremy Hunt MP. New Deal for General Practice, https://www.gov.uk/government/speeches/new-deal-for-general-practice (2015, accessed 25th January 2018).

11. Drennan V, Halter M, Brearley S et al. Investigating the contribution of physician assistants to primary care in England: a mixed-methods study. *Health Services and Delivery Research* 2014;2.

12. Velde FVD, Windt WVD. Alumni van de masteropleiding Physician Assistant. Utrecht: Kiwa Prismant; 2013. Cited in Timmermans MJ, van Vught AJ, Wensing M, Laurant MG. The effectiveness of substitution of hospital ward care from medical doctors to physician assistants: a study protocol. *BMC Health Serv Res* 2014;14:43. doi: 10.1186/1472-6963-14-43.

13. Ritsema T. Faculty of Physician Associates Census results 2017. Royal College of Physicians Faculty of Physician Associates. Accessed 25th January 2018 at http://www.fparcp.co.uk/about-fpa/fpa-census

For peer review only

For peer review only - http://bmjopen.bmj.com/site/about/guidelines.xhtml

14. Hooker RS, Everett CM. The contributions of physician assistants in primary care systems. *Health Soc Care Community* 2012;20–31.

15. Halter M, Drennan V, Chattopadhyay K, et al. The contribution of physician assistants in primary care: a systematic review. *BMC Health Serv Res* 2013;18:223. doi: 10.1186/1472-6963-13-223.

16. Doan Q, Sabhaney V, Kissoon N, et al. A systematic review: the role and impact of the physician assistant in the emergency department. *Emerg Med Australas* 2011;23:7-15.

17. Johal J, Dodd A. Physician extenders on surgical services: a systematic review. Can J Surg. 2017;;60:172-178.

18. Ritsema TS. Faculty of Physician Associates Census Results 2016. Accessed 10 September 2017 at http://www.fparcp.co.uk/about-fpa/fpa-census

19. Merkle F, Ritsema TS, Bauer S, Kuilman L. The physician assistant: Shifting the Paradigm of European medical practice? *HSR Proc Intensive Care Cardiovasc Anesth* 2011;3:255-262.

20. Moher D, Liberati A, Tetzlaff J et al. The PRISMA Group. Preferred reporting items for systematic reviews and meta-analyses: the PRISMA statement. *J Clin Epidemiol* 2009;62:1006–12.

21. Halter M, Wheeler C, Drennan V, et al. Evidence of impact of physician associates in secondary care: a systematic mixed studies review. PROSPERO 2016:CRD42016032895. Accessed on 10 September 2017 http://www.crd.york.ac.uk/PROSPERO/display_record.asp?ID=CRD42016032895.

For peer review only - http://bmjopen.bmj.com/site/about/guidelines.xhtml

22. Greenhalgh T, Peacock R. Effectiveness and efficiency of search methods in systematic reviews of complex evidence: audit of primary sources. *BMJ* 2005; 331:1064–1065

23. Department of Health. The Competence and Curriculum Framework for the Physician Assistant. London: Department of Health 2006.

24. Maxwell R. Dimensions of quality revisited: from thought to action. *Qual Health Care* 1992:1:171-177.

25. International Monetary Fund. Table B1. Advanced Economies: Unemployment, Employment, and Real GDP per Capita. https://www.imf.org/external/pubs/ft/weo/2015/01/pdf/tblpartb.pdf Accessed on 14 December 2015.

26. Kmet LM, Lee RC, Cook LS. Standard quality assessment criteria for evaluating primary research papers from a variety of fields. *Alberta Herit Found Med Res* 2004;13:1–11. http://files.deslibris.ca/cppc/200/200548.pdf Accessed November 24 2015.

27. Pace R, Pluye P, Bartlett G, et al. Testing the reliability and efficiency of the pilot Mixed Methods Appraisal Tool (MMAT) for systematic mixed studies review. Int. J. Nurs. Stud. (2011), doi:10.1016/j.ijnurstu.2011.07.002

28. Centre for Reviews and Dissemination, University of York. Systematic Reviews. CRD's guidance for undertaking reviews in healthcare. 2009.

29. Thomas J, Harden A: Methods for the thematic synthesis of qualitative research in systematic reviews. *BMC Med Res Meth* 2008;8:45.

30. Popay J, Roberts H, Sowden A, et al. Guidance on the conduct of narrative synthesis in systematic reviews. ESRC Research Methods Programme. 2006.

For peer review only - http://bmjopen.bmj.com/site/about/guidelines.xhtml

31. Noyes J & Lewin S. Chapter 6: Supplemental Guidance on Selecting a Method of Qualitative Evidence Synthesis, and Integrating Qualitative Evidence with Cochrane Intervention Reviews. In: Noyes J, Booth A, Hannes K, et al (editors), Supplementary Guidance for Inclusion of Qualitative Research in Cochrane Systematic Reviews of Interventions. Version 1 (updated August 2011). Cochrane Collaboration Qualitative Methods Group, 2011. Available from URL http://cqrmg.cochrane.org/supplemental-handbook-guidance Last accessed January 2015.

32. Arnopolin SL, Smithline HA. Patient care by physician assistants and by physicians in an emergency department. *JAAPA* 2000;13:39-40, 49-50, 53-4 passim.

33. Ducharme J, Alder RJ, Pelletier C, et al. The impact on patient flow after the integration of nurse practitioners and physician assistants in 6 Ontario emergency departments. CJEM. 2009;11:455-461.

34. Hooker RS, Cipher DJ, Cawley JF, et al. Emergency medicine services: Interprofessional care trends. J Interprof Care 2009;22: 167-178.

35. Kozlowski MJ, Wiater JG, Pasqual RG, et al. Painful discrimination: The differential use of analgesia in isolated lower limb injuries. Am J Emerg Med.2002;20:502-5.

36. Pavlick D, Sacchetti A, Seymour A, et al. Physician assistant management of pediatric patients in a general community emergency department: A real-world analysis. *Pediatr Emerg Care* 2017;33:26-30.

37. Ritsema TS, Kelen GD, Pronovost PJ, et al. The National Trend in Quality of Emergency Department Pain Management for Long Bone Fractures. *Acad Emerg Med* 2007;14:163-9.

38. Singer AJ, Hollander JE, Cassara G, et al. Level of training, wound care practices, and infection rates. *Am J Emerg Med* 1995;13:265-8.

For peer review only - http://bmjopen.bmj.com/site/about/guidelines.xhtml

39. Althausen PL, Shannon S, Owens B, et al. Impact of hospital-employed physician assistants on a level II community-based orthopaedic trauma system. *J Orthop Trauma.* 2013;27:e87-91. doi: 10.1097/BOT.0b013e3182647f29.

40. Bohm ER, Dunbar M, Pitman D, et al. Experience with physician assistants in a Canadian arthroplasty program. *Can J Sur.* 2010;53:103-8.

41. Garrison S, Eismann EA, Cornwall R. Does using PAs in the closed treatment of pediatric forearm fractures increase malunion risk? *JAAPA* 2017;30:41-45.

42. Hepp SL, Suter E, Nagy D, et al. Utilizing the physician assistant role: case study in an upper-extremity orthopedic surgical program. *Can J Surg* 60:115-121.

43. Mains C Scarborough K, Baror R, et al. Staff commitment to trauma care improves mortality and length of stay at a level i trauma center. *J Trauma* 2009;66:1315-20. doi: 10.1097/TA.0b013e31819d96d8.

44. Oswanski MF, Sharma OP, Raj SS. Comparative review of use of physician assistants in a level I trauma center. *Am Surg* 2004;Mar;70:272-9.

45. Capstack TM, Seguija C, Vollono LM, et al. A comparison of conventional and expanded physician assistant hospitalist staffing models at a community hospital. *J Clin Outcomes.* 2016 23: 455-461.

46. Van Rhee J, Ritchie J, Eward A. Resource use by physician assistant services versus teaching services *JAAPA* 2002;15:33-8,40,42.cheon

47. McCutchen B, Patel S, Copeland D. Expanding the role of PAs in the treatment of severe and persistent mental illness. *JAAPA* 2017;30:36-37.

48. Greenland S, Robins JM, Pearl J. Confounding and collapsibility in causal inference. *Statistical Science* 14:29-46. http://www.biostat.harvard.edu/robins/publications/Confounding_and_collapsibility.pdf] Accessed March 30th 2017.

For peer review only

For peer review only - http://bmjopen.bmj.com/site/about/guidelines.xhtml

49. Greenland S, Pearl J, Robins JM. Causal diagrams for epidemiological research. *Epidemiology* 1999;10:37-48. http://www.biostat.harvard.edu/robins/publications/causaldia.pdf] Accessed March 30th 2017.

50. Timmermans MJC, van Vught AJAH, Peters YAS, et al. The impact of the implementation of physician assistants in inpatient care: A multicenter matched-controlled study. PLoS ONE 2017;12:e0178212..

51. Timmermans MJC, van den Brink GT, van Vught AJAH, et al. The involvement of physician assistants in inpatient care in hospitals in the Netherlands: a cost-effectiveness analysis. *BMJ Open*. 2017;7:e016405. doi: 10.1136/bmjopen-2017-016405.

52. Hoskins R. Evaluating new roles within emergency care: a literature review. *Int Emerg Nurs* 2011;19):125-40. doi: 10.1016/j.ienj.2010.09.003.

53. Saxon K, London K, Bacharouch A, et al. Patients' Perceptions of Waiting Times and the Effect on Patient Satisfaction in the Emergency Department. *Ann Emer Med* 2013;62,4 Supplement:S82

54. Health Education England. Medical Associate Professionals. https://hee.nhs.uk/printpdf/our-work/developing-our-workforce/medical-associate-professions. Accessed 7 February 2017.

55. Department of Health and Social Care. The regulation of medical associate professions in the UK Closed consultation. Accessed 25th January 2018 at https://www.gov.uk/government/consultations/regulating-medical-associate-professions-in-the-uk

56. Department of Health The regulation of medical associate professions in the UK Consultation document, 2017. Accessed 25th January 2018 at https://www.gov.uk/government/uploads/system/uploads/attachment_data/file/650554/The_regulation_of_MAPs_in_the_UK.pdf

For peer review only - http://bmjopen.bmj.com/site/about/guidelines.xhtml

57. Ross N, Parle J, Begg P et al. The case for the physician assistant. *Clinical Medicine* 2012;12:200–6.

58. Wheeler C, Halter M, Drennan VM, et al. Physician associates working in secondary care teams in England: Interprofessional implications from a national survey. *J Interprof Care* 2017;6:1-3. doi: 10.1080/13561820.2017.1341390.

59. Halter M, Wheeler C, Drennan V, et al. Physician Associates in England's hospitals: a survey of medical directors exploring current usage and supporting/inhibiting factors to their recruitment. *Clin Med* 2017;17:126-131. doi: 10.7861/clinmedicine.17-2-126.

60. Fletcher A, Jamal F, Moore G, et al. Realist complex intervention science: Applying realist principles across all phases of the Medical Research Council framework for developing and evaluating complex interventions. *Evaluation* 2016;22:286-303

61. Moore GF, Audrey S, Barker M, et al. Process evaluation of complex interventions: Medical Research Council guidance. *BMJ* 2015;350:p.h1258.

For peer review only

For peer review only - http://bmjopen.bmj.com/site/about/guidelines.xhtml

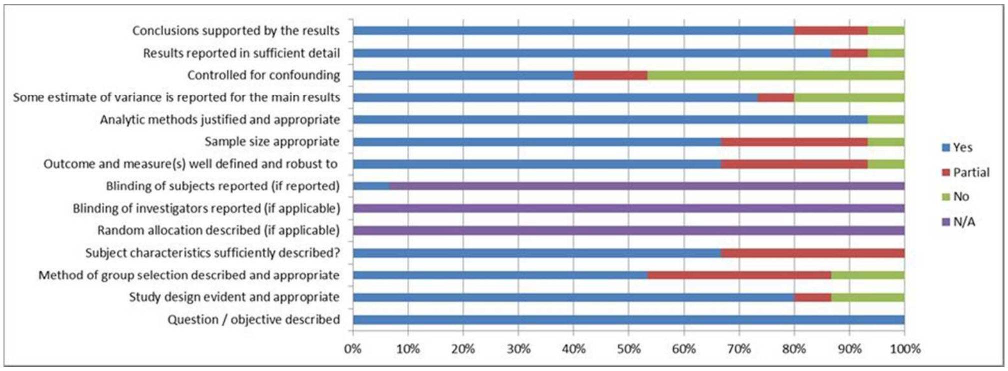

Study selection

81x29mm (300 x 300 DPI)

For peer review only - http://bmjopen.bmj.com/site/about/guidelines.xhtml

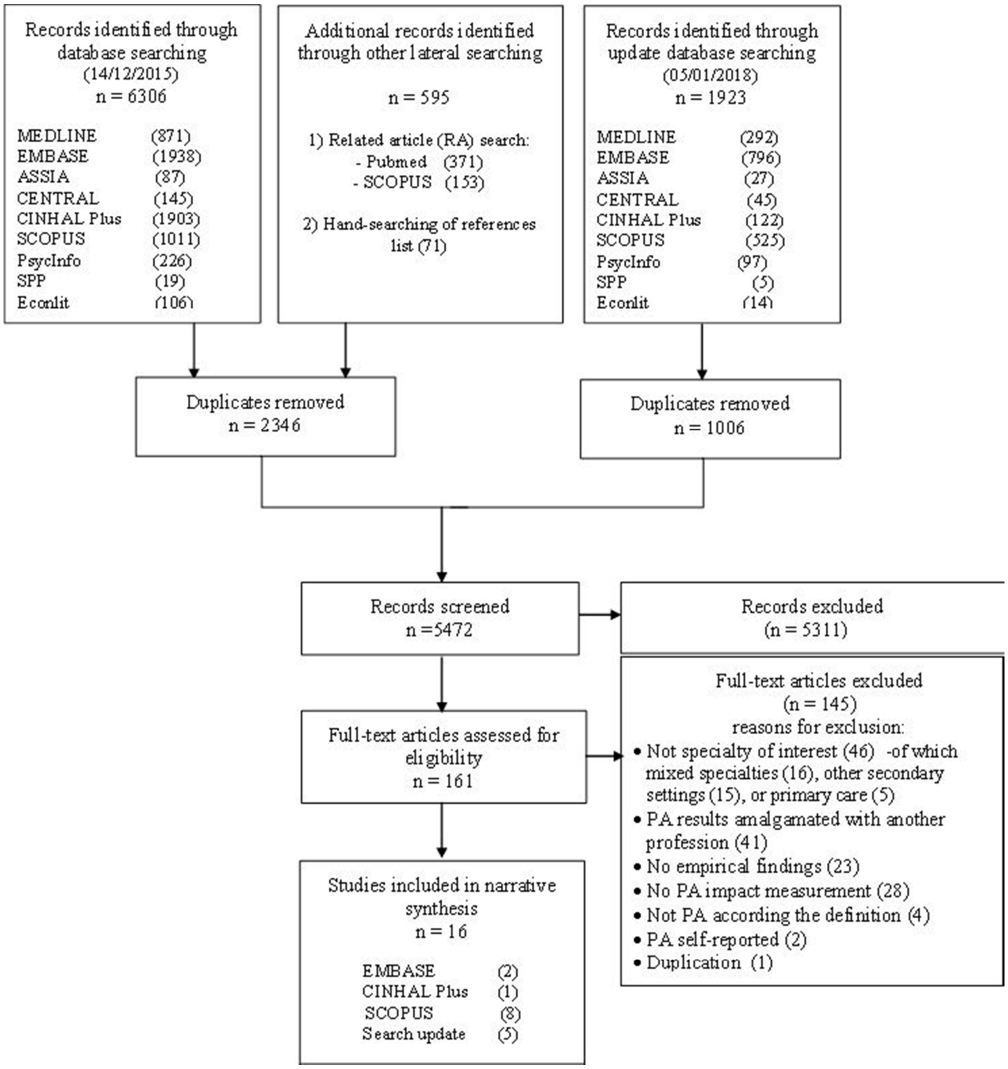

Quality assessment

62x66mm (300 x 300 DPI)

SUPPLEMENTARY FILE 1: Scoping review (Preliminary Medline search strategy – 24/11/2015)

#	Concept		Search Terms	Results
1	Physician Associates		exp Physician Assistants/	2410
2			exp Pediatric Assistants/	26
3			Physician Assistant$.tw.	1498
4			Feldsher$.tw.	17
5			Clinical Officer$.tw.	135
6			Paramedical Practitioner&.tw.	0
7			Medical Assistant$.tw.	324
8			Allied Health Personnel.tw.	48
9			physician associate$.tw.	37
10			(mid level adj3 provider$).tw.	124
11			((assistant* or technician* or officer* or associate$) adj2 (physician$ or surgical or clinical$ or practitioner$ or medical$ or provider$)).tw.	24985
12	Secondary Care	Emergency Medicine	exp Emergency Medicine/ and (speciali?ed or specialty or hospital$ or secondary or care or medicine).tw.	4983
13			((accident and emergency) or A&E department or emergency department or casualty or emergency Medicine).tw.	47842
14			(emergency adj3 (medic* or servic* or ward* or department)).tw.	54262
15			(exp critical care/ or exp intensive care/) and (speciali?ed or specialty or hospital$ or secondary or care or medicine).tw.	23791
16			((intensive adj3 care) and (speciali?ed or specialty or hospital$ or secondary or care or medicine)).tw.	71552
17		Acute Medicine	exp Internal Medicine/ and (speciali?ed or specialty or hospital$ or secondary or care or medicine).tw.	16968
18			(internal medicine and (speciali?ed or specialty or hospital$ or secondary or care or medicine)).tw.	10752
19			(Acute Medicine or acute internal medicine or acute medical unit$ or medical assessment unit$ or acute ward$).tw.	690
20		Trauma or Orthopaedics	(exp Orthopedics/ or exp Traumatology/) and (speciali?ed or specialty or hospital$ or secondary or care or medicine).tw.	3015
21			((Trauma or Orthop?dic$) adj3 (speciali?ed or specialty or hospital$ or secondary or care or medicine)).tw.	7280
22			(Orthop?dic surgery or trauma surgery).tw.	4466
23			((bone$ or joint$ or ligament$ or tendon$ or muscle$ or nerve$) adj3 (operation$ or surgery or replacement$)).tw.	13668
24		Care of the Elderly	(exp geriatrics/ or Aging/ or exp Aged/ or older people.mp. or exp Frail Elderly/) and (speciali?ed or specialty or hospital$ or secondary or care).tw.	361294
25			((Older adult or Aged or elderly or geriatric* or older people* or ag?ng) adj3 (speciali?ed or specialty or hospital$ or secondary or care or medicine)).tw.	15561

For peer review only

26			or/12-25	508965
27			exp Primary Health Care/ or exp preventive medicine/ or exp physicians, Primary Care/	75166
28			(primary care or primary healthcare or primary health care or primary health service$).tw.	68593
29			27 or 28	111510
30		**Primary care**	exp Family Practice/ or exp Physicians, Family/ or exp General Practitioners/ or exp General Practice/	47498
31			(family practice$ or family practitioner$ or family physician$ family medicine$ or General practice$ or General practitioner$ or GPs).tw.	47129
32			30 or 31	72038
33			29 not 32	91680
34		**Outpatient and inpatient care**	(exp Outpatients/ or Outpatient Clinics, Hospital/ or ambulatory care/) and (speciali?ed or specialty or hospital$ or secondary or care or medicine).tw.	18427
35			(exp Inpatients/ or Hospitalization/) and (speciali?ed or specialty or hospital$ or secondary or care or medicine).tw.	49797
36			(ambulatory care or ambulatory emergency care).tw.	3948
37			((outpatient$ or out-patient$) adj3 (speciali?ed or specialty or hospital$ or secondary or care or medicine)).tw.	11455
38			((inpatient$ or in-patient$) adj3 (speciali?ed or specialty or hospital$ or secondary or care or medicine)).tw.	24157
39	**Impact**		Treatment Outcome/ or "Outcome and Process Assessment (Health Care)"/ or "Outcome Assessment (Health Care)"/ or Medical Audit/ or Program Evaluation/	769470
40			exp Patient Readmission/ or exp Length of Stay/ or exp Clinical Audit/ or exp Medical Audit/	68267
41			Health Planning/ and (organi?ation* or system* or hospital* or Physician* or workforce or staff or professional*).tw.	2686
42			Efficiency, Organizational/ and (organi?ation* or system* or hospital* or Physician* or workforce or staff or professional*).tw.	8952
43			Resource Allocation/ and (organi?ation* or system* or hospital* or Physician* or workforce or staff or professional*).tw.	1377
44			Health Personnel/ and (organi?ation* or system* or hospital* or Physician* or workforce or staff or professional*).tw.	11958
45			Health Manpower/ and (organi?ation* or system* or hospital* or Physician* or workforce or staff or professional*).tw.	2123
46			Medical Staff/ and (organi?ation* or system* or hospital* or Physician* or workforce or staff or professional*).tw.	899
47			Delivery of Health Care/ and (productivity or efficiency or performance or guideline* or quality).tw.	8411
48			((equity or difference$ disparit$ or inequalit$ or inequit$) adj5 (experience$ or perception$ or view$ or rates or rating or review or audit or impact or influence or effect or outcome or performance or quality)).tw.	2048
49			((Acceptability or compassion or dignity or satisfaction or dissatisfaction) adj5 (experience$ or perception$ or view$ or rates or rating or review or audit or impact or influence or effect or outcome or performance or quality)).tw.	16604

For peer review only

50		((Efficiency or productivity or economic$ or benefit) adj5 (experience$ or perception$ or view$ or rates or rating or review or audit or impact or influence or effect or outcome or performance or quality)).tw.	34565
51		((Effectiveness or efficacy or effectivity or capability) adj5 (experience$ or perception$ or view$ or rates or rating or review or audit or impact or influence or effect or outcome or performance or quality)).tw.	35758
52		((Effectiveness or efficacy or effectivity or capability) adj5 (experience$ or perception$ or view$ or rates or rating or review or audit or impact or influence or effect or outcome or performance or quality)).tw.	35758
53		((Access$ or responsiveness or timely or timeliness) adj5 (experience$ or perception$ or view$ or rates or rating or review or audit or impact or influence or effect or outcome or performance or quality)).tw.	16251
54		((Appropriate$ or relevance or relevant) adj5 (experience$ or perception$ or view$ or rates or rating or review or audit or impact or influence or effect or outcome or performance or quality)).tw.	32405
55		((Cost$ or afford$ value for money or financ$) adj5 (experience$ or perception$ or view$ or rates or rating or review or audit or impact or influence or effect or outcome or performance or quality)).tw.	33373
56	**Impact in Secondary Care of Physician Associates**	or/1-11	26515
57		26 or 33 or 34 or 35 or 36 or 37 or 38	621770
58		or/39-55	959419
59		56 and 57 and 58	1575
60		limit 59 to (english language and last 20 years)	1513

For peer review only

SUPPLEMENTARY FILE 1 continued: MeSH --Medical Subject Headings definition of search terms used

(alphabetical [US spellings])

http://www.ncbi.nlm.nih.gov/mesh

Aged: A person 65 through 79 years of age. For a person older than 79 years, AGED, 80 AND OVER is available. Year introduced: 1966. By exploding this term, we do include MeSH terms found below it in the MeSH hierarchy as follows: Aged, 80 and over; Frail Elderly.

Aging: The gradual irreversible changes in structure and function of an organism that occur as a result of the passage of time. By exploding this term, we do include MeSH terms found below it in the MeSH hierarchy as follows: Longevity.

Ambulatory care: Health care services provided to patients on an ambulatory basis, rather than by admission to a hospital or other health care facility. The services may be a part of a hospital, augmenting its inpatient services, or may be provided at a free-standing facility. Year introduced: 1968(1966)

Behavioral Disciplines and Activities: The specialties in psychiatry and psychology, their diagnostic techniques and tests, their therapeutic methods, and psychiatric and psychological services. Year introduced: 1998

Clinical Audit: A detailed review and evaluation of selected clinical records by qualified professional personnel to improve the quality of patient care and outcomes. The clinical audit was formally introduced in 1993 into the United Kingdom's National Health Service. Year introduced: 2008

Critical Care: Health care provided to a critically ill patient during a medical emergency or crisis. Year introduced: 1975

Emergency medicine: The branch of medicine concerned with the evaluation and initial treatment of urgent and emergent medical problems, such as those caused by accidents, trauma, sudden illness, poisoning, or disasters. Emergency medical care can be provided at the hospital or at sites outside the medical facility.

Family Practice: A medical specialty concerned with the provision of continuing, comprehensive primary health care for the entire family.

For peer review only - http://bmjopen.bmj.com/site/about/guidelines.xhtml

Frail Elderly: Older adults or aged individuals who are lacking in general strength and are unusually susceptible to disease or to other infirmity. Year introduced: 1991

General Practice: Patient-based medical care provided across age and gender or specialty boundaries. Year introduced: 2011

General Practitioners: Physicians whose practice is not restricted to a specific field of medicine

Geriatrics: The branch of medicine concerned with the physiological and pathological aspects of the aged, including the clinical problems of senescence and senility.

Hospitalization: The confinement of a patient in a hospital.

Inpatients: Persons admitted to health facilities which provide board and room, for the purpose of observation, care, diagnosis or treatment.

Intensive care: Advanced and highly specialized care provided to medical or surgical patients whose conditions are life-threatening and require comprehensive care and constant monitoring. It is usually administered in specially equipped units of a health care facility. Year introduced: 1992

Internal Medicine: A medical specialty concerned with the diagnosis and treatment of diseases of the internal organ systems of adults. By exploding this term, we do include MeSH terms found below it in the MeSH hierarchy as follows: Cardiology; Cardiac electrophysiology; Endocrinology; Gastroenterology; Hematology; Transfusion Medicine; Infectious Disease Medicine; Medical Oncology Radiation; Oncology; Nephrology; Pulmonary Medicine; Rheumatology; Sleep Medicine Specialty.

Medical Audit: A detailed review and evaluation of selected clinical records by qualified professional personnel for evaluating quality of medical care. Year introduced: 1968

Mental Disorders: Psychiatric illness or diseases manifested by breakdowns in the adaptational process expressed primarily as abnormalities of thought, feeling, and behavior producing either distress or impairment of function. Year introduced: use pre-explosion 1974-1997

Mental Health Services: Organized services to provide **mental health** care. Year introduced: 1967
Mental Health: The state wherein the person is well adjusted. Year introduced: 1967

Orthopedics: A surgical specialty which utilizes medical, surgical, and physical methods to treat and correct deformities, diseases, and injuries to the skeletal system, its articulations, and associated structures.

For peer review only

PA-SCer systematic review narrative synthesis: first draft T&O and internal med 160806

Outcome and Process Assessment (Health Care): Evaluation procedures that focus on both the outcome or status (OUTCOMES ASSESSMENT) of the patient at the end of an episode of care - presence of symptoms, level of activity, and mortality; and the process (ASSESSMENT, PROCESS) - what is done for the patient diagnostically and therapeutically. Year introduced: 1979. By exploding this term, we do include MeSH terms found below it in the MeSH hierarchy as follows: Outcome Assessment (Health Care); Patient Outcome Assessment; Treatment Outcome; Process Assessment (Health Care)

Outcome Assessment (Health Care): Research aimed at assessing the quality and effectiveness of health care as measured by the attainment of a specified end result or outcome. Measures include parameters such as improved health, lowered morbidity or mortality, and improvement of abnormal states (such as elevated blood pressure). Year introduced: 1992

Outpatient Clinics, Hospital: Organized services in a hospital which provide medical care on an outpatient basis. Year introduced: 1978

Outpatients: Persons who receive ambulatory care at an outpatient department or clinic without room and board being provided. Year introduced: 1991(1980)

Pediatric Assistants: Persons academically trained to provide medical care, under the supervision of a physician, to infants and children. Year introduced: 1991(1975)

Physician Assistants: Health professionals who practice medicine as members of a team with their supervising physicians. They deliver a broad range of medical and surgical services to diverse populations in rural and urban settings. Duties may include physical exams, diagnosis and treatment of disease, interpretation of tests, assist in surgery, and prescribe medications. (from http://www.aapa.orglabout-pas accessed 2114/2011) Year introduced: 1995

Physicians, Family: Those physicians who have completed the education requirements specified by the American Academy of Family Physicians. Year introduced: 1974(1972)

Physicians, Primary Care: Providers of initial care for patients. These PHYSICIANS refer patients when appropriate for secondary or specialist care. Year introduced: 2011

Preventive medicine: A medical specialty primarily concerned with prevention of disease (PRIMARY PREVENTION) and the promotion and preservation of health in the individual. By exploding this term, we do include MeSH terms found below it in the MeSH hierarchy as follows: Environmental Medicine; Occupational Medicine; Preventive Psychiatry.

For peer review only

PA-SCer systematic review narrative synthesis: first draft T&O and internal med 160806

Primary Health Care: Care which provides integrated, accessible health care services by clinicians who are accountable for addressing a large majority of personal health care needs, developing a sustained partnership with patients, and practicing in the context of family and community. (JAMA 1995;273(3):192) Year introduced: 1974(1972).

Program Evaluation: Studies designed to assess the efficacy of programs. They may include the evaluation of cost-effectiveness, the extent to which objectives are met, or impact. Year introduced: 1989. By exploding this term, we do include MeSH terms found below it in the MeSH hierarchy as follows: benchmarking.

Psychiatry: The medical science that deals with the origin, diagnosis, prevention, and treatment of mental disorders.

Traumatology: The medical specialty which deals with wounds and injuries as well as resulting disability and disorders from physical traumas.

Treatment Outcome: Evaluation undertaken to assess the results or consequences of management and procedures used in combating disease in order to determine the efficacy, effectiveness, safety, and practicability of these interventions in individual cases or series. Year introduced: 1992

For peer review only

SUPPLEMENTARY FILE 2: DEFINITIONS used in this review

As this review question contained broad terms, these were defined at the outset, as follows:

- Physician Associates: trained in a medical model to work in all settings and undertake physical examinations, investigations, diagnosis, treatment, and prescribe within their scope of practice as agreed with their supervising doctor.[1,2] Physician Associates are sometimes described within the term 'mid-level providers' in developed economies: '…..the term mid-level practitioner means an individual practitioner, other than a physician, dentist, veterinarian, or podiatrist, who is licensed, registered, or otherwise permitted by the United States or the jurisdiction in which he/she practices, to dispense a controlled substance in the course of professional practice. Examples of mid-level practitioners include, but are not limited to, health-care providers such as nurse practitioners, nurse midwives, nurse anaesthetists, clinical nurse specialists and physician assistants who are authorized to dispense controlled substances by the state in which they practice.' [3] While this term is contested as an appropriate umbrella term due to its hierarchical connotations [4,5] and international variation in usage,[6] it appears in the literature regarding Physician Associates.
- Impact: using the broad headings of the components of quality as suggested by Maxwell (1992),[7] augmenting that of Donabedian,[8] that is, effectiveness, efficiency, acceptability, access, equity and relevance; further consolidated in the aspects of quality set out in the NHS Next stage Review (2008)[9]: patient safety, patient experience and effectiveness of care.
- Specialties most frequently employing PAs in England:
    - acute medicine
      'Acute medicine is the part of general (internal) medicine concerned with the immediate and early specialist management of adult patients who present to, or from within, hospitals as urgencies or emergencies'.[10]
    - care of the elderly
      '…geriatric medicine is mainly concerned with people over the age of 75, although many 'geriatric' patients are much older. However, geriatric medicine in the UK is broadly from the age of 65 onwards. Frail older people are those with multiple diseases, that often includes dementia, with reduced functional reserve who tend to present to hospital with 'geriatric syndromes' such as falls, confusion and immobility.'[11]
    - emergency medicine
      'Emergency medicine is a field of practice based on the knowledge and skills required for the prevention, diagnosis and management of acute and urgent aspects of illness and injury affecting patients of all age groups with a full spectrum of episodic undifferentiated physical and behavioural disorders; it further encompasses an understanding of the development of

For peer review only

prehospital and in hospital emergency medical systems and the skills necessary for this development.' [12]

– mental health /psychiatry

'Mental health problems can take many forms including depression, schizophrenia, eating disorders, anxieties, phobias, drug and alcohol abuse, post-traumatic stress disorder, and dementia.'[13] Psychiatry includes the sub specialties of child and adolescent, forensic, general adult, old age, psychotherapy and psychiatry of learning disabilities. [14]

- trauma and orthopaedics

Trauma and orthopaedics is an area of surgery concerned with injuries and conditions that affect the musculoskeletal system (the bones, joints, ligaments, tendons, muscles and nerves).[15]

For peer review only

References

1. Department of Health. The Competence and Curriculum Framework for the Physician Assistant. London: Department of Health 2006.
2. Physician Assistant Managed Voluntary Register The Revised Competence and Curriculum Framework for the Physician Assistant. 2012 Accessed at http://www.ukapa.co.uk/files/CCF-27-03-12-for-PAMVR.pdf.
3. United States Department of Justice. (n.d.). Mid-level practitioners authorization by state. Retrieved from http://www.deadiversion.usdoj.gov/drugreg/practioners/index.html
4. Bishop CS. Advanced Practitioners Are Not Mid-Level Providers. Vol 3 No 5 Sep/Oct 2012 287-8. J Adv Pract Oncol AdvancedPractitioner.com http://www.advancedpractitioner.com/media/152572/287.pdf
5. Pappas MD. Stop calling nurse practitioners mid-level providers. 2014 Kevin MD.com. http://www.kevinmd.com/blog/2014/07/stop-calling-nurse-practitioners-mid-level-providers.html. Accessed 24 November 2015.
6. Brown A, Cornetto G, Cumbi A, de Pinho H, Kamwendo F, Lehmann U et al. Mid-level health providers: a promising resource. Rev Peru Med Exp Salud Publica. 2011; 28(2): 308-15.
7. Maxwell R. Dimensions of quality revisited: from thought to action. Quality in Health Care 1992:1:171-177.
8. Donabedian A. The quality of care. How can it be assessed? JAMA 1988;260:1743–8. http://dx.doi.org/10.1001/jama.1988.03410120089033

9. Department of Health. High Quality Care For All NHS Next Stage Review Final Report. 2008. https://www.gov.uk/government/uploads/system/uploads/attachment_data/file/228836/7432.pdf Accessed 10 December 2015.
10. Royal College of Physicians. *Acute medical care; the right person, in the right setting—first time.* Report of a working party. London: RCP, 2007.
11. Conroy S, Cooper N. Executive summary. British Geriatrics Society, 2012. http://www.bgs.org.uk/index.php/topresources/publicationfind/goodpractice/44-gpgacutecare%20 Accessed 24 Nov 2015.
12. International Federation for Emergency Medicine. About IFEM IFEM Definition of Emergency Medicine. 2008. http://www.ifem.cc/About_IFEM.aspx Accessed Nov 24 2015.
13. Royal College of Psychiatrists. Glossary of Terms Professionals involved in the care of people with mental health problems. 2015. http://www.rcpsych.ac.uk/healthadvice/moreinformation/definitions/professionalsinpsychiatry.aspx. Accessed 10 December 2015.
14. Royal College of Psychiatrists. A Career in Psychiatry. 2015. http://www.rcpsych.ac.uk/discoverpsychiatry/acareerinpsychiatry.aspx Accessed 10 December 2015.
15. NHS Choices. Trauma and Orthopaedics (Orthopeadic Surgery) 2015. http://www.nhs.uk/Conditions/orthopaedics/Pages/Introduction.aspx

For peer review only

PRISMA 2009 Checklist

Section/topic	#	Checklist item	Reported on page #
**TITLE**			
Title	1	Identify the report as a systematic review, meta-analysis, or both.	1
**ABSTRACT**			
Structured summary	2	Provide a structured summary including, as applicable: background; objectives; data sources; study eligibility criteria, participants, and interventions; study appraisal and synthesis methods; results; limitations; conclusions and implications of key findings; systematic review registration number.	4-5
**INTRODUCTION**			
Rationale	3	Describe the rationale for the review in the context of what is already known.	6-7
Objectives	4	Provide an explicit statement of questions being addressed with reference to participants, interventions, comparisons, outcomes, and study design (PICOS).	9
**METHODS**			
Protocol and registration	5	Indicate if a review protocol exists, if and where it can be accessed (e.g., Web address), and, if available, provide registration information including registration number.	8 + reference
Eligibility criteria	6	Specify study characteristics (e.g., PICOS, length of follow-up) and report characteristics (e.g., years considered, language, publication status) used as criteria for eligibility, giving rationale.	9
Information sources	7	Describe all information sources (e.g., databases with dates of coverage, contact with study authors to identify additional studies) in the search and date last searched.	8
Search	8	Present full electronic search strategy for at least one database, including any limits used, such that it could be repeated.	Supplementary file
Study selection	9	State the process for selecting studies (i.e., screening, eligibility, included in systematic review, and, if applicable, included in the meta-analysis).	8-10
Data collection process	10	Describe method of data extraction from reports (e.g., piloted forms, independently, in duplicate) and any processes for obtaining and confirming data from investigators.	10
Data items	11	List and define all variables for which data were sought (e.g., PICOS, funding sources) and any assumptions and simplifications made.	10
Risk of bias in individual studies	12	Describe methods used for assessing risk of bias of individual studies (including specification of whether this was done at the study or outcome level), and how this information is to be used in any data synthesis.	10
Summary measures	13	State the principal summary measures (e.g., risk ratio, difference in means).	10-11
Synthesis of results	14	Describe the methods of handling data and combining results of studies, if done, including measures of consistency (e.g., I^2) for each meta-analysis.	10-11

For peer review only

PRISMA 2009 Checklist

Section/topic	#	Checklist item	Reported on page #
Risk of bias across studies	15	Specify any assessment of risk of bias that may affect the cumulative evidence (e.g., publication bias, selective reporting within studies).	10
Additional analyses	16	Describe methods of additional analyses (e.g., sensitivity or subgroup analyses, meta-regression), if done, indicating which were pre-specified.	n/a
**RESULTS**			
Study selection	17	Give numbers of studies screened, assessed for eligibility, and included in the review, with reasons for exclusions at each stage, ideally with a flow diagram.	11+figure1
Study characteristics	18	For each study, present characteristics for which data were extracted (e.g., study size, PICOS, follow-up period) and provide the citations.	Table 1
Risk of bias within studies	19	Present data on risk of bias of each study and, if available, any outcome level assessment (see item 12).	Table 2 + Figure2
Results of individual studies	20	For all outcomes considered (benefits or harms), present, for each study: (a) simple summary data for each intervention group (b) effect estimates and confidence intervals, ideally with a forest plot.	Table 1 + 2
Synthesis of results	21	Present results of each meta-analysis done, including confidence intervals and measures of consistency.	n/a
Risk of bias across studies	22	Present results of any assessment of risk of bias across studies (see Item 15).	36+Figure 2
Additional analysis	23	Give results of additional analyses, if done (e.g., sensitivity or subgroup analyses, meta-regression [see Item 16]).	n/a
**DISCUSSION**			
Summary of evidence	24	Summarize the main findings including the strength of evidence for each main outcome; consider their relevance to key groups (e.g., healthcare providers, users, and policy makers).	44-45
Limitations	25	Discuss limitations at study and outcome level (e.g., risk of bias), and at review-level (e.g., incomplete retrieval of identified research, reporting bias).	46-47
Conclusions	26	Provide a general interpretation of the results in the context of other evidence, and implications for future research.	47-49
**FUNDING**			
Funding	27	Describe sources of funding for the systematic review and other support (e.g., supply of data); role of funders for the systematic review.	51

From: Moher D, Liberati A, Tetzlaff J, Altman DG, The PRISMA Group (2009). Preferred Reporting Items for Systematic Reviews and Meta-Analyses: The PRISMA Statement. PLoS Med 6(7): e1000097. doi:10.1371/journal.pmed1000097

For more information, visit: **www.prisma-statement.org**.

For peer review only - http://bmjopen.bmj.com/site/about/guidelines.xhtml

PRISMA 2009 Checklist

For peer review only
